# Deregulated microRNA and mRNA expression profiles in the peripheral blood of patients with Marfan syndrome

**DOI:** 10.1186/s12967-018-1429-3

**Published:** 2018-03-12

**Authors:** Masood Abu-Halima, Mustafa Kahraman, Dominic Henn, Tanja Rädle-Hurst, Andreas Keller, Hashim Abdul-Khaliq, Eckart Meese

**Affiliations:** 1 Institute of Human Genetics, Saarland University, 66421 Homburg/Saar, Germany; 2 Chair for Clinical Bioinformatics, Saarland University, 66041 Saarbrücken, Germany; 3 Department of Hand, Plastic and Reconstructive Surgery, BG Trauma Center Ludwigshafen, University of Heidelberg, 67071 Ludwigshafen, Germany; 4 Department of Pediatric Cardiology, Saarland University Medical Center, 66421 Homburg/Saar, Germany

**Keywords:** MicroRNA, mRNA, Integration analysis, Marfan syndrome, Fibrillin

## Abstract

**Background:**

MicroRNAs (miRNAs) are small RNAs regulating gene expression post-transcriptionally. While acquired changes of miRNA and mRNA profiles in cancer have been extensively studied, little is known about expression changes of circulating miRNAs and messenger RNAs (mRNA) in monogenic constitutional anomalies affecting several organ systems, like Marfan syndrome (MFS). We performed integrated miRNA and mRNA expression profiling in blood samples of Marfan patients in order to investigate deregulated miRNA and mRNA networks in these patients which could serve as potential diagnostic and prognostic tools for MFS therapy.

**Methods:**

MiRNA and mRNA expression profiles were determined in blood samples from MFS patients (n = 7) and from healthy volunteer controls (n = 7) by microarray analysis. Enrichment analyses of altered mRNA expression were identified using bioinformatic tools.

**Results:**

A total of 28 miRNAs and 32 mRNAs were found to be significantly altered in MFS patients compared to controls (> 2.0-fold change, adjusted *P *< 0.05). The expression of 11 miRNA and 6 mRNA candidates was validated by RT-qPCR in an independent cohort of 26 MFS patients and 26 matched HV controls. Significant inverse correlations were evident between 8 miRNAs and 5 mRNAs involved in vascular pathology, inflammation and telomerase regulation. Significant positive correlations were present for 7 miRNAs with age, for 2 miRNAs with the MFS aortic root status (Z-score) and for 7 miRNAs with left ventricular end-diastolic diameter in MFS patients. In addition, miR-331-3p was significantly up-regulated in MFS patients without mitral valve prolapse (MVP) as compared with patients with MVP.

**Conclusions:**

Our data show deregulated gene and miRNA expression profiles in the peripheral blood of MFS patients, demonstrating several candidates for prognostic biomarkers for cardiovascular manifestations in MFS as well as targets for novel therapeutic approaches. A deregulation of miRNA expression seems to play an important role in MFS, highlighting the plethora of effects on post-transcriptional regulation of miRNAs and mRNAs initiated by constitutional mutations in single genes.

*Trial registration* Nr: EA2/131/10. Registered 28 December, 2010

**Electronic supplementary material:**

The online version of this article (10.1186/s12967-018-1429-3) contains supplementary material, which is available to authorized users.

## Background

Marfan syndrome (MFS, OMIM #154700) is a connective tissue disorder with an estimated incidence of 1:5000 individuals, across all ethnic backgrounds [1, 2]. Although an autosomal dominant inheritance of MFS typically appears in affected multi-generation families, approximately 25% of cases have the disorder as the result of a de novo mutation [3]. The phenotypic variability of this disorder ranges from minor stigmata to life-threatening manifestations, typically involving the cardiovascular (thoracic aortic aneurysms (TAA) and dissections), ocular (ectopia lentis), and musculoskeletal system (tall stature with arachnodactyly) [4–6]. Cardiovascular manifestations are responsible for the high morbidity in individuals with MFS and can include dilation of the ascending aorta, pulmonary artery dilation, and mitral valve prolapse [6]. Mutations in Fibrillin-1 (FBN1) were identified as the cause of MFS [5]. As FBN1 is a constituent of the connective tissue in a wide range of organs, decreased mechanical stability caused by mutations in FBN1 has pleiotropic effects. Pleiotropy was introduced by Plate in 1910 to describe multiple phenotypic effects of a single genetic trait [7]. Although there are 3077 known mutations in the FBN1 gene (UMD-FBN1 listed in the database: http://www.umd.be/FBN1/, updated on August 28, 2014) and more than 1500 different disease-causing FBN1 mutations, there is no single FBN1 genotype feature that qualifies as a reliable predictor of the clinical severity and long-term course of MFS. Even within a given family with an identical FBN1 mutation, there is considerable variation as to the severity of manifestation, pointing towards complex interactions of FBN1 with other genes and their products [4, 6, 8]. Currently, methods for predicting the prognosis of Marfan-related cardiovascular manifestations are limited. However, in several pathologies, microRNAs (miRNAs) have emerged in recent years as a promising new type of biomarker for the prognosis of disease, including initial data on MFS and aortic disease [9, 10]. MiRNAs are a class of non-coding RNAs of 18–22 nucleotides in length that regulate gene expression post-transcriptionally via sequence-specific interaction with the 3′ untranslated region (UTR) of a target gene’s mRNA, resulting in inhibition of translation and/or mRNA degradation [7]. Altered expression of miRNA has been associated with many human diseases, including MFS [9, 11]. Recently, it was reported that miR-29b is associated with vascular remodeling and subsequent aneurysm development characteristic of MFS and that this miRNA plays an important role in regulating aortic wall apoptosis and extracellular matrix abnormalities in MFS [11]. In addition to miRNA expression analysis, genome-wide mRNA expression analyses of skin fibroblast cultures from individuals with known FBN1 mutations and controls has been performed [12]. In tissue of MFS patients, however, investigations of miRNAs as well as mRNAs are still lacking. Thus, it is conceivable that in addition to an entire miRNome expression profiling, the search for miRNAs whose expression inversely correlates with the expression of mRNA targets may demonstrate another layer of the molecular diversity of this pleiotropic syndrome and may potentially be a useful diagnostic and prognostic tool for MFS therapy and treatment. A crucial clinical challenge are still insufficient indication criteria for preventive aortic replacement that call for biological parameters beyond the current restriction to ultrasonographic and magnetic resonance imaging (MRI) measurements. Therefore, we investigated differences in miRNA and mRNA expression patterns between MFS patients and healthy volunteer [13] controls. We furthermore performed an integrated analysis across all samples to identify mRNA targets of deregulated miRNAs. To our knowledge, this is the first large-scale investigation of the association between miRNA-related mRNAs in patients with MFS.

## Methods

### Patient samples

The study was conducted in accordance with the Declaration of Helsinki and approved by the locally appointed Ethics committee [Institutional Review Board (Number: EA2/131/10)]. Informed consent was obtained from all patients and HV controls. A cohort of 34 patients in whom the clinical diagnosis of classical MFS was made according to the current Ghent nosology [6] was assessed for the ocular, musculoskeletal, and cardiovascular features by an ophthalmologist, a pediatrician, a cardiologist, and a clinical geneticist. Two-dimensional echocardiography was used to measure the diameter of the ascending aorta which was used to determine the patients’ Z-score. Moreover the left ventricular end-diastolic diameter (LVEDD) and the presence of a mitral valve prolapse (MVP) were assessed by echocardiography. The patient cohort included 15 males and 19 females with a mean age of 27.62 years (standard deviation ± 15.66 years) and confirmed FBN1 mutation which were compared with age- and sex matched HV controls (n = 34). All Marfan patients were on Angiotensin receptor blockers. Beta blockers or ACE inhibitors had been added to the medication depending on the level of arterial hypertension or the presence of other cardiovascular morbidities. In all HV controls, a physical examination including measurement of blood pressure and transcutaneous oxygen saturation as well as two-dimensional echocardiography was performed to rule out any confounding cardiac and extracardiac abnormalities. At the time of enrolment, none of the controls took any medication or had elevated blood pressure. Additionally, none of them had any heart abnormality on the echocardiogram. In all patients and HV controls 2.5 mL of venous blood from the cubital vein was collected in PAXgene blood tubes (BD Biosciences, San Jose, California, United States). All PAXgene blood tubes were stored at room temperature for 2 h to ensure complete lysis of the blood cells before they were stored at − 20 °C until RNA isolation. MiRNA raw data were acquired from samples which had previously been used for a related study published by our group [9].

### RNA isolation

Total RNA including miRNAs of all MFS patients and HV controls was isolated with the PAXgene miRNA blood kit using the QIAcube™ automated isolation instrument according to the manufacturer’s instructions (Qiagen, Hilden, Germany). The RNA concentration and purity were confirmed by the spectrophotometric ratio using absorbance measurements at wavelengths of 260 and 280 nm on a NanoDrop-2000 (Thermo Scientific, Waltham, Massachusetts, United States). The integrity of the isolated RNA was analyzed on a RNA Nano 6000 chip using an Agilent Bioanalyzer (Agilent Technologies, Santa Clara, California, United States). Genomic DNA contamination was removed, and conventional polymerase chain reaction (PCR) was carried out to exclude any residual DNA in the samples as previously described [14]. Moreover, the RNA-based RT-qPCRs were carried out using predesigned exon spanning primers (Qiagen).

### Gene expression microarray assay analysis

MRNA expression profiles of MFS (n = 8) and HV controls (n = 8) samples were performed with SurePrint G3 Human Gene Expression v2 8x60K microarrays containing 50,599 biological features (Agilent Technologies, Santa Clara, CA, United States). All procedures were carried out according to the manufacturer’s protocol. Briefly, 100 ng total RNA from each sample was reversely transcribed, amplified and labeled using the LowInput QuickAmp Labeling Kit (Agilent). Quantification and specific activity of labeled complementary RNA (cRNA) was evaluated using the NanoDrop-2000 spectrophotometer (Thermo Scientific) to ensure that labeled cRNA was of sufficient quality for hybridization. A total of 600 ng of cRNA was then applied to the microarray slide per the manufacturer’s instructions and hybridized in a rotating oven for 17 h at 65 °C and 10 rpm. Arrays were washed and then scanned using a DNA Microarray Scanner (Agilent). Feature extraction software was utilized to extract gene expression data (Agilent).

### MiRNA microarray assay analysis

We used the Sureprint G3 Human miRNA 8x60K microarrays raw data of 8 MFS and 8 HV controls [9]. MiRNA expression profiles were performed with Sureprint G3 Human miRNA 8x60K microarrays containing 40 replicates of 1205 miRNAs of miRBase v16 (Agilent). All procedures were carried out according to the manufacturer’s protocol. Briefly, 100 ng total RNA from each sample was processed using the miRNA Complete Labeling and Hyb Kit (Agilent) to generate fluorescently labeled miRNA. The labeled RNA was then applied to the microarray slide per the manufacturer’s instructions and hybridized in a rotating oven for 20 h at 55 °C and 20 rpm. Arrays were washed and then scanned using a DNA Microarray Scanner (Agilent). Feature extraction software was utilized to extract gene expression data (Agilent).

### Reverse transcription and quantitative real-time PCR

Expression of selected mRNAs and miRNAs in MFS and HV controls was determined by real-time quantitative PCR (RT-qPCR) using the StepOnePlus™ Real-Time PCR System (Applied Biosystems, Foster City, CA, United States) and the miScript PCR System that contain *mi*Script RT II Kit with 5× miScript HiFlex Buffer and SYBR Green PCR along with the QuantiTect and miScript Primer Assays (Qiagen). All procedures were carried out according to the manufacturer’s recommendations. Using a cohort of independent MFS patients (n = 26) and HV controls (n = 26), 13 differentially expressed mRNAs (CLU, CRYAA, CTNNA1, DYSF, GBP2, ITGB3, LIMK2, MFN2, MMP9, MX1, SIRPB1, POT1 and SOCS3) and 18 differentially expressed miRNAs (miR-1228, miR-1234-3p, miR-1275, miR-139-3p, miR-151-5p, miR-200c, miR-24, miR-30e, miR-324-5p, miR-940, miR-3616-3p, miR-362-5p, miR-500b, miR-502-3p, miR-564, miR-627, miR-874 and miR-331-3p) were selected to validate the array results. In brief, 400 ng of total RNA were converted into complementary DNA (cDNA). The resulting cDNA was then diluted to have 1.5 and 0.5 ng/µL for mRNA and miRNA, respectively. All RT-qPCR experiments were performed using the Liquid Handling Robot QIAgility™ (Qiagen) before performing RT-qPCR. *β*-Actin and RNU6B small nuclear RNA (snRNA) primer assays (Qiagen) were chosen as endogenous references for mRNA and miRNA normalization. Moreover, a no template control (NTC) and no reverse transcriptase control (RT negative) were included in each mRNA and miRNA in each run, and a miRNA reverse transcription control (miRTC) was performed to assess the performance of the reverse transcription reaction by detecting template synthesized from the kit’s built-in control RNA (Qiagen). Melting curve analysis was used to control for the specificity of RT-qPCR products.

### Overrepresentation analysis

To evaluate the significance of the identified differentially expressed genes, the Protein ANalysis THrough Evolutionary Relationships (PANTHER) Classification System was used to categorize the differentially expressed genes according to PANTHER protein class, Gene Ontology (GO) Molecular Function, GO Biological Process and GO cellular components annotations [15]. For each biological pathway and/or process, the difference between the observed fraction of genes in that pathway and/or process and the number expected by chance was tested using Fisher’s exact test. *P* values were adjusted using a Bonferroni correction.

### Statistical analysis

The statistical analysis was performed using R (version 3.4.0) to analyze the differences in mRNA and miRNA expression patterns in the MFS patients and HV controls. Raw data generated by the Agilent Feature Extraction image analysis software was normalized by variance stabilizing normalization (vsn) [16] and quantile normalization methods for mRNAs and miRNAs, respectively and uploaded to the NCBI GEO database (Accession ID: GSE110966). The significance level of mRNAs and miRNAs was determined by applying an unpaired two-tailed t test. Then the median values of each miRNA and mRNA were log2 transformed and the resulting miRNA P values were adjusted for multiple testing using Benjamini–Hochberg adjustment. In addition, the area under the receiver operating characteristic curve values for each miRNA were computed. For the significantly deregulated miRNAs and protein coding genes with *P *< 0.05 and fold change > 2 or < 1/2 in MFS patients compared to HV controls, we computed a Pearson correlation coefficient of expression for each mRNA–miRNA pair. Spearman’s correlations coefficient was used to correlate the clinical parameters of MFS and the expression level of both validated miRNAs and mRNAs. Using the DataAssist™ Software v3.0 (Applied Biosystems), the fold-change and *P* value (unpaired t test with Welch’s correction) of each mRNA and miRNA was calculated.

## Results

### Patient characteristics

Among 19 females and 15 males included in the study, there were 22 patients with MVP, 6 patients with ectopia lentis and 14 patients who underwent aortic root replacement because of aortic dissection or an aortic aneurysm (aortic root > 53 mm). Additional file 3: Table S1 shows the clinical features of the MFS patients. The presented data refer to the largest diameters of the aortic root before surgery.

### Correlation analysis of miRNA and mRNA between MFS patients and HV controls

As an initial analysis, we calculated the degree of correlation based on Pearson’s correlation coefficient across samples from each group, i.e., MFS patients and HV controls. The correlation plots are presented in Additional file 1: Figure S1 for miRNA and Additional file 2: Figure S2 for mRNA. In general, the correlation heatmaps illustrated that the correlation was strong (Pearson correlation coefficient r of mostly > 0.80 and > 0.92 for miRNA and mRNA, respectively) in both MFS patients and HV controls, except for two samples which we identified as outliers in the mRNA data by applying Hampel’s rule for outlier detection [17]. These two samples and the corresponding miRNA samples of the same MFS patients and HV controls were excluded from further analyses.

### Differentially expressed miRNAs between MFS patients and HV controls

**Fig. 1:**
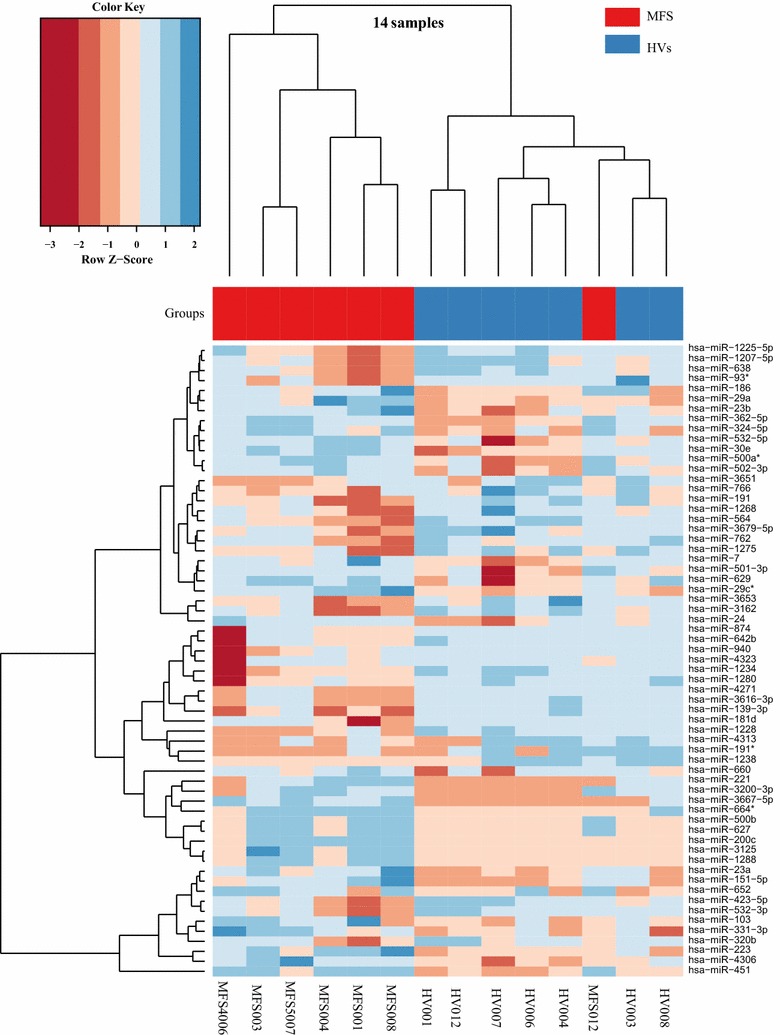
Unsupervised hierarchical clustering (Euclidian distance, complete linkage) of the 14 samples based on expression of the 63 with significant highest variance out of the 1205 miRNAs. The heatmap shows miRNAs with high expression in red, miRNAs with low expression in green. The red lines indicate three main clusters of samples

Using the high-throughput SurePrint G3 Human v16 miRNA microarray platform, we screened the expression level of 1205 human mature miRNAs of miRBase v16. Following background correction and normalization, the miRNA expression levels from MFS patients and HV controls were identified. After excluding outliers, 7 MFS patients and 7 HV controls were considered for further analysis. Using quantile normalization, a total of 277 miRNAs were detected in at least 25% of the samples in at least one group (filtering). By applying an un-paired two-tailed t test for miRNAs that showed a significant change in the considered groups, 63 miRNAs showed statistical significance in MFS patients versus HV controls (Table 1) (P < 0.05. FDR adjusted). By considering only the differentially expressed miRNAs with twofold or greater change in MFS patients versus HV controls, a total of 28 miRNAs were identified including 15 down-regulated and 13 up-regulated miRNAs (*P* value < 0.05, fold change ≥ 2.0) (Table 1). To compare the relative expression level of the differentially expressed miRNAs, we used the hierarchical clustering of miRNAs and hierarchical clustering of samples based on average linkage and Euclidian distance of the significantly deregulated 63 miRNAs out of 1205 miRNAs in MFS patients versus HV controls (Fig. 1). In general, hierarchical clustering revealed that MFS patients and HV controls were grouped into two distinct clusters, except for only one MFS (fell into the wrong cluster). Moreover, the heatmap showed that some miRNAs were expressed only in the MFS patients group and/or expressed at a low level in HV controls and vice versa (Fig. 1).

**Table 1:** Significantly expressed miRNAs in the blood of patients with MFS (n = 7) compared HVs controls (n = 7) as determined by microarray (unpaired two-tailed t test. P < 0.05, FDR adjusted)

miRNA	Median log2 MFS	Median log2 HVS	Fold change	Log2 fold change	Regulation	P value	Adjusted P value	AUC
hsa-miR-4271	− 3.32	4.40	0.0047	− 7.72	Down	0.0037	0.0689	0.95
hsa-miR-3616-3p	− 3.32	4.15	0.0056	− 7.47	Down	0.0003	0.0149	0.96
hsa-miR-1228	− 3.32	3.80	0.0072	− 7.12	Down	0.0004	0.0149	0.98
hsa-miR-1238	− 3.32	3.17	0.0111	− 6.49	Down	0.0169	0.1552	0.84
hsa-miR-191	− 3.32	3.03	0.0123	− 6.35	Down	0.0450	0.2177	0.80
hsa-miR-1234	0.94	4.02	0.12	− 3.09	Down	0.0004	0.0149	0.98
hsa-miR-4313	0.69	3.39	0.15	− 2.70	Down	0.0468	0.2177	0.78
hsa-miR-139-3p	0.65	3.15	0.18	− 2.50	Down	0.0001	0.0149	1.00
hsa-miR-874	2.83	4.76	0.26	− 1.92	Down	0.0042	0.0731	0.92
hsa-miR-564	4.70	5.90	0.43	− 1.20	Down	0.0003	0.0149	1.00
hsa-miR-940	3.06	4.21	0.45	− 1.15	Down	0.0004	0.0149	1.00
hsa-miR-1207-5p	5.80	6.93	0.46	− 1.13	Down	0.0319	0.1878	0.83
hsa-miR-1280	1.64	2.73	0.47	− 1.10	Down	0.0048	0.0790	0.98
hsa-miR-181d	3.93	4.99	0.48	− 1.06	Down	0.0277	0.1845	0.80
hsa-miR-1275	3.77	4.80	0.49	− 1.03	Down	0.0014	0.0323	0.94
hsa-miR-3653	7.04	8.01	0.51	− 0.97	Down	0.0484	0.2177	0.85
hsa-miR-1268	5.18	6.07	0.54	− 0.88	Down	0.0239	0.1739	0.89
hsa-miR-320b	10.66	11.48	0.57	− 0.82	Down	0.0085	0.1242	0.92
hsa-miR-532-3p	8.69	9.46	0.59	− 0.77	Down	0.0130	0.1444	0.84
hsa-miR-642b	3.17	3.93	0.59	− 0.76	Down	0.0179	0.1552	0.87
hsa-miR-4323	2.73	3.44	0.61	− 0.70	Down	0.0218	0.1675	0.87
hsa-miR-93	5.67	6.31	0.64	− 0.64	Down	0.0297	0.1871	0.80
hsa-miR-3162	6.83	7.46	0.65	− 0.63	Down	0.0068	0.1050	0.89
hsa-miR-3679-5p	4.53	5.13	0.66	− 0.59	Down	0.0278	0.1845	0.87
hsa-miR-423-5p	9.11	9.62	0.70	− 0.51	Down	0.0124	0.1433	0.86
hsa-miR-1225-5p	6.16	6.67	0.70	− 0.51	Down	0.0287	0.1852	0.84
hsa-miR-3651	4.99	5.44	0.73	− 0.45	Down	0.0478	0.2177	0.81
hsa-miR-638	5.98	6.41	0.74	− 0.43	Down	0.0193	0.1575	0.86
hsa-miR-766	4.84	5.25	0.76	− 0.40	Down	0.0238	0.1739	0.91
hsa-miR-191	5.10	5.49	0.76	− 0.39	Down	0.0350	0.1978	0.83
hsa-miR-762	4.55	4.73	0.88	− 0.18	Down	0.0403	0.2106	0.78
hsa-miR-221	2.77	− 3.32	68.19	6.09	Up	0.0499	0.2177	0.18
hsa-miR-1288	2.53	− 3.32	57.95	5.86	Up	0.0344	0.1978	0.21
hsa-miR-3125	2.35	− 3.32	50.81	5.67	Up	0.0481	0.2177	0.21
hsa-miR-500b	2.27	− 3.32	48.35	5.60	Up	0.0142	0.1462	0.14
hsa-miR-200c	2.13	− 3.32	43.66	5.45	Up	0.0142	0.1462	0.14
hsa-miR-3200-3p	2.07	− 3.32	41.98	5.39	Up	0.0280	0.1845	0.17
hsa-miR-3667-5p	1.81	− 3.32	35.05	5.13	Up	0.0380	0.2067	0.14
hsa-miR-627	1.74	− 3.32	33.47	5.06	Up	0.0311	0.1878	0.14
hsa-miR-664	1.33	− 3.32	25.09	4.65	Up	0.0395	0.2106	0.20
hsa-miR-223	13.58	11.98	3.04	1.60	Up	0.0496	0.2177	0.15
hsa-miR-660	4.28	2.92	2.57	1.36	Up	0.0192	0.1575	0.08
hsa-miR-29c	4.59	3.33	2.41	1.27	Up	0.0177	0.1552	0.00
hsa-miR-7	4.62	3.56	2.09	1.06	Up	0.0357	0.1978	0.12
hsa-miR-29a	6.93	5.98	1.93	0.95	Up	0.0117	0.1407	0.04
hsa-miR-500a	5.70	4.80	1.87	0.90	Up	0.0008	0.0202	0.04
hsa-miR-23a	9.46	8.57	1.85	0.89	Up	0.0103	0.1364	0.06
hsa-miR-151-5p	9.11	8.33	1.73	0.79	Up	0.0175	0.1552	0.09
hsa-miR-324-5p	6.20	5.41	1.72	0.79	Up	0.0005	0.0149	0.05
hsa-miR-4306	12.57	11.84	1.66	0.73	Up	0.0161	0.1552	0.08
hsa-miR-186	6.78	6.10	1.60	0.68	Up	0.0261	0.1845	0.12
hsa-miR-502-3p	5.53	4.87	1.58	0.66	Up	0.0005	0.0149	0.00
hsa-miR-23b	6.34	5.70	1.56	0.64	Up	0.0468	0.2177	0.18
hsa-miR-629	4.76	4.12	1.56	0.64	Up	0.0466	0.2177	0.20
hsa-miR-362-5p	6.34	5.74	1.52	0.60	Up	0.0005	0.0149	0.02
hsa-miR-652	9.07	8.47	1.51	0.60	Up	0.0116	0.1407	0.16
hsa-miR-24	7.95	7.38	1.48	0.57	Up	0.0018	0.0392	0.01
hsa-miR-501-3p	4.31	3.74	1.48	0.56	Up	0.0099	0.1364	0.04
hsa-miR-30e	5.97	5.49	1.39	0.48	Up	0.0027	0.0542	0.08
hsa-miR-331-3p	10.09	9.62	1.38	0.46	Up	0.0205	0.1621	0.16
hsa-miR-451	16.51	16.05	1.38	0.46	Up	0.0004	0.0149	0.06
hsa-miR-532-5p	5.94	5.53	1.33	0.41	Up	0.0318	0.1878	0.13
hsa-miR-103	10.66	10.25	1.33	0.41	Up	0.0478	0.2177	0.18

Each value represents the median of 7 MFS patients and 7 HV controls and ± standard deviation (STDV). Statistical analysis was performed with unpaired-two-tailed t test (P < 0.05). MFS Marfan syndrome, HVs healthy volunteers; AUC area under the receiver operating characteristic curve

### Differentially expressed genes between MFS patients and HV controls

**Fig. 2:**
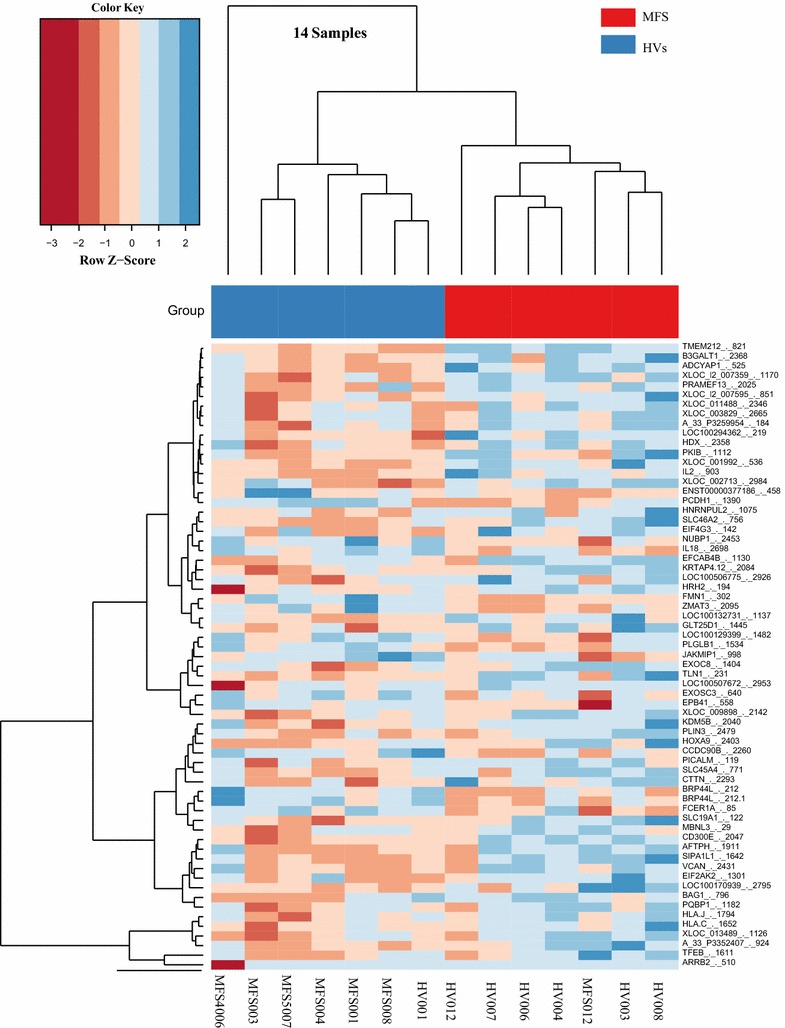
Unsupervised hierarchical clustering (Euclidian distance, complete linkage) of the 14 samples based on expression of the 65 transcripts with the highest expression variances out of the 50,599 biological features. The heatmap shows transcripts with high expression in red, transcript with low expression in green. The red lines indicate three main clusters of samples

Using the high-throughput SurePrint G3 Human Gene Expression v2 microarray platform, we screened the expression level of human 50,599 biological features of ENSEMBL release 52. Following background correction and normalization, the gene expression levels from MFS patients and HV controls were identified. Using variance stabilizing normalization (vsn) to the generated gene expression data, 58,717 transcripts were detected (no filtering). By applying an un-paired two-tailed t test for the transcripts that showed a significant change in the considered groups, we found 1662 transcripts with significant differences of MFS patients versus HV controls (P < 0.05) (Additional file 3: Table S2). By considering only the differentially expressed transcripts with 1.5-fold or greater change in MFS patients versus HV controls, a total of 505 transcripts were identified including 15 down-regulated and 490 up-regulated transcripts (*P* value < 0.05, fold change ≥ 1.5). Considering only the protein coding genes and removing different transcript variants, 296 genes out of 505 transcripts were identified, including 5 down-regulated and 291 up-regulated protein coding genes (Additional file 3: Table S3). The number of significant protein coding genes with twofold or greater change in MFS patients versus HV controls was decreased including one down-regulated and 31 up-regulated genes (*P* value < 0.05) (Table 2). Using hierarchical clustering with the Euclidian distance measure, we analyzed how the MFS patients and HV controls relate to each other. For this task, we used the 65 transcripts with the highest expression variances out of the 50,599 biological features. Figure 2 shows the resulting heatmap of the hierarchical clustering. In general, we observed two distinct clusters without overlap, with the first cluster containing only HV controls and the second cluster containing only MFS patients.

**Table 2:** Significantly expressed protein coding genes in the blood of patients with MFS (n = 7) compared HVs controls (n = 7) as determined by microarray (unpaired two-tailed t test. > 2.0-fold difference. P < 0.05)

Gene Name	NCBI accession code	Median log2 MFS	Median log2 HVS	Fold change	log2 Fold change	Regulation	P value	AUC
POT1 (protection of telomeres 1)	NM_015450	7.96	9.05	0.47	−1.09	Down	0.0002	1.00
SIRPB1 (signal regulatory protein beta 1)	NM_001135844	11.30	8.96	5.08	2.34	Up	0.0474	0.16
HBZ (hemoglobin subunit zeta)	NM_005332	8.71	6.47	4.73	2.24	Up	0.0132	0.16
MYOM2 (myomesin 2)	NM_003970	8.38	6.50	3.67	1.87	Up	0.0372	0.14
ITGB3 (integrin subunit beta 3)	NM_000212	9.23	7.38	3.62	1.86	Up	0.0392	0.08
MX1 (MX dynamin like GTPase 1)	NM_002462	12.89	11.36	2.89	1.53	Up	0.0383	0.16
DYSF (dysferlin)	NM_003494	10.49	9.04	2.74	1.45	Up	0.0149	0.08
CCR1 (C-C motif chemokine receptor 1)	NM_001295	12.91	11.50	2.66	1.41	Up	0.0217	0.12
IFIT2 (interferon induced protein with tetratricopeptide repeats 2)	NM_001547	11.56	11.33	1.17	0.23	Up	0.0317	0.14
LRG1 (leucine rich alpha-2-glycoprotein 1)	NM_052972	11.14	9.85	2.45	1.29	Up	0.0181	0.16
TRANK1 (tetratricopeptide repeat and ankyrin repeat containing 1)	NM_014831	10.86	9.62	2.37	1.25	Up	0.0013	0.02
ZAN (zonadhesin (gene/pseudogene))	NM_173059	9.30	8.08	2.34	1.23	Up	0.0423	0.18
CRYAA (crystallin alpha A)	NM_000394	9.30	8.08	2.34	1.23	Up	0.0329	0.18
NRGN (neurogranin)	NM_006176	12.69	11.52	2.25	1.17	Up	0.0356	0.12
RNF213 (ring finger protein 213)	NM_020914	8.24	7.70	1.46	0.55	Up	0.0377	0.18
LIMK2 (LIM domain kinase 2)	NM_001031801	9.31	8.16	2.22	1.15	Up	0.0181	0.14
MFN2 (mitofusin 2)	NM_014874	5.49	5.18	1.24	0.31	Up	0.0288	0.20
CTTN (cortactin)	NM_005231	9.51	8.36	2.21	1.14	Up	0.0268	0.16
MX2 (MX dynamin like GTPase 2)	NM_002463	12.60	11.52	2.11	1.07	Up	0.0157	0.12
CD14 (CD14 molecule)	NM_001174104	15.04	13.97	2.10	1.07	Up	0.0332	0.16
GRN (granulin precursor)	NM_002087	15.83	14.76	2.10	1.07	Up	0.0258	0.16
RNF222 (ring finger protein 222)	NM_001146684	5.29	5.01	1.22	0.28	Up	0.0336	0.14
MVP (major vault protein)	NM_017458	14.55	13.49	2.08	1.06	Up	0.0046	0.08
CXCL5 (C-X-C motif chemokine ligand 5)	NM_002994	8.88	7.83	2.07	1.05	Up	0.0455	0.18
CTNNA1 (catenin alpha 1)	NM_001903	9.08	8.05	2.05	1.04	Up	0.0309	0.16
MMP9 (matrix metallopeptidase 9)	NM_004994	13.20	12.14	2.07	1.05	Up	0.0456	0.20
SOCS3 (suppressor of cytokine signaling 3)	NM_003955	9.47	8.43	2.05	1.03	Up	0.0472	0.20
GBP2 (guanylate binding protein 2)	NM_004120	11.49	10.46	2.04	1.03	Up	0.0097	0.08
GNL3L (G protein nucleolar 3 like)	NM_019067	8.33	7.93	1.32	0.41	Up	0.0325	0.20
CLU (clusterin)	NM_001831	9.71	8.69	2.02	1.02	Up	0.0423	0.22
SELL (selectin L)	NM_000655	14.93	13.93	2.00	1.00	Up	0.0011	0.04
OSM (oncostatin M)	NM_020530	9.18	8.19	2.00	1.00	Up	0.0414	0.14

Each value represents the median of 7 MFS patients and 7 HV controls and ± standard deviation (STDV). Statistical analysis was performed with unpaired-two-tailed t test (*P *< 0.05). *MFS* Marfan syndrome, *HVs* healthy volunteers; AUC area under the receiver operating characteristic curve

### Validation of candidate miRNAs and mRNAs by RT-qPCR

**Fig. 3:**
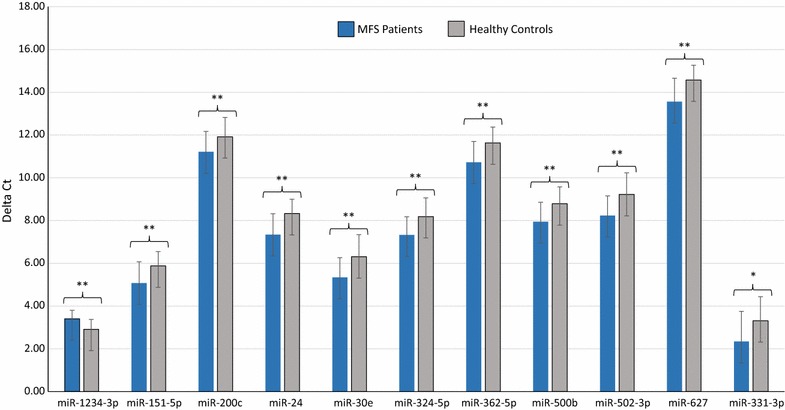
Validation of differentially expressed miRNAs in the blood of MFS patients (n = 26) compared to HV controls (n = 26) as determined by RT-qPCR (P < 0.05). Mean ΔCt MFS and HV controls (Lower ΔCt, higher expression level). RNAU6B as an endogenous control for normalization, Unpaired-two-tailed t tests and ± standard deviation (STDV) were used to evaluate differences in expression. *P ≤ 0.05; **P ≤ 0.01; ***P ≤ 0.001

**Fig. 4:**
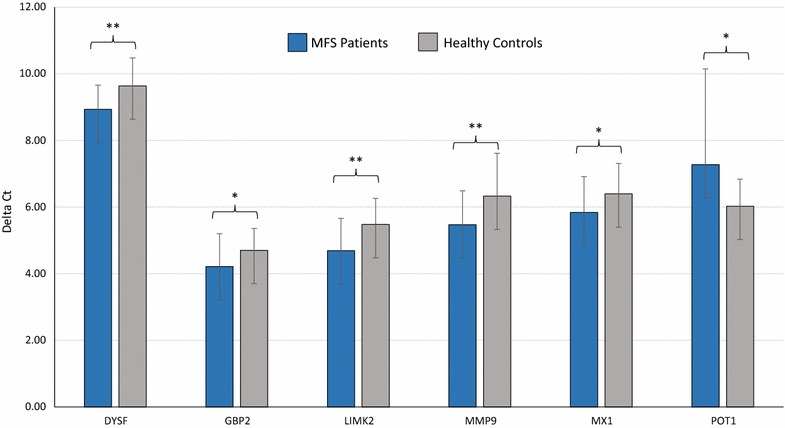
Validation of differentially expressed mRNAs in the blood of MFS patients (n = 26) compared to HV controls (n = 26) as determined by RT-qPCR (P < 0.05). Mean ΔCt MFS and HV controls (Lower ΔCt, higher expression level). β-Actin as an endogenous housekeeping gene for normalization, Unpaired-two-tailed t tests and ± standard deviation (STDV) were used to evaluate differences in expression. *P ≤ 0.05; **P ≤ 0.01; ***P ≤ 0.001

In order to validate the results obtained from the microarray analysis, RT-qPCR was performed using a larger separate cohort of MFS patients and HV controls (MFS patients, n = 26 and HV controls, n = 26). Eighteen miRNAs were selected based on their differential expression level in MFS patients versus HV controls, and some of them were selected based on their known associations with cardiovascular diseases and MFS (listed in Table 1). In addition, 13 mRNAs were selected based on their known associations with cardiovascular diseases like mitral valve stenosis, myocardial infarction, ischemia and acute coronary syndrome, and some had been observed to be differentially expressed with twofold or greater change in MFS patients versus HV controls in the microarray data (listed in Table 2). Considering the direction of expression changes, there was a general accordance between microarray and RT-qPCR data for the miRNAs and mRNAs tested. In detail, RT-qPCR validated the results of the microarray analysis for 11 out of 18 miRNAs with regards both to the direction of expression changes and to the significance of different expressions between MFS patients and HV controls, including one significantly down-regulated miRNA (miR-1234-3p) and 10 significantly up-regulated miRNAs (miR-151-5p, miR-200c, miR-24, miR-30e, miR-324-5p, miR-362-5p, miR-500b, miR-502-3p, miR-627, and miR-331-3p) (Fig. 3). Likewise for the mRNA analysis, 11 out of 13 mRNAs showed the same direction of expression changes in the RT-qPCR and in the microarray analysis. Of these 11 miRNAs, 6 mRNAs were validated with regard to both the direction of expression changes and to the significance of different expressions between MFS patients and HV controls including 5 significantly up-regulated mRNAs (DYSF, GBP2, LIMK2, MMP9, and MX1) and one significantly down-regulated mRNA (POT1) (Fig. 4).

### Inverse correlation between miRNA and target mRNA

To further understand the relationship between miRNA and mRNA changes, and to specifically identify potentially relevant miRNA–mRNA target interactions, we calculated the Pearson correlation coefficient for every stably expressed miRNA and every protein coding gene that was significantly deregulated with a fold change of < 0.5/> 1.5. This computation yielded 11 significant negative combinations (P < 0.05) with a correlation range between − 0.62 and − 0.89 (Table 3). MiR-1234-3p showed the highest number of significant correlations followed by an intermediate group consisting of miR-324-5p, miR-151-5p, miR-200c-3p, miR-200c-3p, miR-362-5p, miR-502-3p and miR-627-5p. A number of genes involved in mitral valve stenosis, myocardial infection, ischemia and acute coronary syndrome exhibited a statistically significantly correlation between miRNA and mRNA expression. In addition, based on microarray data and RT-qPCR, miR-1234-3p was down-regulated and LIMK2, DYSF, GBP2, and MMP9 were up-regulated whereas one mRNA (POT1) was down-regulated and miR-324-5p, miR-151-5p, miR-200c-3p, miR-200c-3p, miR-362-5p, miR-502-3p and miR-627-5p were up-regulated.

**Table 3:** Significant negative correlation between the identified miRNA and mRNA by microarray (un-paired two-tailed t test < 0.5/> 1.5-fold difference, P < 0.05)

miRNA	mRNA	P value correlation	Correlation	Fold change miRNA	P value miRNA	Fold change mRNA	P value mRNA
hsa-miR-1234	LIMK2	0.00001	− 0.89	0.12	0.00038	2.22	0.01813
hsa-miR-1234	DYSF	0.00018	− 0.85	0.12	0.00038	2.74	0.01491
hsa-miR-1234	GBP2	0.00143	− 0.78	0.12	0.00038	2.04	0.00973
hsa-miR-1234	MMP9	0.00382	− 0.74	0.12	0.00038	2.15	0.03885
hsa-miR-324-5p	POT1	0.00670	− 0.69	1.72	0.00050	0.47	0.00017
hsa-miR-151-5p	POT1	0.00728	− 0.68	1.73	0.01754	0.47	0.00017
hsa-miR-200c	POT1	0.01132	− 0.65	43.66	0.01425	0.47	0.00017
hsa-miR-362-5p	POT1	0.01281	− 0.64	1.52	0.00045	0.47	0.00017
hsa-miR-502-3p	POT1	0.01590	− 0.63	1.58	0.00054	0.47	0.00017
hsa-miR-500b	POT1	0.01868	− 0.62	48.35	0.01419	0.47	0.00017
hsa-miR-627	POT1	0.01868	− 0.62	33.47	0.03109	0.47	0.00017

### Correlation between the clinical parameters and expression levels of miRNA and mRNA

We further analyzed the correlations between the validated 11 miRNAs and 6 mRNAs by RT-qPCR and various clinical parameters of MFS. We found that the expression levels of 7 miRNAs including miR-151-5p, miR-24, miR-30e, miR-324-5p, miR-362-5p, miR-500b, and miR-502-3p significantly correlated with the age of patients with MFS (Table 4) (*P *< 0.05). In contrast, no significant correlation was observed between the expression levels of these 7 miRNAs and the aortic root status of patients with MFS. However, there was a significant correlation between the expression level of miR-200c (P value = 0.015) and a borderline significant correlation with miR-151-5p, miR-324-5p and miR-500b and aortic root status (Z-score) of patients with MFS. Statistically significant correlations were observed between 7 miRNAs including miR-151-5p, miR-24, miR-30e, miR-324-5p, miR-500b, miR-502-3p, and miR-627) and the LVEDD. There was no significant correlation between the validated target mRNAs and the clinical parameters of MFS. Furthermore, we also assessed the significance of the differences of the validated 11 miRNAs in MFS patients without MVP compared with patients with MVP using the Wilcoxon test. Out of these 11 miRNAs, miR-331-3p showed a significant down-regulation in patients with MVP compared to patients without MVP (*P* < 0.05). MiR-200c showed a borderline significant decrease in MFS patients with MVP compared with patients without MVP (*P *= 0.060).

**Table 4:** Correlation between clinical parameters and validated miRNA and mRNA expression levels by RT-qPCR in patients with MFS P < 0.05)

Parameters	Marfan syndrome patients							Healthy volunteers	
	Age (MFS patients)		Aortic root status (Z score)		LVEDD		MVP	Age	
miRNA	Correlation	P value	Correlation	P value	Correlation	P value	P value*	Correlation	P value
hsa-miR-1234	0.003	0.988	0.063	0.761	− 0.075	0.716	0.732	− 0.122	0.554
hsa-miR-151-5p	0.519	*0.007*	0.375	***0.059***	0.496	*0.010*	0.979	0.055	0.789
hsa-miR-200c	0.370	***0.063***	0.473	*0.015*	0.213	0.295	0.060	− 0.220	0.279
hsa-miR-24	0.496	*0.010*	0.281	0.164	0.420	*0.033*	0.654	− 0.194	0.342
hsa-miR-30e	0.565	*0.003*	0.230	0.258	0.472	*0.015*	0.517	− 0.030	0.883
hsa-miR-324-5p	0.510	*0.008*	0.350	***0.079***	0.457	*0.019*	0.391	− 0.019	0.927
hsa-miR-362-5p	0.413	*0.036*	0.216	0.289	0.383	***0.053***	0.673	0.024	0.906
hsa-miR-500b	0.440	*0.024*	0.384	***0.053***	0.456	*0.019*	0.812	− 0.003	0.987
hsa-miR-502-3p	0.505	*0.008*	0.260	0.200	0.425	*0.030*	0.816	0.036	0.860
hsa-miR-627	0.310	0.123	0.291	0.149	0.423	*0.032*	0.816	0.010	0.961
hsa-miR-331-3p	0.307	0.127	0.333	0.097	0.015	0.942	0.041	− 0.243	0.231

Parameters	Marfan syndrome patients							Healthy volunteers	
	Age		Aortic root status (Z score)		LVEDD		MVP	Age	
mRNA	Correlation	P value	Correlation	P value	Correlation	P value	P value	Correlation	P value
DYSF	0.123	0.550	0.087	0.672	− 0.010	0.963	0.958	0.186	0.385
GBP2	0.200	0.326	0.071	0.729	− 0.198	0.332	0.816	0.080	0.710
LIMK2	0.003	0.989	− 0.142	0.489	− 0.241	0.235	0.916	0.135	0.530
MMP9	0.276	0.173	0.272	0.178	− 0.042	0.840	0.460	0.295	0.162
MX1	0.071	0.732	− 0.142	0.489	0.019	0.926	0.897	0.073	0.736
POT1	− 0.028	0.891	− 0.041	0.842	− 0.306	0.129	0.510	− 0.075	0.727

LVEDD left ventricular end diastolic diameter, MVP mitral valve prolapse. P values were calculated using unpaired-two-tailed t test (P < 0.05). *P values were calculated using Wilcoxon test (P < 0.05)

Italic—significant with adjusted P-value

Bold Italic—borderline significant with adjusted P-value

### Classification and overrepresentation analysis

Considering only the protein coding genes and removing of different transcript variants, 292 genes out of 296 were grouped according to PANTHER protein class, GO Molecular Function, GO Biological Process and GO cellular components annotations. The complete classifications can be found in the Additional file 3: Table S4. In detail, after applying Bonferroni correction for multiple testing, there were statistically significant pathways within the 292 genes differentially expressed in the MFS patients, displaying apoptosis signaling pathway (*P* value = 1.54E−03), JAK/STAT signaling pathway (*P* value = 1.78E−02), integrin signaling pathway (*P* value = 1.92E−02) and angiogenesis (*P* value = 3.50E−02) (Table 5).

**Table 5:** Pathways significantly enriched for the identified protein coding genes in the blood of patients with MFS compared to HV controls (adjusted P value < 0.05)

PANTHER classification pathways	Number of genes			Over-/under-represented (±)	Fold enrichment	P value
	Reference list^a^	Target list^b^	Expected^c^			
Apoptosis signaling pathway (P00006)	119	10	1.68	+	5.96	0.00154
JAK/STAT signaling pathway (P00038)	17	4	0.24	+	16.69	0.00210
Integrin signalling pathway (P00034)	194	11	2.73	+	4.02	0.01920
Angiogenesis (P00005)	174	10	2.45	+	4.08	0.03500

Pathways resulted significantly over-represented by the identified protein coding genes. P values were tested using Fisher exact test and adjusted using a Bonferroni correction test. MFS Marfan syndrome, HVs healthy volunteers

^a^ Number of genes in the reference list that map to this PANTHER classification category

^b^ Number of genes in the target genes list that map to this PANTHER classification category

^c^ Expected value is the number of genes that could be expected in target genes list for this PANTHER category based on the reference list

## Discussion

In this study, we found 13 miRNAs and 31 mRNAs with significantly increased expression levels and 15 miRNAs and one single mRNA (POT1) with significantly decreased expression level in patients with MFS compared with HV controls. In a cohort of independent MFS patients and HV controls, 11 miRNAs and 6 mRNAs were validated. These data show that miRNA and mRNA expression levels in the blood of patients with MFS differ from HV controls and that distinct differences in specific miRNA expression patterns can be further explored as potential biomarkers for differentiating between patients with MFS and HV controls. A distinctive non-invasive surrogate biomarker for MFS would be of high clinical value, as mutation analysis of the huge (65 exons) FBN1 gene is still relatively expensive and time-consuming and therefore restricted to phenotypically recognized Ghent-positive patients. An affordable screening test for MFS would likely detect a considerable number of atypical MFS who currently remain undiagnosed. Moreover, our investigation provides a comprehensive analysis of the gene expression pattern in patients with MFS as compared to HV controls, suggesting that non-pathogenic variants of other genes than FBN1 may significantly influence the phenotype, and explain the often striking clinical variation among members of a given MFS family. The identification of these genes may lead to a possible novel signature related to MFS, provide new prognostic parameters and ultimately even generate targets for novel approaches to chemoprevention of complications beyond currently unsatisfying medical treatment options [18]. Intriguingly, many of the biological pathways identified, such as apoptosis signaling [19], JAK/STAT signaling [20], integrin signaling [21] and angiogenesis pathways [22], have been associated with development of cardiovascular complications in MFS and its related diseases including aortic and pulmonary artery dilation as well as mitral valve prolapse. Among the identified deregulated mRNAs, some genes play a role in cardiomyocyte differentiation and remodeling during acute myocardial infarction and in dilated cardiomyopathy (DCM). For example, patients suffering from DCM show a strong and lasting increase of *oncostatin M* (OSM) gene expression level and signaling [23]. Moreover, significant changes in *clusterin* [24] gene level have been detected in patients with acute myocardial infarction (AMI) [25] and increased levels of *selectin L* (SELL) are associated with ischemic stroke [26]. The JAK/STAT pathway is negatively regulated by the suppressor of cytokine signaling (SOCS) protein, and the myocardium-specific suppressor of *cytokine signaling 3* (SOCS3) gene plays a key role in the development of left ventricular (LV) remodeling after AMI [27]. In agreement with the higher expression level of *C*-*X*-*C motif chemokine ligand 5* (CXCL5) in the blood of patients with MFS, CXCL5 showed an increased expression level in the plasma of patients with coronary artery disease. Recent studies have showed that CXCL5 and its receptors are implicated in congestive heart failure and ischemic stroke, making CXCL5 a candidate gene for potential future therapy strategies in cardiovascular diseases [28–30]. CXCL5 has also been reported to be up-regulated in abdominal aortic aneurysm (AAA) [31]. *Matrix metalloproteinase 9* (MMP) was shown to be up-regulated in the blood of MFS patients by microarray and RT-qPCR in our analysis. MMP9 showed a significant inverse correlation with hsa-miR-1234, which also was identified in the MFS patients [9]. A proteolytic degradation of the extracellular matrix of the aortic wall by an upregulation MMPs has been shown to be involved in the pathogenesis of TAA and AAA and also contributes to the histologic changes found in the aortic wall of patients with MFS [32, 33]. The expression of MMP9 has been shown to be up-regulated in the vascular wall of human AAA [34, 35] and also in aneurysm tissue in a mouse model of MFS [32]. Interestingly, Balistreri et al., found potential associations of SNPs in the MMP9 gene [rs3918242 (−1562C/T MMP-9)] degenerative forms of mitral valve diseases (MVDs), concluding that genetic variants in MMP9 play a role in MVD in MFS patients [36]. Together with our data, showing an up-regulation of MMP9 in the blood of MFS patients compared to controls, indicate that MMP9 may represent a potential biomarker and therapeutic target to reduce the growth rate of TAAs in MFS patients. *Doxycyclin* and statins have proven to be effective inhibitors of MMPs [37, 38] and have shown therapeutic benefits in both TAA and AAA patients [39, 40]. However, data on MFS patients as well as large randomized trials are still lacking, making these drugs promising candidates for future investigations in MFS. Our data, showing a significant inverse correlation of miR-1234 and MMP9 indicate that a down-regulation of this miRNA may be involved in the up-regulation MMP9 in MFS. We demonstrate a significantly up-regulated expression of the *LIM kinase 2* (LIMK2) which also inversely correlated with miR-1234. LIMK2 regulates dynamic changes of the actin cytoskeleton by phosphorylating *cofilin* and thereby inactivating its F-actin depolymerizing activity [41]. It was shown in mouse models that an activation of LIMK2 is associated with a disturbed flow in the aortic arch and disturbs endothelial cell (EC) barrier function, which was reversed by inhibition of LIMK2 with *m*-*calpain* [42]. An up-regulation of LIMK2 likely linked to a down-regulation of miR-1234 in the blood of MFS patients, which was demonstrated in our study, therefore may be related to elevated levels of vascular wall shear stress in the thoracic aorta of MFS patients [43] and be associated with endothelial dysfunction in MFS. Since effective LIMK2 inhibition has already been shown to improve endothelial function in animal models [42]. LIMK2 may represent a promising target for future investigations in MFS patients. Our data shown a significant up-regulation of *guanylate binding protein*-*2* (GBP-2) and a significant inverse correlation with miR-1234 in the blood of MFS patients compared to controls. Human *guanylate binding proteins* (GBPs) are a class of large GTPases which are induced by cytokines like Interferon alpha/gamma, Interleukin-1 and TNF-alpha [44]. GBP-2 has not yet been investigated as comprehensively as GBP-1, but shares 75% sequence identity with this isoform [45] which has been shown to be actively secreted by ECs [46]. Patients with rheumatic diseases like rheumatoid arthritis, systemic lupus erythematosus [22], and systemic sclerosis, which are characterized by a chronic inflammatory vessel activation, show reduced levels of GBP-1 in their peripheral blood [44]. In a rat arteriovenous (AV) loop model, it has been shown that GBP-1 inhibits endothelial cell progenitor migration and leads to endothelial cell dysfunction [44]. An up-regulation of GBP-2 in the blood of MFS patients is likely reflects the vascular pathology and disturbed endothelial cell function in these patients. A significant up-regulation and inverse correlation to miR-1234 in the blood of MFS patients compared to controls was also shown for *dysferlin* (DYSF). Mutations in DYSF lead to limb-girdle muscular dystrophy type 2B and Miyoshi myopathy. DYSF, which is expressed in human ECs, has been shown to form a complex with *platelet endothelial cellular adhesion molecule*-*1* (PECAM-1), thereby preventing its proteosomal degradation [47]. Since PECAM-1 is a ligand of α_V_β_3_-integrin and a promotor of angiogenesis, these data are in line with our observation of an enrichment of gene sets for angiogenesis and integrin signaling in the blood of MFS patients. DYSF is induced in vitro by TNF-alpha and has also been shown to be up-regulated in the blood vessels of patients with multiple sclerosis representing increased vascular inflammation and a disturbed blood–brain barrier [48]. It seems likely that an overexpression of DYSF in the blood of MFS patients, as with GBP-2, represents vascular inflammation in these patients and may be a potential biomarker for the severity of vascular pathologies in MFS warranting further investigations. The gene encoding for *protection of telomeres 1* (POT1) was the only significantly down-regulated gene in the blood of MFS patients (fold-change ≤ 2) compared to controls and exhibited inverse correlations with 7 miRNAs. POT1 binds single-stranded DNA as a heterodimer with *tripeptidyl peptidase 1* (TPP1) and promotes telomerase-mediated telomere extension. Reduced telomere length is recognized as a hallmark of cardiovascular aging and as a biomarker for and TAA and dissections [49, 50]. It has to date not been investigated whether telomere length plays a role in the pathogenesis of MFS. The reduced expression of POT1 in the blood of MFS patients demonstrated in our study, however, indicates, that accelerated cardiac ageing may be present in MFS, which may be reflected in reduced telomere length and POT1 expression. Among the miRNAs inversely correlating with POT1, miR-362 has been linked to the degree of inflammation in samples from abdominal aortic aneurysms [51]. Moreover, miR-500 was shown to be deregulated in degenerative mitral valve disease [52] and miR-502 was also up-regulated in the sera of patients with congestive heart failure [53]. Some genes which we found to be up-regulated in the blood of MFS patients compared to controls play a role in cardiomyocyte differentiation and remodeling during AMI as well as in DCM. Patients with DCM show a strong and lasting increase of *oncostatin M* (OSM) gene expression [23]. Moreover, significant changes in *clusterin* [24] have been detected in patients with AMI [25] and increased levels of *selectin L* (SELL) are associated with ischemic stroke [26]. The JAK/STAT pathway is negatively regulated by the suppressor of cytokine signaling (SOCS) protein, and the myocardium-specific suppressor of *cytokine signaling 3* (SOCS3) gene plays a key role in the development of left ventricular (LV) remodeling after AMI [27]. In agreement with the higher expression level of *C*-*X*-*C motif chemokine ligand 5* (CXCL5) in the blood of patients with MFS, CXCL5 showed an increased expression level in the plasma of patients with coronary artery disease. Recent studies have shown that CXCL5 is up-regulated in abdominal aortic aneurysms (AAA) [31], making it a candidate for potential future anti-inflammatory therapy strategies in MFS. We identified three miRNAs, namely miR-151-5p, miR-324-5p, and miR-500b, which correlated significantly with the Z-score and the LVEDD of MFS patients. MiR-24 and miR-30e correlated only with the LVEDD of MFS patients in our study. MiR-24 has been reported to be up-regulated in tissue from thoracic aortic aneurysms [54] and the miR-30-family was shown to be up-regulated in tissue from thoracic aortic dissections and abdominal aortic aneurysms [55, 56]. Interestingly miR-331-3p, which has been linked to cardiac hypertrophy [57] and miR-200, which also has been linked to cardiovascular disease [58], were down-regulated in MFS patients with MVP compared with patients without MVP. MiR-200c-3p also showed an inverse correlation to POT1 in our study. These deregulated miRNAs may serve as potential future biomarkers in MFS after conformational analysis in studies with larger sample sizes. MiRNA and mRNA profiles measured in PAXgene blood samples comes to a greater extent from the cellular components of the blood, i.e. leukocytes and erythrocytes, and only to a lesser extent from cell-free RNA. Therefore, the expression changes we identified in our study presumably reflect rather changes in the blood cells of the patients rather than expression changes in solid tissue, i.e. bone. One of the MFS clinical manifestations is the musculoskeletal system (typically tall stature with arachnodactyly) and patients with MFS also have significant musculoskeletal phenotypes which may affect the marrow cavity and subsequently influence the hematopoiesis process. Therefore, it is conceivable that changes in miRNA and mRNA expression profile in the blood of MFS patients might be the results of differences in the hematopoiesis process in MFS patients compared to healthy controls. Another line of thinking is, that differences in the mRNA and miRNA profiles might originate from differences in blood flow kinetics between MFS patients and controls. It is known that altered blood pressure has an impact on miRNA expression profile, as shown by Neth et al. [59]. Aortic dilatation and structural cardiac anomalies like MVP in MFS patients exposes the vascular endothelium to altered hemodynamic forces, which may indirectly influence miRNA and mRNA profiles in the blood cells of MFS patients due to different blood flow velocities compared to healthy individuals.

Limitations of our study are related to a relatively small sample size. Moreover, our analysis focused only on the main diagnostic criteria such as FBN1 positivity, aortic root dilatation and lens dislocation and revealed correlations of miRNA expression to cardiovascular features such as aortic root dilatation and mitral valve prolapse. Skeletal features which are characterized by a highly variable age of onset are heterogeneous and have been considered as secondary diagnostic criteria according to the modified Ghent criteria. Certainly, the skeletal features are important leading diagnostic criteria for further evaluation of the patients suspected to have Marfan disease. Correlation of miRNA expression to skeletal abnormalities has to be performed in future studies with larger cohorts of patients with definitive and highly characterized main skeletal features. Future studies also have to investigate whether the observed miRNA expression profiles are specific to MFS or also relate to other syndromes with familial thoracic aortic aneurysm like Loeys–Dietz syndrome, Shprintzen–Goldberg syndrome or mutations in ACTA2.

## Conclusions

We present the first study investigating miRNA and mRNA expression patterns in the peripheral blood of MFS patients in comparison with HV controls. A strong deregulation of both miRNA and mRNA expression profiles was present in MFS patients including multiple genes with high relevance to cardiovascular pathogenesis and diseases. Four genes associated with vascular pathology and inflammation namely MMP9, LIMK2, GBP-2, and DYSF were up-regulated in MFS patients and showed inverse correlations with miR-1234. POT-1 was down-regulated and inversely correlated with 7 miRNAs indicating a potential role of telomere length in the pathogenesis of MFS. These genes represent promising candidates for future investigations aiming at prognostic biomarkers for cardiovascular manifestations in MFS as well as targets for novel therapeutic approaches. Apart from the particular considerations as to the value of the observed distinctive miRNA/mRNA patterns for diagnosis and prognosis of MFS patients, our study fundamentally highlights the extreme breadth of molecular downstream effects initiated by a constitutional single point mutation in a monogenic heritable condition. Pleiotropy also has an as yet underestimated molecular dimension that may provide insights into how complex seemingly “simple” monogenic traits actually are.

## Additional files

**Additional file 1: Figure S1.** Pearson correlation coefficient-based heat map representation between samples. Samples are clustered by the Euclidean distance between rows and columns based on miRNA expression level.

**Additional file 2: Figure S2.** Pearson correlation coefficient-based heat map representation between samples. Samples are clustered by the Euclidean distance between rows and columns based on mRNA expression level.

**Additional file 3: Table S1.** Clinical characteristics of patients.** Table S2.** Significantly expressed transcripts s in the blood of patients with MFS (n = 7) compared HVs controls (n = 7) as determined by microarray (*P-*value <0.05).** Table S3.** Significantly expressed protein coding genes in the blood of patients with MFS (n = 7) compared HVs controls (n = 7) as determined by microarray (*P-*value <0.05).** Table S4.** Over-representation analysis of target genes list.

## Abbreviations

AAA: abdominal aortic aneurysms
AMI: acute myocardial infarction
cDNA: complementary DNA
cRNA: complementary RNA
DCM: dilated cardiomyopathy
EC: endothelial cell
FBN1: Fibrillin-1
GO: Gene Ontology
HV: healthy volunteer
LV: left ventricular
LVEDD: left ventricular end-diastolic diameter
MGS: Marfan syndrome
miRNAs: microRNAs
miRTC: miRNA reverse transcription control
MRI: magnetic resonance imaging
mRNA: messenger RNA
MVP: mitral valve prolapse
NTC: no template control
PANTHER: Protein ANalysis THrough Evolutionary Relationships
PCR: polymerase chain reaction
RT-qPCR: real-time quantitative PCR
snRNA: small nuclear RNA
TAA: thoracic aortic aneurysms
3′UTR: 3′ untranslated region
vsn: variance stabilizing normalization
