# Supplementary material for: Deregulated microRNA and mRNA expression profiles in the peripheral blood of patients with Marfan syndrome
Source: J Transl Med. 2018 Mar 12;16:60. doi: 10.1186/s12967-018-1429-3 (PMC5848586; doi:10.1186/s12967-018-1429-3)
Supplement: Supplementary file 1 — Additional file 1: Figure S1. Pearson correlation coefficient-based heat map representation between samples. Samples are clustered by the Euclidean distance between rows and columns based on miRNA expression level. [file 12967_2018_1429_MOESM1_ESM.pptx]

## Slide 1
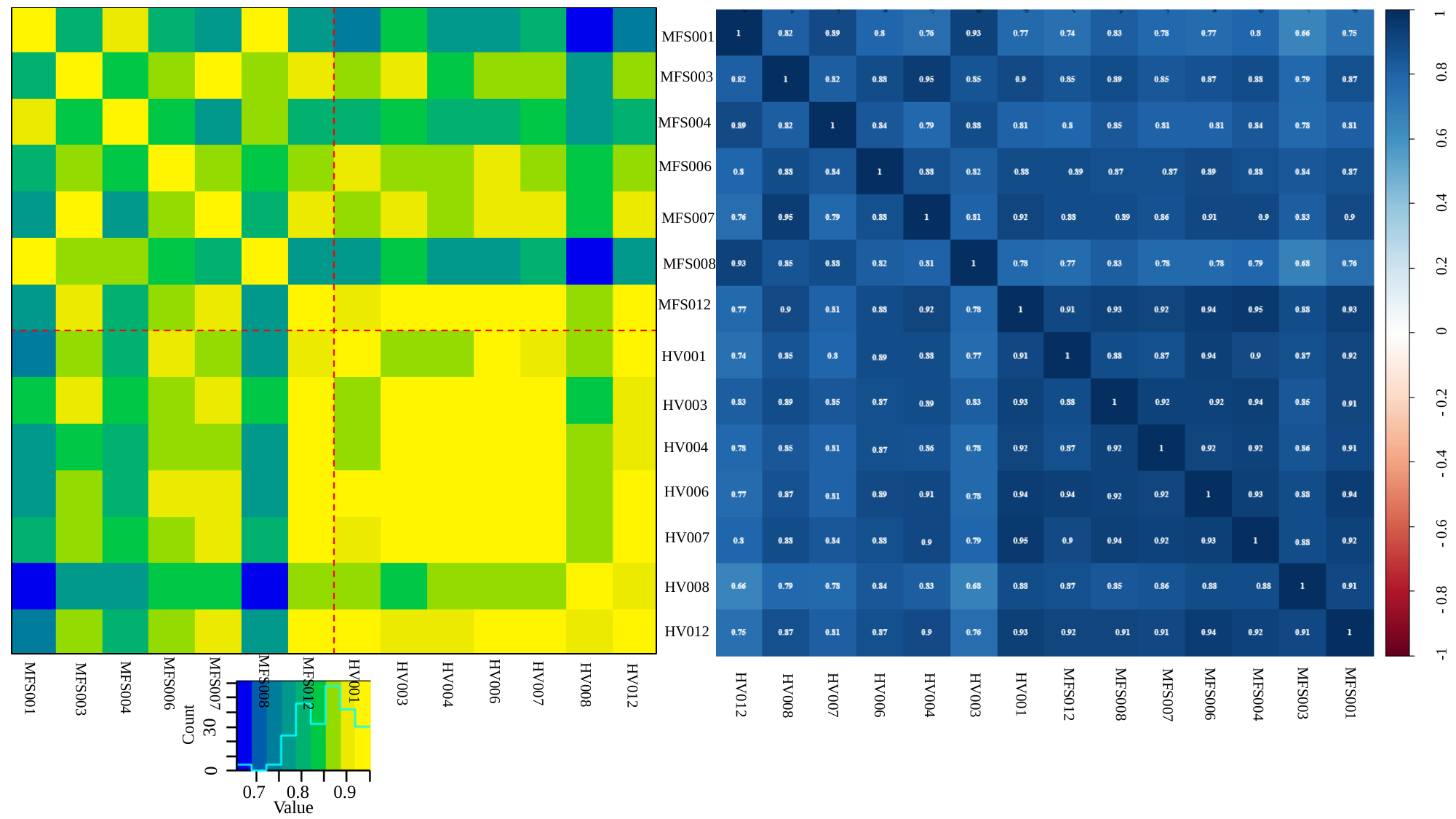

1
MFS001
0.8
MFS003
MFS004
0.6
MFS006
0.4
MFS007
MFS008
0.2
MFS012
0
HV001
- 0.2
HV003
HV004
- 0.4
HV006
- 0.6
HV007
HV008
- 0.8
HV012
-1
HV006
HV001
MFS008
HV003
HV007
MFS012
MFS006
MFS007
HV004
HV008
HV012
MFS004
MFS001
MFS003
MFS003
MFS001
HV001
MFS012
MFS008
MFS006
MFS004
HV012
MFS007
HV007
HV004
HV003
HV006
HV008
Count
30
0
0.7
0.8
0.9
Value
