# Supplementary material for: Deregulated microRNA and mRNA expression profiles in the peripheral blood of patients with Marfan syndrome
Source: J Transl Med. 2018 Mar 12;16:60. doi: 10.1186/s12967-018-1429-3 (PMC5848586; doi:10.1186/s12967-018-1429-3)
Supplement: Supplementary file 2 — Additional file 2: Figure S2. Pearson correlation coefficient-based heat map representation between samples. Samples are clustered by the Euclidean distance between rows and columns based on mRNA expression level. [file 12967_2018_1429_MOESM2_ESM.pptx]

## Slide 1
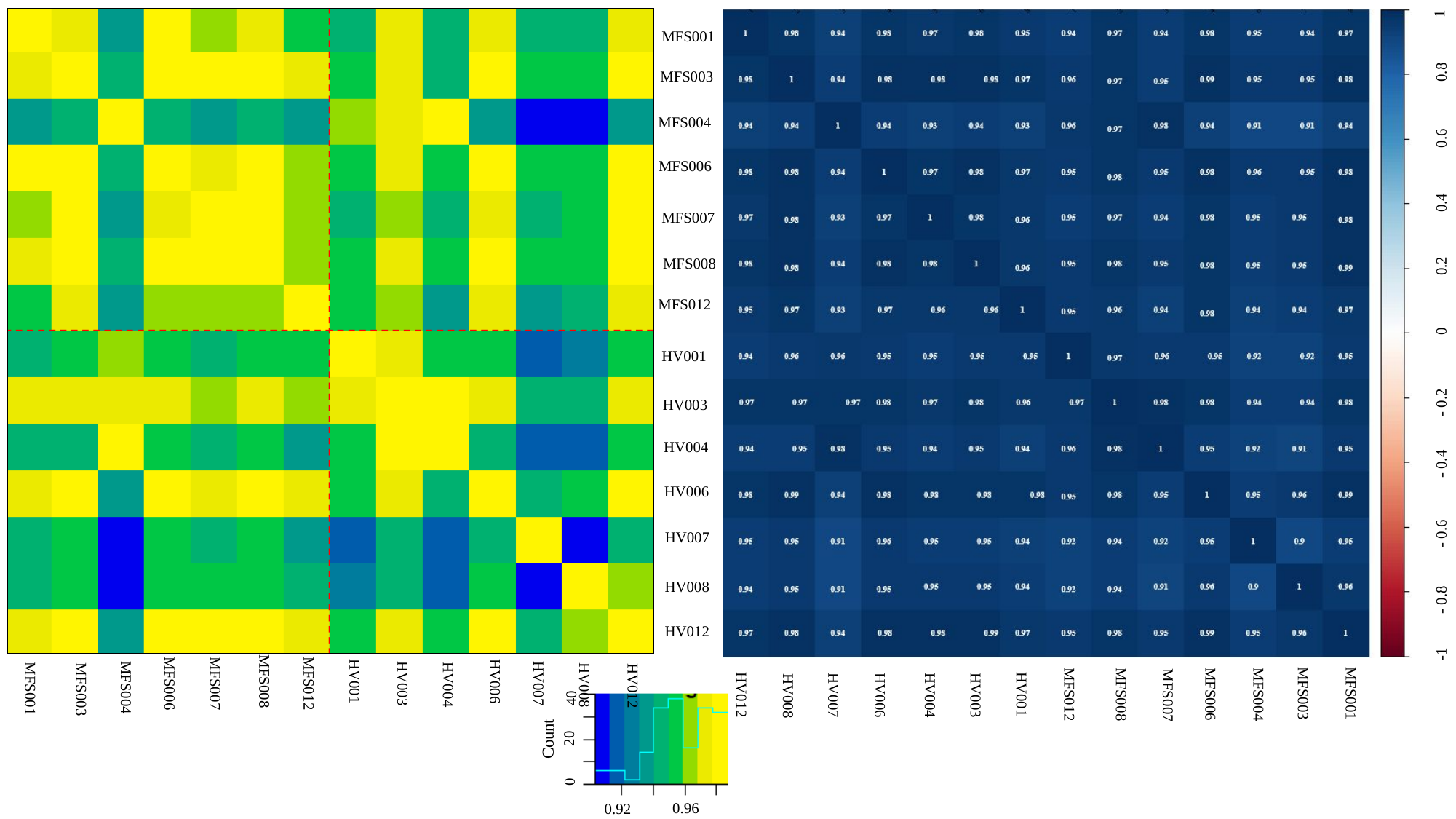

1
MFS001
0.8
MFS003
MFS004
0.6
MFS006
0.4
MFS007
MFS008
0.2
MFS012
0
HV001
- 0.2
HV003
HV004
- 0.4
HV006
- 0.6
HV007
HV008
- 0.8
HV012
-1
HV006
HV001
MFS008
HV003
HV007
MFS012
MFS006
MFS007
HV004
HV008
HV012
MFS004
MFS001
MFS003
MFS003
MFS001
HV001
MFS012
MFS008
MFS006
MFS004
HV012
MFS007
HV007
HV004
HV003
HV006
HV008
40
Count
20
0
0.96
0.92
