# Supplementary material for: Deregulated microRNA and mRNA expression profiles in the peripheral blood of patients with Marfan syndrome
Source: J Transl Med. 2018 Mar 12;16:60. doi: 10.1186/s12967-018-1429-3 (PMC5848586; doi:10.1186/s12967-018-1429-3)
Supplement: Supplementary file 3 — Additional file 3: Table S1. Clinical characteristics of patients. Table S2. Significantly expressed transcripts s in the blood of patients with MFS (n = 7) compared HVs controls (n = 7) as determined by microarray (P-value <0.05). Table S3. Significantly expressed protein coding genes in the blood of patients with MFS (n = 7) compared HVs controls (n = 7) as determined by microarray (P-value <0.05). Table S4. Over-representation analysis of target genes list. [file 12967_2018_1429_MOESM3_ESM.docx]

**Table S1:** Clinical characteristics of patients.

| **Sample** | **Age** | **Sex** | **FBN1 Mutation** | **Mitral valve prolapse** | **Ectopia lentis** | **Aortic root status (Z score)** | **LVEDD*** |
| --- | --- | --- | --- | --- | --- | --- | --- |
| Microarray screening ( n=8) | | | | | | | |
| MFS 001 | 19 | Male | Yes | Yes | No | 5 | 61 |
| MFS 003 | 16 | Male | Yes | Yes | No | 1.23 | 42 |
| MFS 004 | 50 | Female | Yes | Yes | No | 5 | 42 |
| MFS 006 | 19 | Female | Yes | No | No | 1.66 | 47 |
| MFS 007 | 14 | Female | Yes | Yes | No | 5 | 41 |
| MFS 008 | 41 | Female | Yes | No | No | 3.3 | 48 |
| MFS 011* | 20 | Female | Yes | Yes | No | 5 | 59 |
| MFS 012 | 61 | Female | Yes | Yes | No | 5 | 48 |
|  | | | | | | | |
| **RT-qPCR validation (n=26)** | | | | | | | |
| MFS 002 | 52 | Male | Yes | Yes | No | 5 | 60 |
| MFS 005 | 54 | Male | Yes | Yes | No | 5 | 55 |
| MFS 009 | 37 | Female | Yes | Yes | Yes | 5 | 51 |
| MFS 010 | 10 | Female | Yes | Yes | Yes | 4.6 | 42 |
| MFS 013 | 62 | Male | Yes | Yes | No | 5 | 55 |
| MFS 014 | 7 | Male | Yes | Yes | No | 3.4 | 37 |
| MFS 015 | 20 | Male | Yes | No | No | 2.83 | 51 |
| MFS 016 | 26 | Male | Yes | Yes | No | 3.75 | 45 |
| MFS 017 | 11 | Female | Yes | Yes | Yes | 6.6 | 39 |
| MFS 018 | 15 | Male | Yes | Yes | No | 6.2 | 50 |
| MFS 019 | 38 | Female | Yes | Yes | Yes | 5 | 53 |
| MFS 020 | 35 | Male | Yes | Yes | No | 5 | 55 |
| MFS 021 | 22 | Female | Yes | Yes | No | 2.89 | 54 |
| MFS 022 | 22 | Female | Yes | Yes | No | 4.21 | 46 |
| MFS 023 | 39 | Male | Yes | No | No | 5 | 55 |
| MFS 024 | 42 | Female | Yes | No | No | 5 | 58 |
| MFS 025 | 19 | Female | Yes | Yes | No | 3.41 | 58 |
| MFS 026 | 40 | Male | Yes | No | No | 5 | 60 |
| MFS 027 | 6 | Male | Yes | Yes | No | 5.4 | 44 |
| MFS 028 | 6 | Female | Yes | No | Yes | 3.3 | 38 |
| MFS 029 | 31 | Female | Yes | No | No | 4.35 | 44 |
| MFS 030 | 20 | Male | Yes | Yes | No | 4.05 | 62 |
| MFS 031 | 20 | Female | Yes | No | No | 1.12 | 50 |
| MFS 032 | 25 | Male | Yes | No | Yes | 3.36 | 52 |
| MFS 033 | 20 | Female | Yes | No | No | 0.66 | 49 |
| MFS 034 | 20 | Female | Yes | No | No | 3.45 | 56 |

* Sample was excluded from analysis due to detection metrics failure. Abbreviations: ARR. aortic root replacement by aortic dissection or aortic aneurysm (Aortic root >52 mm); LVEDD, left ventricular end-diastolic diameter.

**Table S2:** Significantly deregulated mRNA transcripts in the serum of patients with Marfan Syndrome (MFS) (n = 7) compared to healthy volunteer (HV) controls (n = 7) as determined by microarray analysis (P-values <0.05). AUC = area under the receiver operating characteristics curve.

| **Transcript Name** | **Systematic Name** | **Median MFS** | **Median HVs** | **Fold Change** | **Log2 Fold Change** | **Regulation** | **P-value** | **AUC** |
| --- | --- | --- | --- | --- | --- | --- | --- | --- |
| POT1 | NM_015450 | 248.67 | 530.62 | 0.47 | -1.09 | Down | 0.000168 | 1.00 |
| LOC100506476 | XR_110221 | 69.43 | 137.21 | 0.51 | -0.98 | Down | 0.019123 | 0.84 |
| C17orf97 | NM_001013672 | 61.69 | 112.85 | 0.55 | -0.87 | Down | 0.026108 | 0.80 |
| LOC100131564 | NR_034089 | 173.56 | 284.11 | 0.61 | -0.71 | Down | 0.020267 | 0.88 |
| LOC100507100 | XR_108691 | 218.02 | 355.18 | 0.61 | -0.70 | Down | 0.008353 | 0.90 |
| FCER1A | NM_002001 | 560.85 | 908.25 | 0.62 | -0.70 | Down | 0.023762 | 0.88 |
| XLOC_009337 | TCONS_00019542 | 70.14 | 113.13 | 0.62 | -0.69 | Down | 0.041597 | 0.78 |
| XLOC_005471 | ENST00000434255 | 55.72 | 89.16 | 0.62 | -0.68 | Down | 0.048095 | 0.76 |
| ANKRD20A5P | NR_040113 | 94.44 | 151.05 | 0.63 | -0.68 | Down | 0.017936 | 0.88 |
| XLOC_001020 | TCONS_00001678 | 165.89 | 263.37 | 0.63 | -0.67 | Down | 0.010859 | 0.90 |
| ARHGEF15 | NM_173728 | 287.81 | 453.65 | 0.63 | -0.66 | Down | 0.042069 | 0.80 |
| A_33_P3365501 | A_33_P3365501 | 183.37 | 281.36 | 0.65 | -0.62 | Down | 0.011254 | 0.92 |
| DA734158 | DA734158 | 104.66 | 157.71 | 0.66 | -0.59 | Down | 0.015890 | 0.92 |
| ME3 | NM_001014811 | 87.20 | 130.90 | 0.67 | -0.59 | Down | 0.039721 | 0.80 |
| XLOC_l2_011170 | TCONS_l2_00021187 | 36.07 | 54.12 | 0.67 | -0.59 | Down | 0.049867 | 0.76 |
| LOC728431 | NR_038842 | 464.80 | 310.82 | 1.50 | 0.58 | Up | 0.002011 | 0.08 |
| RPS2 | NM_002952 | 177799.76 | 118882.72 | 1.50 | 0.58 | Up | 0.034702 | 0.16 |
| ACOX1 | NM_001185039 | 1964.04 | 1313.10 | 1.50 | 0.58 | Up | 0.021373 | 0.14 |
| CRISPLD2 | NM_031476 | 172.58 | 115.38 | 1.50 | 0.58 | Up | 0.025060 | 0.16 |
| NUB1 | NM_016118 | 929.94 | 621.64 | 1.50 | 0.58 | Up | 0.018410 | 0.14 |
| XLOC_006069 | ENST00000450016 | 194.90 | 130.19 | 1.50 | 0.58 | Up | 0.027421 | 0.14 |
| CD97 | NM_078481 | 17074.58 | 11399.16 | 1.50 | 0.58 | Up | 0.045424 | 0.14 |
| XLOC_l2_005557 | TCONS_l2_00010291 | 111212.90 | 74240.23 | 1.50 | 0.58 | Up | 0.027779 | 0.14 |
| MYLK | NM_053025 | 152.00 | 101.43 | 1.50 | 0.58 | Up | 0.017457 | 0.08 |
| GPSM3 | NM_022107 | 8219.77 | 5484.36 | 1.50 | 0.58 | Up | 0.030723 | 0.16 |
| JAK3 | NM_000215 | 11611.30 | 7744.18 | 1.50 | 0.58 | Up | 0.038115 | 0.20 |
| REEP4 | NM_025232 | 369.19 | 245.94 | 1.50 | 0.59 | Up | 0.023934 | 0.16 |
| HLA-DRB1 | NM_002124 | 17676.69 | 11768.64 | 1.50 | 0.59 | Up | 0.016528 | 0.14 |
| ACTB | NM_001101 | 148311.47 | 98698.04 | 1.50 | 0.59 | Up | 0.001192 | 0.06 |
| PMPCA | NM_015160 | 642.31 | 427.34 | 1.50 | 0.59 | Up | 0.044147 | 0.22 |
| AATF | NM_012138 | 1135.29 | 755.31 | 1.50 | 0.59 | Up | 0.017090 | 0.16 |
| XRCC3 | ENST00000557439 | 43839.49 | 29158.30 | 1.50 | 0.59 | Up | 0.035969 | 0.18 |
| RFX1 | NM_002918 | 1416.30 | 941.94 | 1.50 | 0.59 | Up | 0.047826 | 0.18 |
| UBA1 | NM_003334 | 1140.49 | 758.49 | 1.50 | 0.59 | Up | 0.025650 | 0.12 |
| SRC | NM_005417 | 158.47 | 105.37 | 1.50 | 0.59 | Up | 0.008616 | 0.10 |
| EHD1 | NM_006795 | 9247.99 | 6148.43 | 1.50 | 0.59 | Up | 0.017749 | 0.14 |
| ITPRIP | NM_033397 | 4827.55 | 3206.52 | 1.51 | 0.59 | Up | 0.007497 | 0.12 |
| LIMK1 | NM_002314 | 347.82 | 230.63 | 1.51 | 0.59 | Up | 0.009530 | 0.12 |
| TNRC18 | NM_001080495 | 1425.65 | 945.26 | 1.51 | 0.59 | Up | 0.014682 | 0.10 |
| BBX | NM_020235 | 1404.32 | 930.66 | 1.51 | 0.59 | Up | 0.041901 | 0.20 |
| REC8 | NM_001048205 | 1558.93 | 1032.06 | 1.51 | 0.60 | Up | 0.036326 | 0.20 |
| A_33_P3304769 | A_33_P3304769 | 92.85 | 61.47 | 1.51 | 0.60 | Up | 0.004527 | 0.08 |
| ENST00000506864 | ENST00000506864 | 110.58 | 73.19 | 1.51 | 0.60 | Up | 0.033681 | 0.14 |
| FRAT2 | NM_012083 | 14460.43 | 9570.51 | 1.51 | 0.60 | Up | 0.023387 | 0.14 |
| LOC100133161 | NR_028326 | 65051.55 | 43047.95 | 1.51 | 0.60 | Up | 0.047408 | 0.18 |
| ZNF641 | NM_152320 | 456.99 | 302.28 | 1.51 | 0.60 | Up | 0.028318 | 0.10 |
| AFF1 | NM_005935 | 241.07 | 159.34 | 1.51 | 0.60 | Up | 0.032863 | 0.14 |
| PSAP | NM_001042465 | 13757.98 | 9087.50 | 1.51 | 0.60 | Up | 0.033009 | 0.16 |
| A_33_P3336038 | A_33_P3336038 | 33449.05 | 22091.25 | 1.51 | 0.60 | Up | 0.020554 | 0.12 |
| PLIN3 | NM_005817 | 455.49 | 300.66 | 1.51 | 0.60 | Up | 0.041854 | 0.16 |
| MX2 | ENST00000495892 | 305.95 | 201.74 | 1.52 | 0.60 | Up | 0.004611 | 0.10 |
| LINC00265 | NR_026999 | 11472.99 | 7564.09 | 1.52 | 0.60 | Up | 0.030358 | 0.16 |
| SMARCD3 | NM_003078 | 1593.17 | 1050.29 | 1.52 | 0.60 | Up | 0.012551 | 0.16 |
| XLOC_004229 | TCONS_00008716 | 55.47 | 36.56 | 1.52 | 0.60 | Up | 0.022956 | 0.20 |
| CSNK1G2 | NM_001319 | 16865.90 | 11109.46 | 1.52 | 0.60 | Up | 0.036516 | 0.12 |
| WASF3 | NM_006646 | 48.88 | 32.19 | 1.52 | 0.60 | Up | 0.032010 | 0.18 |
| GUK1 | NM_000858 | 19244.34 | 12668.08 | 1.52 | 0.60 | Up | 0.035033 | 0.18 |
| NT5M | NM_020201 | 1539.60 | 1013.31 | 1.52 | 0.60 | Up | 0.033526 | 0.18 |
| YY1AP1 | NM_139118 | 2695.66 | 1773.74 | 1.52 | 0.60 | Up | 0.019937 | 0.14 |
| LCP1 | NM_002298 | 42127.01 | 27719.38 | 1.52 | 0.60 | Up | 0.005107 | 0.08 |
| LRRN2 | NM_201630 | 191.20 | 125.79 | 1.52 | 0.60 | Up | 0.028352 | 0.14 |
| BRD3 | NM_007371 | 415.67 | 273.40 | 1.52 | 0.60 | Up | 0.033932 | 0.18 |
| MOB3A | NM_130807 | 5957.92 | 3918.30 | 1.52 | 0.60 | Up | 0.022703 | 0.14 |
| VASP | NM_003370 | 11722.83 | 7709.49 | 1.52 | 0.60 | Up | 0.005976 | 0.06 |
| IQGAP1 | NM_003870 | 10967.14 | 7210.20 | 1.52 | 0.61 | Up | 0.001416 | 0.02 |
| GHRLOS | NR_004431 | 531.83 | 349.53 | 1.52 | 0.61 | Up | 0.022454 | 0.18 |
| RGS3 | NM_134427 | 1234.24 | 810.65 | 1.52 | 0.61 | Up | 0.005417 | 0.10 |
| SKIV2L | NM_006929 | 251.30 | 164.91 | 1.52 | 0.61 | Up | 0.043080 | 0.20 |
| IRF9 | NM_006084 | 3741.04 | 2454.71 | 1.52 | 0.61 | Up | 0.005976 | 0.10 |
| XLOC_l2_012953 | TCONS_l2_00024711 | 121079.92 | 79434.31 | 1.52 | 0.61 | Up | 0.029186 | 0.16 |
| LAMA5 | ENST00000370677 | 60599.60 | 39740.69 | 1.52 | 0.61 | Up | 0.031824 | 0.14 |
| FLJ40606 | BC133006 | 89070.44 | 58392.26 | 1.53 | 0.61 | Up | 0.048127 | 0.20 |
| PSTPIP1 | NM_003978 | 5070.96 | 3324.06 | 1.53 | 0.61 | Up | 0.023325 | 0.14 |
| ARHGDIB | NM_001175 | 87318.28 | 57212.59 | 1.53 | 0.61 | Up | 0.012694 | 0.14 |
| PSMD2 | NM_002808 | 8033.14 | 5262.15 | 1.53 | 0.61 | Up | 0.049442 | 0.20 |
| BBX | NM_020235 | 1401.21 | 917.75 | 1.53 | 0.61 | Up | 0.018058 | 0.12 |
| IFITM2 | NM_006435 | 125829.65 | 82394.77 | 1.53 | 0.61 | Up | 0.049568 | 0.18 |
| CRAT | NM_000755 | 2292.20 | 1500.92 | 1.53 | 0.61 | Up | 0.016242 | 0.14 |
| CD300E | NM_181449 | 1636.57 | 1071.22 | 1.53 | 0.61 | Up | 0.004539 | 0.10 |
| TFE3 | NM_006521 | 628.17 | 411.15 | 1.53 | 0.61 | Up | 0.013533 | 0.12 |
| FHOD1 | NM_013241 | 590.39 | 386.16 | 1.53 | 0.61 | Up | 0.043994 | 0.22 |
| TRAFD1 | NM_001143906 | 4739.25 | 3099.57 | 1.53 | 0.61 | Up | 0.027516 | 0.18 |
| LOC388210 | XM_001716361 | 204.16 | 133.52 | 1.53 | 0.61 | Up | 0.014666 | 0.12 |
| PTPRC | NM_002838 | 39023.82 | 25517.06 | 1.53 | 0.61 | Up | 0.025571 | 0.14 |
| WDFY4 | NM_020945 | 4574.22 | 2988.80 | 1.53 | 0.61 | Up | 0.020598 | 0.16 |
| LOC100507948 | XR_112445 | 1330.67 | 869.42 | 1.53 | 0.61 | Up | 0.030360 | 0.16 |
| TLN1 | NM_006289 | 658.02 | 429.62 | 1.53 | 0.62 | Up | 0.001808 | 0.06 |
| XLOC_004924 | TCONS_00010402 | 1281.73 | 836.54 | 1.53 | 0.62 | Up | 0.032417 | 0.20 |
| MEFV | NM_000243 | 627.29 | 408.90 | 1.53 | 0.62 | Up | 0.016228 | 0.18 |
| RALBP1 | NM_006788 | 909.87 | 593.08 | 1.53 | 0.62 | Up | 0.003566 | 0.08 |
| RALB | NM_002881 | 3573.45 | 2327.50 | 1.54 | 0.62 | Up | 0.034330 | 0.16 |
| ODF3B | NM_001014440 | 1452.92 | 946.02 | 1.54 | 0.62 | Up | 0.023947 | 0.16 |
| SIRPB1 | NM_006065 | 363.53 | 236.56 | 1.54 | 0.62 | Up | 0.042897 | 0.20 |
| SLC43A2 | NM_152346 | 2517.20 | 1637.77 | 1.54 | 0.62 | Up | 0.019643 | 0.14 |
| NTNG2 | NM_032536 | 2073.58 | 1348.56 | 1.54 | 0.62 | Up | 0.036137 | 0.12 |
| LOC100134663 | XM_001718823 | 617.37 | 401.30 | 1.54 | 0.62 | Up | 0.006320 | 0.12 |
| PDGFA | NM_002607 | 74.03 | 48.10 | 1.54 | 0.62 | Up | 0.010320 | 0.14 |
| XLOC_004924 | BQ232757 | 1066.19 | 692.73 | 1.54 | 0.62 | Up | 0.002341 | 0.06 |
| FTH1 | NM_002032 | 68397.05 | 44424.91 | 1.54 | 0.62 | Up | 0.001582 | 0.06 |
| ENST00000381105 | ENST00000381105 | 96345.33 | 62575.74 | 1.54 | 0.62 | Up | 0.003517 | 0.06 |
| F8A1 | NM_012151 | 5912.91 | 3838.26 | 1.54 | 0.62 | Up | 0.031958 | 0.16 |
| XLOC_013994 | HQ013231 | 3645.41 | 2365.80 | 1.54 | 0.62 | Up | 0.013037 | 0.10 |
| XLOC_006132 | DB461521 | 590.03 | 382.73 | 1.54 | 0.62 | Up | 0.020674 | 0.14 |
| MBD6 | NM_052897 | 1716.34 | 1112.72 | 1.54 | 0.63 | Up | 0.009399 | 0.16 |
| WDR81 | NM_152348 | 1331.60 | 863.15 | 1.54 | 0.63 | Up | 0.033990 | 0.16 |
| CAMK1D | NM_020397 | 691.41 | 447.97 | 1.54 | 0.63 | Up | 0.045805 | 0.20 |
| RBPJ | NM_203284 | 2278.12 | 1475.22 | 1.54 | 0.63 | Up | 0.039188 | 0.20 |
| PKM2 | NM_182470 | 4246.63 | 2748.08 | 1.55 | 0.63 | Up | 0.009030 | 0.10 |
| CARS2 | NM_024537 | 13627.39 | 8816.83 | 1.55 | 0.63 | Up | 0.010369 | 0.12 |
| NEXN | NM_144573 | 196.54 | 127.12 | 1.55 | 0.63 | Up | 0.033888 | 0.22 |
| AY927536 | AY927536 | 1271.77 | 822.23 | 1.55 | 0.63 | Up | 0.027967 | 0.16 |
| F8A1 | NM_012151 | 5814.21 | 3757.74 | 1.55 | 0.63 | Up | 0.035973 | 0.18 |
| GIT2 | NM_057169 | 2261.20 | 1461.16 | 1.55 | 0.63 | Up | 0.048802 | 0.16 |
| CS | NM_004077 | 1408.67 | 909.95 | 1.55 | 0.63 | Up | 0.030505 | 0.16 |
| NFKB1 | NM_003998 | 1650.55 | 1065.96 | 1.55 | 0.63 | Up | 0.039449 | 0.16 |
| RNF19B | NM_153341 | 505.20 | 326.17 | 1.55 | 0.63 | Up | 0.011824 | 0.10 |
| IL6R | NM_000565 | 1315.87 | 849.46 | 1.55 | 0.63 | Up | 0.009036 | 0.10 |
| ATP6V0D1 | NM_004691 | 7935.11 | 5122.32 | 1.55 | 0.63 | Up | 0.040365 | 0.18 |
| PLIN3 | NM_005817 | 506.77 | 326.87 | 1.55 | 0.63 | Up | 0.009764 | 0.12 |
| LOC145474 | NR_027046 | 433.18 | 279.17 | 1.55 | 0.63 | Up | 0.006787 | 0.10 |
| A_33_P3312754 | A_33_P3312754 | 478.75 | 308.25 | 1.55 | 0.64 | Up | 0.043017 | 0.20 |
| PNKP | NM_007254 | 622.30 | 400.47 | 1.55 | 0.64 | Up | 0.038045 | 0.18 |
| PRR12 | NM_020719 | 394.87 | 254.03 | 1.55 | 0.64 | Up | 0.041370 | 0.22 |
| F8A1 | NM_012151 | 6489.27 | 4173.92 | 1.55 | 0.64 | Up | 0.030959 | 0.16 |
| HLA-J | NR_024240 | 103949.96 | 66851.34 | 1.55 | 0.64 | Up | 0.026970 | 0.16 |
| NBEAL2 | NM_015175 | 1990.02 | 1278.98 | 1.56 | 0.64 | Up | 0.031576 | 0.18 |
| TRIM56 | NM_030961 | 1563.02 | 1003.52 | 1.56 | 0.64 | Up | 0.032607 | 0.16 |
| SND1 | NM_014390 | 2948.64 | 1893.10 | 1.56 | 0.64 | Up | 0.019327 | 0.12 |
| INA | NM_032727 | 73.96 | 47.46 | 1.56 | 0.64 | Up | 0.026365 | 0.18 |
| LOC100506190 | BC038559 | 634.52 | 407.02 | 1.56 | 0.64 | Up | 0.045080 | 0.16 |
| CTSZ | NM_001336 | 1178.60 | 755.17 | 1.56 | 0.64 | Up | 0.011669 | 0.04 |
| LOC93444 | AK000175 | 165.37 | 105.92 | 1.56 | 0.64 | Up | 0.039363 | 0.18 |
| XLOC_005355 | THC2673986 | 247.67 | 158.60 | 1.56 | 0.64 | Up | 0.034368 | 0.10 |
| SPATA13 | NM_153023 | 1441.65 | 923.04 | 1.56 | 0.64 | Up | 0.008671 | 0.08 |
| NLRP3 | NM_001079821 | 833.69 | 533.30 | 1.56 | 0.64 | Up | 0.045272 | 0.18 |
| HLA-DRB1 | NM_002124 | 15806.19 | 10107.67 | 1.56 | 0.65 | Up | 0.027030 | 0.14 |
| SASH3 | NM_018990 | 4146.40 | 2650.76 | 1.56 | 0.65 | Up | 0.013822 | 0.14 |
| LYN | NM_002350 | 22722.02 | 14522.33 | 1.56 | 0.65 | Up | 0.012085 | 0.12 |
| FOXH1 | NM_003923 | 825.37 | 527.28 | 1.57 | 0.65 | Up | 0.049010 | 0.14 |
| NCOR2 | NM_006312 | 4137.39 | 2640.72 | 1.57 | 0.65 | Up | 0.029980 | 0.12 |
| LOC728431 | NR_038842 | 478.98 | 305.54 | 1.57 | 0.65 | Up | 0.002185 | 0.06 |
| AMICA1 | NM_153206 | 55523.07 | 35401.63 | 1.57 | 0.65 | Up | 0.017244 | 0.16 |
| ICAM1 | NM_000201 | 340.44 | 217.06 | 1.57 | 0.65 | Up | 0.031902 | 0.14 |
| HLA-DRB1 | NM_002124 | 18264.37 | 11637.71 | 1.57 | 0.65 | Up | 0.028344 | 0.14 |
| TNFSF10 | NM_003810 | 4438.05 | 2827.05 | 1.57 | 0.65 | Up | 0.046020 | 0.20 |
| SLC45A4 | NM_001080431 | 673.23 | 428.83 | 1.57 | 0.65 | Up | 0.041845 | 0.20 |
| LCE1D | NM_178352 | 39163.82 | 24919.94 | 1.57 | 0.65 | Up | 0.021693 | 0.14 |
| XLOC_l2_000727 | TCONS_l2_00000966 | 864.69 | 550.07 | 1.57 | 0.65 | Up | 0.021575 | 0.12 |
| ADAR | NM_001111 | 10454.67 | 6649.27 | 1.57 | 0.65 | Up | 0.016561 | 0.14 |
| RN28S1 | NR_003287 | 215828.30 | 137232.25 | 1.57 | 0.65 | Up | 0.017947 | 0.12 |
| LOC100653058 | XM_003403391 | 498.48 | 316.95 | 1.57 | 0.65 | Up | 0.035265 | 0.18 |
| MBOAT7 | NM_024298 | 10938.91 | 6953.37 | 1.57 | 0.65 | Up | 0.024181 | 0.16 |
| PLIN3 | NM_005817 | 506.62 | 321.59 | 1.58 | 0.66 | Up | 0.028174 | 0.18 |
| TLR4 | NM_138554 | 1160.82 | 736.69 | 1.58 | 0.66 | Up | 0.018655 | 0.12 |
| HBB | NM_000518 | 254838.20 | 161705.39 | 1.58 | 0.66 | Up | 0.024888 | 0.10 |
| INSL3 | NM_005543 | 785.59 | 498.42 | 1.58 | 0.66 | Up | 0.013141 | 0.12 |
| CRISPLD2 | NM_031476 | 165.44 | 104.80 | 1.58 | 0.66 | Up | 0.031354 | 0.16 |
| THRAP3 | NM_005119 | 5526.64 | 3500.71 | 1.58 | 0.66 | Up | 0.030155 | 0.18 |
| SLED1 | NR_003542 | 276.12 | 174.80 | 1.58 | 0.66 | Up | 0.019330 | 0.12 |
| NAIP | NM_004536 | 226.69 | 143.47 | 1.58 | 0.66 | Up | 0.048446 | 0.20 |
| DNAJC5 | NM_025219 | 1284.93 | 811.49 | 1.58 | 0.66 | Up | 0.034610 | 0.16 |
| SLC16A3 | NM_001042422 | 18272.33 | 11527.80 | 1.59 | 0.66 | Up | 0.010773 | 0.14 |
| KPNB1 | NM_002265 | 9381.01 | 5914.30 | 1.59 | 0.67 | Up | 0.033156 | 0.18 |
| XLOC_006593 | ENST00000454957 | 97.73 | 61.61 | 1.59 | 0.67 | Up | 0.042255 | 0.20 |
| ACSS2 | NM_018677 | 496.80 | 312.87 | 1.59 | 0.67 | Up | 0.038412 | 0.24 |
| ITGAM | NM_000632 | 1599.19 | 1007.05 | 1.59 | 0.67 | Up | 0.043946 | 0.20 |
| TCIRG1 | NM_006019 | 8455.15 | 5324.26 | 1.59 | 0.67 | Up | 0.017393 | 0.16 |
| MXD1 | NM_002357 | 5507.07 | 3467.13 | 1.59 | 0.67 | Up | 0.006359 | 0.08 |
| CAMK1D | NM_153498 | 2825.02 | 1778.24 | 1.59 | 0.67 | Up | 0.025298 | 0.14 |
| FAM129B | NM_022833 | 1564.70 | 984.18 | 1.59 | 0.67 | Up | 0.044314 | 0.20 |
| ACTN1 | NM_001102 | 3339.62 | 2100.42 | 1.59 | 0.67 | Up | 0.017378 | 0.08 |
| PARP14 | NM_017554 | 2354.24 | 1479.44 | 1.59 | 0.67 | Up | 0.023638 | 0.10 |
| HOMER3 | NM_001145722 | 4250.05 | 2670.18 | 1.59 | 0.67 | Up | 0.042852 | 0.20 |
| C7orf74 | NM_175884 | 863.76 | 542.33 | 1.59 | 0.67 | Up | 0.021296 | 0.10 |
| ACSL1 | NM_001995 | 3984.27 | 2500.30 | 1.59 | 0.67 | Up | 0.049141 | 0.16 |
| PRR24 | NM_178511 | 936.76 | 587.62 | 1.59 | 0.67 | Up | 0.049433 | 0.22 |
| NARF | NM_001038618 | 2619.05 | 1642.27 | 1.59 | 0.67 | Up | 0.006142 | 0.08 |
| ZFP106 | NM_022473 | 1986.06 | 1244.64 | 1.60 | 0.67 | Up | 0.004114 | 0.08 |
| ITGAM | NM_000632 | 1271.75 | 796.68 | 1.60 | 0.67 | Up | 0.048235 | 0.18 |
| XPO6 | NM_015171 | 2640.65 | 1652.89 | 1.60 | 0.68 | Up | 0.024833 | 0.18 |
| ICAM1 | NM_000201 | 359.71 | 225.15 | 1.60 | 0.68 | Up | 0.038235 | 0.18 |
| HBB | NM_000518 | 256019.67 | 159999.60 | 1.60 | 0.68 | Up | 0.009953 | 0.10 |
| XLOC_002473 | TCONS_00004564 | 164.52 | 102.80 | 1.60 | 0.68 | Up | 0.047676 | 0.20 |
| PLIN3 | NM_005817 | 521.13 | 325.53 | 1.60 | 0.68 | Up | 0.008702 | 0.10 |
| KAT6A | NM_001099412 | 2362.09 | 1475.31 | 1.60 | 0.68 | Up | 0.028333 | 0.16 |
| ATF6 | NM_007348 | 923.33 | 576.22 | 1.60 | 0.68 | Up | 0.009355 | 0.10 |
| ZNF333 | NM_032433 | 422.64 | 263.51 | 1.60 | 0.68 | Up | 0.026973 | 0.12 |
| WWC3 | NM_015691 | 1328.68 | 828.14 | 1.60 | 0.68 | Up | 0.004092 | 0.10 |
| HIF3A | ENST00000457865 | 1362.04 | 848.32 | 1.61 | 0.68 | Up | 0.002277 | 0.10 |
| KCNQ3 | NM_004519 | 1639.06 | 1019.87 | 1.61 | 0.68 | Up | 0.041976 | 0.18 |
| CYFIP1 | NM_014608 | 350.24 | 217.92 | 1.61 | 0.68 | Up | 0.020483 | 0.12 |
| XLOC_l2_000720 | ENST00000424587 | 421.03 | 261.93 | 1.61 | 0.68 | Up | 0.025875 | 0.20 |
| PF4 | NM_002619 | 11528.73 | 7171.72 | 1.61 | 0.68 | Up | 0.031326 | 0.18 |
| LOC100507006 | XR_109941 | 428.16 | 266.11 | 1.61 | 0.69 | Up | 0.020244 | 0.12 |
| STAT1 | NM_139266 | 2695.71 | 1673.11 | 1.61 | 0.69 | Up | 0.032803 | 0.16 |
| XLOC_l2_015885 | TCONS_l2_00030893 | 62000.95 | 38477.29 | 1.61 | 0.69 | Up | 0.014677 | 0.10 |
| XLOC_009192 | AK091141 | 1647.70 | 1021.24 | 1.61 | 0.69 | Up | 0.040286 | 0.16 |
| CMA1 | NM_001836 | 111.05 | 68.80 | 1.61 | 0.69 | Up | 0.049348 | 0.20 |
| LOC100170939 | NR_024054 | 1638.82 | 1014.74 | 1.62 | 0.69 | Up | 0.040999 | 0.16 |
| NT5C3 | NM_001002010 | 2632.41 | 1629.66 | 1.62 | 0.69 | Up | 0.002358 | 0.04 |
| ATP1A1 | NM_000701 | 1913.19 | 1184.16 | 1.62 | 0.69 | Up | 0.035995 | 0.16 |
| XLOC_002049 | ENST00000418746 | 280.85 | 173.78 | 1.62 | 0.69 | Up | 0.043145 | 0.12 |
| HLA-DRB1 | NM_002124 | 19355.39 | 11970.25 | 1.62 | 0.69 | Up | 0.034659 | 0.18 |
| AQP1 | NM_198098 | 317.43 | 196.26 | 1.62 | 0.69 | Up | 0.032856 | 0.14 |
| MYO9B | NM_004145 | 2166.05 | 1338.92 | 1.62 | 0.69 | Up | 0.046896 | 0.18 |
| HSPA6 | NM_002155 | 2727.82 | 1685.90 | 1.62 | 0.69 | Up | 0.032081 | 0.20 |
| HBB | NM_000518 | 255573.38 | 157896.66 | 1.62 | 0.69 | Up | 0.049996 | 0.18 |
| AK125099 | AK125099 | 951.52 | 587.83 | 1.62 | 0.69 | Up | 0.019852 | 0.10 |
| PLIN5 | NM_001013706 | 742.07 | 457.35 | 1.62 | 0.70 | Up | 0.015404 | 0.12 |
| HBB | NM_000518 | 254071.22 | 156545.83 | 1.62 | 0.70 | Up | 0.010768 | 0.10 |
| SEC14L1 | NM_003003 | 10630.45 | 6549.01 | 1.62 | 0.70 | Up | 0.020094 | 0.14 |
| SLC19A1 | NM_194255 | 1161.76 | 715.70 | 1.62 | 0.70 | Up | 0.046627 | 0.20 |
| TBL1X | NM_005647 | 1992.02 | 1226.88 | 1.62 | 0.70 | Up | 0.018855 | 0.10 |
| LILRB3 | NM_006864 | 34230.72 | 21071.99 | 1.62 | 0.70 | Up | 0.017673 | 0.12 |
| DOK3 | NM_024872 | 1342.06 | 826.15 | 1.62 | 0.70 | Up | 0.004136 | 0.08 |
| TPM4 | NM_003290 | 865.65 | 532.57 | 1.63 | 0.70 | Up | 0.000916 | 0.00 |
| CASP10 | NM_032977 | 2637.60 | 1620.19 | 1.63 | 0.70 | Up | 0.008576 | 0.06 |
| PPP1R14B | NM_138689 | 5240.02 | 3216.74 | 1.63 | 0.70 | Up | 0.005970 | 0.10 |
| RERE | NM_012102 | 545.10 | 334.40 | 1.63 | 0.70 | Up | 0.029480 | 0.16 |
| NPLOC4 | NM_017921 | 3682.34 | 2258.06 | 1.63 | 0.71 | Up | 0.038708 | 0.24 |
| XLOC_009524 | TCONS_00019731 | 310.10 | 190.13 | 1.63 | 0.71 | Up | 0.035645 | 0.12 |
| DENND3 | ENST00000520482 | 5212.25 | 3194.72 | 1.63 | 0.71 | Up | 0.004376 | 0.10 |
| ASAP1 | NM_001247996 | 634.38 | 388.50 | 1.63 | 0.71 | Up | 0.021365 | 0.16 |
| LTA4H | NM_000895 | 7400.92 | 4532.10 | 1.63 | 0.71 | Up | 0.031190 | 0.18 |
| ZFP36L1 | NM_004926 | 18669.38 | 11432.09 | 1.63 | 0.71 | Up | 0.021248 | 0.08 |
| PLEKHO2 | NM_025201 | 4183.21 | 2560.35 | 1.63 | 0.71 | Up | 0.011817 | 0.12 |
| XLOC_004924 | TCONS_00010404 | 4128.48 | 2526.73 | 1.63 | 0.71 | Up | 0.007447 | 0.08 |
| FBXO42 | NM_018994 | 1205.39 | 737.47 | 1.63 | 0.71 | Up | 0.028958 | 0.22 |
| DUX4 | NM_033178 | 301.69 | 184.53 | 1.63 | 0.71 | Up | 0.025958 | 0.16 |
| STAT1 | NM_139266 | 2888.70 | 1766.08 | 1.64 | 0.71 | Up | 0.034528 | 0.16 |
| NOTCH2 | NM_024408 | 4018.21 | 2454.11 | 1.64 | 0.71 | Up | 0.011175 | 0.10 |
| PLIN3 | NM_005817 | 464.70 | 283.69 | 1.64 | 0.71 | Up | 0.007274 | 0.10 |
| LYN | NM_002350 | 19759.13 | 12060.29 | 1.64 | 0.71 | Up | 0.015954 | 0.18 |
| ENST00000448179 | ENST00000448179 | 132798.41 | 80996.95 | 1.64 | 0.71 | Up | 0.003901 | 0.08 |
| THC2753069 | THC2753069 | 1396.42 | 851.66 | 1.64 | 0.71 | Up | 0.010625 | 0.10 |
| ADAM8 | NM_001109 | 36566.74 | 22291.61 | 1.64 | 0.71 | Up | 0.044667 | 0.18 |
| CSF1R | NM_005211 | 9478.75 | 5776.85 | 1.64 | 0.71 | Up | 0.039695 | 0.18 |
| LOC729175 | XM_001129558 | 474.28 | 288.74 | 1.64 | 0.72 | Up | 0.035147 | 0.16 |
| BBX | NM_020235 | 1620.07 | 986.14 | 1.64 | 0.72 | Up | 0.047525 | 0.20 |
| CCDC88B | NM_032251 | 13851.64 | 8423.84 | 1.64 | 0.72 | Up | 0.021952 | 0.14 |
| FES | NM_002005 | 1650.73 | 1003.81 | 1.64 | 0.72 | Up | 0.026951 | 0.16 |
| FSTL1 | NM_007085 | 116.56 | 70.83 | 1.65 | 0.72 | Up | 0.001315 | 0.06 |
| CSF3R | NM_156039 | 83814.93 | 50912.74 | 1.65 | 0.72 | Up | 0.013188 | 0.14 |
| BBX | NM_020235 | 1730.94 | 1051.09 | 1.65 | 0.72 | Up | 0.024767 | 0.20 |
| HBA2 | NM_000517 | 270909.71 | 164490.33 | 1.65 | 0.72 | Up | 0.006251 | 0.10 |
| HSPA1A | NM_005345 | 6829.88 | 4145.54 | 1.65 | 0.72 | Up | 0.047086 | 0.18 |
| LYN | NM_002350 | 19627.99 | 11904.25 | 1.65 | 0.72 | Up | 0.013927 | 0.12 |
| RPS2 | NM_002952 | 111378.62 | 67525.56 | 1.65 | 0.72 | Up | 0.016996 | 0.12 |
| DYNC1H1 | NM_001376 | 1542.72 | 934.87 | 1.65 | 0.72 | Up | 0.031947 | 0.14 |
| STAT1 | NM_139266 | 2805.30 | 1698.39 | 1.65 | 0.72 | Up | 0.030513 | 0.14 |
| FLOT2 | NM_004475 | 9375.44 | 5671.26 | 1.65 | 0.73 | Up | 0.015853 | 0.14 |
| ICAM1 | NM_000201 | 402.84 | 243.66 | 1.65 | 0.73 | Up | 0.034527 | 0.14 |
| CPAMD8 | NM_015692 | 446.79 | 270.15 | 1.65 | 0.73 | Up | 0.030917 | 0.16 |
| BAG1 | NM_001172415 | 2701.75 | 1633.55 | 1.65 | 0.73 | Up | 0.049692 | 0.20 |
| DISC1 | NM_001164554 | 623.17 | 376.72 | 1.65 | 0.73 | Up | 0.011193 | 0.12 |
| LYN | NM_002350 | 22058.53 | 13332.80 | 1.65 | 0.73 | Up | 0.017506 | 0.14 |
| XLOC_l2_013594 | BQ926105 | 885.32 | 534.38 | 1.66 | 0.73 | Up | 0.005839 | 0.12 |
| A_24_P636974 | A_24_P636974 | 212.67 | 128.31 | 1.66 | 0.73 | Up | 0.034312 | 0.12 |
| NFKB1 | NM_003998 | 1467.47 | 884.29 | 1.66 | 0.73 | Up | 0.038101 | 0.16 |
| PHC2 | NM_198040 | 11442.48 | 6894.34 | 1.66 | 0.73 | Up | 0.028865 | 0.18 |
| IL2RG | NM_000206 | 7274.11 | 4374.34 | 1.66 | 0.73 | Up | 0.037674 | 0.20 |
| TMEM175 | NM_032326 | 3307.08 | 1986.70 | 1.66 | 0.74 | Up | 0.047940 | 0.22 |
| ITGAM | NM_000632 | 2005.78 | 1204.17 | 1.67 | 0.74 | Up | 0.029285 | 0.18 |
| ACTN4 | NM_004924 | 927.55 | 556.43 | 1.67 | 0.74 | Up | 0.021202 | 0.08 |
| RUFY1 | NM_025158 | 4810.16 | 2884.48 | 1.67 | 0.74 | Up | 0.003718 | 0.10 |
| C10orf54 | NM_022153 | 17201.34 | 10311.31 | 1.67 | 0.74 | Up | 0.033434 | 0.18 |
| CTSB | NM_147780 | 2647.38 | 1586.28 | 1.67 | 0.74 | Up | 0.035514 | 0.16 |
| LOC283070 | NR_027322 | 3769.78 | 2257.85 | 1.67 | 0.74 | Up | 0.037525 | 0.16 |
| XLOC_l2_001399 | ENST00000411733 | 865.76 | 518.50 | 1.67 | 0.74 | Up | 0.004001 | 0.10 |
| ITGAM | NM_000632 | 2001.29 | 1198.15 | 1.67 | 0.74 | Up | 0.046674 | 0.16 |
| TFF1 | NM_003225 | 248.11 | 148.48 | 1.67 | 0.74 | Up | 0.044845 | 0.14 |
| GK | ENST00000378938 | 193.66 | 115.85 | 1.67 | 0.74 | Up | 0.039621 | 0.18 |
| DHRS13 | NM_144683 | 1300.55 | 777.72 | 1.67 | 0.74 | Up | 0.015784 | 0.12 |
| CSNK1D | NM_001893 | 667.48 | 398.88 | 1.67 | 0.74 | Up | 0.040689 | 0.14 |
| SPI1 | NM_001080547 | 11685.69 | 6982.22 | 1.67 | 0.74 | Up | 0.013799 | 0.16 |
| LOC729040 | XR_108595 | 369.99 | 220.95 | 1.67 | 0.74 | Up | 0.007981 | 0.08 |
| RNF24 | NM_007219 | 1068.69 | 637.40 | 1.68 | 0.75 | Up | 0.015192 | 0.16 |
| SLC8A1 | NM_021097 | 869.09 | 518.13 | 1.68 | 0.75 | Up | 0.018093 | 0.16 |
| LINC00174 | NR_026873 | 1138.78 | 678.85 | 1.68 | 0.75 | Up | 0.036349 | 0.20 |
| ICAM1 | NM_000201 | 388.14 | 231.01 | 1.68 | 0.75 | Up | 0.045131 | 0.18 |
| TNFRSF1A | NM_001065 | 2563.82 | 1525.65 | 1.68 | 0.75 | Up | 0.040194 | 0.16 |
| THBS1 | NM_003246 | 206.27 | 122.68 | 1.68 | 0.75 | Up | 0.020440 | 0.08 |
| DB335107 | DB335107 | 2535.68 | 1507.98 | 1.68 | 0.75 | Up | 0.010196 | 0.12 |
| TLR5 | NM_003268 | 524.93 | 311.91 | 1.68 | 0.75 | Up | 0.031877 | 0.08 |
| EIF3B | NM_001037283 | 1809.90 | 1075.18 | 1.68 | 0.75 | Up | 0.040836 | 0.22 |
| LOC401357 | XR_132811 | 3100.97 | 1841.06 | 1.68 | 0.75 | Up | 0.031895 | 0.16 |
| XLOC_l2_000297 | THC2520127 | 326.03 | 193.44 | 1.69 | 0.75 | Up | 0.024958 | 0.14 |
| HK1 | NM_033500 | 1487.58 | 882.19 | 1.69 | 0.75 | Up | 0.030768 | 0.16 |
| XLOC_002730 | TCONS_00006915 | 377.32 | 223.68 | 1.69 | 0.75 | Up | 0.046248 | 0.14 |
| IL1RN | NM_173843 | 13126.44 | 7778.74 | 1.69 | 0.75 | Up | 0.025361 | 0.16 |
| PAQR6 | NM_024897 | 782.29 | 463.41 | 1.69 | 0.76 | Up | 0.004484 | 0.04 |
| SEC31A | NM_001077207 | 1164.86 | 689.78 | 1.69 | 0.76 | Up | 0.016235 | 0.12 |
| HSCB | NM_172002 | 53568.46 | 31714.55 | 1.69 | 0.76 | Up | 0.009619 | 0.14 |
| PLIN3 | NM_005817 | 582.59 | 344.63 | 1.69 | 0.76 | Up | 0.004854 | 0.10 |
| TMEM88 | NM_203411 | 609.64 | 360.61 | 1.69 | 0.76 | Up | 0.003788 | 0.06 |
| SBNO1 | NM_001167856 | 1146.23 | 677.96 | 1.69 | 0.76 | Up | 0.021235 | 0.18 |
| CD163 | NM_004244 | 1057.28 | 624.66 | 1.69 | 0.76 | Up | 0.037816 | 0.18 |
| EXTL3 | NM_001440 | 4228.43 | 2494.88 | 1.69 | 0.76 | Up | 0.024157 | 0.18 |
| TMEM40 | NM_018306 | 172.13 | 101.45 | 1.70 | 0.76 | Up | 0.038366 | 0.16 |
| C15orf39 | NM_015492 | 1475.85 | 869.75 | 1.70 | 0.76 | Up | 0.014883 | 0.12 |
| LOC497256 | AK094988 | 199.83 | 117.73 | 1.70 | 0.76 | Up | 0.018710 | 0.12 |
| XLOC_l2_000727 | THC2520724 | 235.54 | 138.59 | 1.70 | 0.77 | Up | 0.010929 | 0.12 |
| A_33_P3303394 | A_33_P3303394 | 57164.03 | 33630.70 | 1.70 | 0.77 | Up | 0.020367 | 0.16 |
| ITGAM | NM_000632 | 1668.11 | 981.10 | 1.70 | 0.77 | Up | 0.027964 | 0.18 |
| LYN | NM_002350 | 18433.04 | 10829.88 | 1.70 | 0.77 | Up | 0.016079 | 0.12 |
| DAPK3 | NM_001348 | 2339.06 | 1373.73 | 1.70 | 0.77 | Up | 0.025044 | 0.18 |
| N4BP1 | NM_153029 | 656.29 | 385.28 | 1.70 | 0.77 | Up | 0.012116 | 0.10 |
| TNFSF10 | NM_003810 | 5137.46 | 3014.04 | 1.70 | 0.77 | Up | 0.046684 | 0.22 |
| XLOC_l2_005350 | ENST00000446912 | 1062.90 | 623.23 | 1.71 | 0.77 | Up | 0.003016 | 0.08 |
| HBD | NM_000519 | 211465.49 | 123914.21 | 1.71 | 0.77 | Up | 0.007941 | 0.10 |
| FLOT1 | NM_005803 | 3197.19 | 1870.17 | 1.71 | 0.77 | Up | 0.001919 | 0.08 |
| SEC24C | NM_004922 | 6709.32 | 3924.19 | 1.71 | 0.77 | Up | 0.045696 | 0.20 |
| LOC731275 | NR_029401 | 1448.10 | 846.94 | 1.71 | 0.77 | Up | 0.031881 | 0.16 |
| CLDN5 | NM_001130861 | 413.00 | 241.40 | 1.71 | 0.77 | Up | 0.003989 | 0.08 |
| PLIN3 | NM_005817 | 475.35 | 277.59 | 1.71 | 0.78 | Up | 0.005259 | 0.10 |
| CYP1B1 | NM_000104 | 1171.16 | 683.64 | 1.71 | 0.78 | Up | 0.013106 | 0.12 |
| APOL2 | NM_145637 | 6694.38 | 3906.78 | 1.71 | 0.78 | Up | 0.004048 | 0.10 |
| ETV6 | NM_001987 | 432.10 | 252.12 | 1.71 | 0.78 | Up | 0.018182 | 0.10 |
| NRG1 | AF176921 | 89.44 | 52.14 | 1.72 | 0.78 | Up | 0.026585 | 0.16 |
| LYN | NM_002350 | 25939.70 | 15108.97 | 1.72 | 0.78 | Up | 0.008424 | 0.12 |
| KIAA1324 | NM_020775 | 1482.01 | 863.00 | 1.72 | 0.78 | Up | 0.047788 | 0.18 |
| YBX1 | NM_004559 | 8262.86 | 4810.22 | 1.72 | 0.78 | Up | 0.003515 | 0.00 |
| PRKCD | NM_006254 | 2785.50 | 1620.96 | 1.72 | 0.78 | Up | 0.047019 | 0.18 |
| ABCD1 | NM_000033 | 298.08 | 173.45 | 1.72 | 0.78 | Up | 0.006930 | 0.14 |
| ITGAM | NM_000632 | 1766.85 | 1026.95 | 1.72 | 0.78 | Up | 0.028560 | 0.20 |
| ITGAM | NM_000632 | 1695.64 | 984.04 | 1.72 | 0.79 | Up | 0.035003 | 0.18 |
| TBL1Y | NM_033284 | 657.79 | 381.42 | 1.72 | 0.79 | Up | 0.009264 | 0.08 |
| CASP1 | NM_033292 | 3739.94 | 2162.92 | 1.73 | 0.79 | Up | 0.020365 | 0.16 |
| IL17RA | NM_014339 | 3134.96 | 1809.13 | 1.73 | 0.79 | Up | 0.015176 | 0.16 |
| KIAA0513 | NM_014732 | 6228.43 | 3588.72 | 1.74 | 0.80 | Up | 0.037013 | 0.16 |
| XLOC_l2_013383 | TCONS_l2_00025852 | 289.65 | 166.73 | 1.74 | 0.80 | Up | 0.001784 | 0.04 |
| XM_003118986 | XM_003118986 | 7388.90 | 4250.53 | 1.74 | 0.80 | Up | 0.024866 | 0.16 |
| ARRB2 | NM_004313 | 14408.94 | 8288.74 | 1.74 | 0.80 | Up | 0.002837 | 0.10 |
| LOC100505994 | XR_108596 | 899.25 | 517.06 | 1.74 | 0.80 | Up | 0.018381 | 0.10 |
| PLIN3 | NM_005817 | 534.90 | 307.51 | 1.74 | 0.80 | Up | 0.005955 | 0.08 |
| SLC15A3 | NM_016582 | 3443.63 | 1978.60 | 1.74 | 0.80 | Up | 0.018691 | 0.12 |
| ALDH2 | NM_000690 | 1438.95 | 826.06 | 1.74 | 0.80 | Up | 0.048240 | 0.18 |
| LOC731275 | NR_029401 | 5848.98 | 3357.72 | 1.74 | 0.80 | Up | 0.001779 | 0.04 |
| FKBP1B | NM_054033 | 412.33 | 236.29 | 1.75 | 0.80 | Up | 0.029222 | 0.14 |
| LOC100288069 | NR_033908 | 1941.04 | 1110.86 | 1.75 | 0.81 | Up | 0.004620 | 0.06 |
| PRR5 | NM_015366 | 4709.51 | 2694.02 | 1.75 | 0.81 | Up | 0.022470 | 0.16 |
| PQLC2 | NM_001040125 | 169324.09 | 96789.77 | 1.75 | 0.81 | Up | 0.012787 | 0.12 |
| CRISPLD2 | NM_031476 | 181.04 | 103.48 | 1.75 | 0.81 | Up | 0.019849 | 0.16 |
| GLT1D1 | NM_144669 | 5174.61 | 2956.05 | 1.75 | 0.81 | Up | 0.023270 | 0.16 |
| TNFRSF1A | NM_001065 | 2647.28 | 1510.26 | 1.75 | 0.81 | Up | 0.031483 | 0.16 |
| GNAZ | NM_002073 | 962.23 | 547.29 | 1.76 | 0.81 | Up | 0.033819 | 0.12 |
| HBB | NM_000518 | 270727.03 | 153847.21 | 1.76 | 0.82 | Up | 0.003294 | 0.08 |
| PLCB2 | NM_004573 | 17203.01 | 9772.05 | 1.76 | 0.82 | Up | 0.027097 | 0.18 |
| GIMAP8 | NM_175571 | 1210.23 | 687.29 | 1.76 | 0.82 | Up | 0.037779 | 0.20 |
| SLC25A44 | NM_014655 | 2372.80 | 1347.03 | 1.76 | 0.82 | Up | 0.018342 | 0.14 |
| DOCK8 | NM_203447 | 3667.62 | 2080.12 | 1.76 | 0.82 | Up | 0.035804 | 0.16 |
| NFKB1 | NM_003998 | 1425.63 | 806.77 | 1.77 | 0.82 | Up | 0.035126 | 0.16 |
| SPARC | NM_003118 | 148.71 | 84.15 | 1.77 | 0.82 | Up | 0.017615 | 0.12 |
| LYN | NM_002350 | 20492.41 | 11592.85 | 1.77 | 0.82 | Up | 0.015821 | 0.14 |
| ACADVL | NM_000018 | 5172.65 | 2923.68 | 1.77 | 0.82 | Up | 0.033586 | 0.20 |
| ENST00000381524 | ENST00000381524 | 3847.26 | 2173.90 | 1.77 | 0.82 | Up | 0.010654 | 0.08 |
| LOC100128348 | AK128128 | 256147.62 | 144701.80 | 1.77 | 0.82 | Up | 0.007616 | 0.10 |
| THC2572285 | THC2572285 | 14633.34 | 8265.98 | 1.77 | 0.82 | Up | 0.022930 | 0.14 |
| ACSL1 | NM_001995 | 4314.93 | 2425.38 | 1.78 | 0.83 | Up | 0.038597 | 0.14 |
| LYN | NM_002350 | 24817.38 | 13936.39 | 1.78 | 0.83 | Up | 0.010429 | 0.12 |
| XLOC_l2_013420 | TCONS_l2_00025900 | 34442.78 | 19338.54 | 1.78 | 0.83 | Up | 0.048193 | 0.20 |
| ACSL1 | NM_001995 | 3620.10 | 2032.37 | 1.78 | 0.83 | Up | 0.041274 | 0.14 |
| PXN | NM_002859 | 24928.45 | 13963.90 | 1.79 | 0.84 | Up | 0.010021 | 0.08 |
| PRDM1 | NM_001198 | 346.48 | 193.77 | 1.79 | 0.84 | Up | 0.003001 | 0.04 |
| UCP3 | NM_022803 | 2418.82 | 1352.48 | 1.79 | 0.84 | Up | 0.019892 | 0.12 |
| TBL1Y | NM_033284 | 586.45 | 327.87 | 1.79 | 0.84 | Up | 0.049152 | 0.14 |
| SORL1 | NM_003105 | 39630.29 | 22151.45 | 1.79 | 0.84 | Up | 0.008516 | 0.04 |
| CBS | NM_000071 | 457.27 | 255.07 | 1.79 | 0.84 | Up | 0.042958 | 0.20 |
| HSPA1A | NM_005345 | 6430.18 | 3585.70 | 1.79 | 0.84 | Up | 0.044697 | 0.16 |
| FLJ14186 | NR_037596 | 3580.86 | 1995.31 | 1.79 | 0.84 | Up | 0.025478 | 0.18 |
| AB529256 | AB529256 | 202.69 | 112.90 | 1.80 | 0.84 | Up | 0.046900 | 0.12 |
| STAT2 | NM_005419 | 9108.43 | 5070.83 | 1.80 | 0.84 | Up | 0.009111 | 0.12 |
| S100A8 | NM_002964 | 31807.12 | 17662.57 | 1.80 | 0.85 | Up | 0.017149 | 0.12 |
| APOL1 | NM_145343 | 1765.94 | 980.27 | 1.80 | 0.85 | Up | 0.010160 | 0.16 |
| XLOC_006193 | AK055267 | 2209.11 | 1224.38 | 1.80 | 0.85 | Up | 0.026049 | 0.18 |
| NAALADL2 | NM_207015 | 292.70 | 162.18 | 1.80 | 0.85 | Up | 0.044661 | 0.16 |
| NFKB1 | NM_003998 | 1707.87 | 944.26 | 1.81 | 0.85 | Up | 0.028222 | 0.14 |
| ALOX5 | NM_000698 | 19620.14 | 10841.50 | 1.81 | 0.86 | Up | 0.029976 | 0.20 |
| NEAT1 | AF001893 | 1564.37 | 863.85 | 1.81 | 0.86 | Up | 0.031997 | 0.04 |
| PRIC285 | NM_001037335 | 4043.47 | 2232.34 | 1.81 | 0.86 | Up | 0.006325 | 0.12 |
| TNFRSF1A | NM_001065 | 2952.33 | 1629.27 | 1.81 | 0.86 | Up | 0.042107 | 0.22 |
| LOC100288069 | NR_033908 | 2304.84 | 1271.53 | 1.81 | 0.86 | Up | 0.003471 | 0.06 |
| TNFRSF1A | NM_001065 | 2761.99 | 1521.77 | 1.81 | 0.86 | Up | 0.035380 | 0.18 |
| NFKB1 | NM_003998 | 1682.18 | 923.35 | 1.82 | 0.87 | Up | 0.029760 | 0.16 |
| EMILIN2 | NM_032048 | 2866.84 | 1570.68 | 1.83 | 0.87 | Up | 0.026187 | 0.16 |
| SERPINA1 | NM_001002236 | 25780.78 | 14118.14 | 1.83 | 0.87 | Up | 0.006262 | 0.10 |
| APBB1IP | NM_019043 | 4365.52 | 2388.40 | 1.83 | 0.87 | Up | 0.009128 | 0.10 |
| NFE2 | NM_006163 | 23816.34 | 12988.62 | 1.83 | 0.87 | Up | 0.009484 | 0.10 |
| SECTM1 | NM_003004 | 13285.75 | 7243.48 | 1.83 | 0.88 | Up | 0.028894 | 0.24 |
| XLOC_012841 | TCONS_00026520 | 1377.04 | 749.31 | 1.84 | 0.88 | Up | 0.044703 | 0.20 |
| IL17RA | NM_014339 | 5813.74 | 3162.71 | 1.84 | 0.88 | Up | 0.031124 | 0.18 |
| PGM1 | NM_002633 | 1138.38 | 618.64 | 1.84 | 0.88 | Up | 0.007047 | 0.10 |
| LINC00265 | NR_026999 | 18575.01 | 10086.55 | 1.84 | 0.88 | Up | 0.010772 | 0.12 |
| NEBL | NM_006393 | 72.89 | 39.50 | 1.85 | 0.88 | Up | 0.006178 | 0.08 |
| TALDO1 | NM_006755 | 38655.02 | 20922.60 | 1.85 | 0.89 | Up | 0.027461 | 0.14 |
| APP | NM_000484 | 320.84 | 173.60 | 1.85 | 0.89 | Up | 0.018333 | 0.14 |
| PARP9 | NM_001146106 | 1251.90 | 677.35 | 1.85 | 0.89 | Up | 0.015467 | 0.16 |
| NKX1-2 | NM_001146340 | 20195.28 | 10913.62 | 1.85 | 0.89 | Up | 0.025293 | 0.20 |
| NFKB1 | NM_003998 | 1810.71 | 975.16 | 1.86 | 0.89 | Up | 0.036435 | 0.12 |
| ALOX5 | NM_000698 | 20827.72 | 11193.19 | 1.86 | 0.90 | Up | 0.044124 | 0.20 |
| CR1L | NM_175710 | 182.45 | 97.99 | 1.86 | 0.90 | Up | 0.000480 | 0.00 |
| A_33_P3304748 | A_33_P3304748 | 1808.85 | 969.43 | 1.87 | 0.90 | Up | 0.012396 | 0.04 |
| ACSL1 | NM_001995 | 3673.55 | 1968.11 | 1.87 | 0.90 | Up | 0.040523 | 0.14 |
| LOC401357 | XR_132464 | 16897.98 | 9052.18 | 1.87 | 0.90 | Up | 0.014291 | 0.14 |
| ITGB5 | NM_002213 | 185.03 | 98.88 | 1.87 | 0.90 | Up | 0.027699 | 0.12 |
| LOC100133331 | NR_028327 | 1078.36 | 576.26 | 1.87 | 0.90 | Up | 0.016471 | 0.08 |
| PLIN3 | NM_005817 | 555.92 | 297.02 | 1.87 | 0.90 | Up | 0.003923 | 0.06 |
| SBNO2 | NM_014963 | 7369.77 | 3936.22 | 1.87 | 0.90 | Up | 0.011471 | 0.14 |
| OSCAR | NM_206818 | 3873.02 | 2066.61 | 1.87 | 0.91 | Up | 0.008347 | 0.08 |
| PADI2 | NM_007365 | 742.42 | 393.53 | 1.89 | 0.92 | Up | 0.033171 | 0.16 |
| EIF2AK2 | NM_002759 | 1695.38 | 894.60 | 1.90 | 0.92 | Up | 0.048544 | 0.16 |
| FLJ45445 | NR_028324 | 22313.63 | 11767.13 | 1.90 | 0.92 | Up | 0.016668 | 0.16 |
| LOC729737 | NR_039983 | 896.71 | 472.17 | 1.90 | 0.93 | Up | 0.002559 | 0.04 |
| SLC2A14 | ENST00000431042 | 4039.48 | 2121.21 | 1.90 | 0.93 | Up | 0.006198 | 0.10 |
| UBN1 | NM_016936 | 2989.00 | 1567.91 | 1.91 | 0.93 | Up | 0.024002 | 0.10 |
| ACSL1 | NM_001995 | 4298.83 | 2254.97 | 1.91 | 0.93 | Up | 0.041043 | 0.16 |
| SORT1 | NM_002959 | 786.11 | 412.28 | 1.91 | 0.93 | Up | 0.024380 | 0.18 |
| LOC399744 | NR_024497 | 857.47 | 447.04 | 1.92 | 0.94 | Up | 0.028008 | 0.10 |
| PLIN3 | NM_005817 | 629.62 | 328.03 | 1.92 | 0.94 | Up | 0.005897 | 0.08 |
| MYH9 | NM_002473 | 5612.75 | 2922.47 | 1.92 | 0.94 | Up | 0.002444 | 0.02 |
| ENST00000419160 | ENST00000419160 | 165.22 | 85.99 | 1.92 | 0.94 | Up | 0.008586 | 0.10 |
| LOC731275 | NR_029401 | 5032.99 | 2614.43 | 1.93 | 0.94 | Up | 0.000755 | 0.06 |
| MARCH2 | NM_016496 | 628.65 | 325.17 | 1.93 | 0.95 | Up | 0.031317 | 0.10 |
| A_33_P3292126 | A_33_P3292126 | 2171.78 | 1122.25 | 1.94 | 0.95 | Up | 0.046551 | 0.18 |
| SIPA1L1 | NM_015556 | 1829.57 | 944.99 | 1.94 | 0.95 | Up | 0.012681 | 0.14 |
| TRIM25 | NM_005082 | 7637.30 | 3939.73 | 1.94 | 0.95 | Up | 0.014307 | 0.14 |
| XLOC_l2_014931 | ENST00000422343 | 1030.26 | 531.45 | 1.94 | 0.96 | Up | 0.043945 | 0.16 |
| XLOC_004346 | TCONS_00009926 | 333.58 | 172.00 | 1.94 | 0.96 | Up | 0.036591 | 0.12 |
| LYN | NM_002350 | 19400.39 | 9995.21 | 1.94 | 0.96 | Up | 0.011750 | 0.12 |
| LOC731275 | NR_029401 | 808.46 | 415.81 | 1.94 | 0.96 | Up | 0.004511 | 0.04 |
| IRF7 | NM_004031 | 8953.41 | 4593.46 | 1.95 | 0.96 | Up | 0.021837 | 0.16 |
| HSPA1A | NM_005345 | 6529.72 | 3345.50 | 1.95 | 0.96 | Up | 0.035103 | 0.18 |
| APOBR | NM_018690 | 4120.97 | 2107.06 | 1.96 | 0.97 | Up | 0.019892 | 0.14 |
| FAM129A | NM_052966 | 9308.00 | 4750.02 | 1.96 | 0.97 | Up | 0.019659 | 0.14 |
| SEPT14 | NM_207366 | 1228.61 | 625.18 | 1.97 | 0.97 | Up | 0.036576 | 0.12 |
| A_33_P3221055 | A_33_P3221055 | 1801.84 | 910.71 | 1.98 | 0.98 | Up | 0.034073 | 0.16 |
| GK | ENST00000378941 | 196.05 | 99.00 | 1.98 | 0.99 | Up | 0.000003 | 0.00 |
| XLOC_006324 | ENST00000469418 | 546.40 | 275.47 | 1.98 | 0.99 | Up | 0.004408 | 0.04 |
| STAT1 | NM_139266 | 2935.69 | 1478.67 | 1.99 | 0.99 | Up | 0.018595 | 0.12 |
| OSM | NM_020530 | 581.85 | 291.37 | 2.00 | 1.00 | Up | 0.041370 | 0.14 |
| DKFZP434F142 | AL136837 | 15200.56 | 7604.47 | 2.00 | 1.00 | Up | 0.032281 | 0.18 |
| SELL | NM_000655 | 31194.94 | 15602.46 | 2.00 | 1.00 | Up | 0.001100 | 0.04 |
| LOC100288069 | NR_033908 | 481.94 | 240.97 | 2.00 | 1.00 | Up | 0.007204 | 0.08 |
| BAG1 | NM_001172415 | 13528.83 | 6756.55 | 2.00 | 1.00 | Up | 0.038826 | 0.12 |
| LOC100653060 | XM_003403393 | 431.97 | 215.40 | 2.01 | 1.00 | Up | 0.017785 | 0.12 |
| LYN | NM_002350 | 20514.26 | 10228.58 | 2.01 | 1.00 | Up | 0.009932 | 0.10 |
| XLOC_l2_015938 | TCONS_l2_00030929 | 8809.12 | 4382.26 | 2.01 | 1.01 | Up | 0.034875 | 0.12 |
| NEBL | NM_006393 | 63.43 | 31.52 | 2.01 | 1.01 | Up | 0.023060 | 0.24 |
| NFKB1 | NM_003998 | 1615.79 | 802.80 | 2.01 | 1.01 | Up | 0.027429 | 0.12 |
| LIMK2 | NM_016733 | 7795.53 | 3861.83 | 2.02 | 1.01 | Up | 0.018621 | 0.12 |
| CLU | NM_001831 | 836.51 | 413.13 | 2.02 | 1.02 | Up | 0.042327 | 0.22 |
| GNL3L | NM_019067 | 5954.61 | 2934.43 | 2.03 | 1.02 | Up | 0.032513 | 0.20 |
| GBP2 | NM_004120 | 2880.78 | 1411.74 | 2.04 | 1.03 | Up | 0.009735 | 0.08 |
| LOC731275 | NR_029401 | 1082.52 | 529.07 | 2.05 | 1.03 | Up | 0.000833 | 0.02 |
| SOCS3 | NM_003955 | 708.26 | 345.92 | 2.05 | 1.03 | Up | 0.047206 | 0.20 |
| ACSL1 | NM_001995 | 3662.76 | 1788.55 | 2.05 | 1.03 | Up | 0.042299 | 0.16 |
| MMP9 | NM_004994 | 9362.43 | 4569.53 | 2.05 | 1.03 | Up | 0.045560 | 0.20 |
| CTNNA1 | NM_001903 | 542.33 | 264.28 | 2.05 | 1.04 | Up | 0.030932 | 0.16 |
| CXCL5 | NM_002994 | 471.95 | 228.28 | 2.07 | 1.05 | Up | 0.045531 | 0.18 |
| XLOC_006188 | TCONS_00014180 | 26589.70 | 12852.23 | 2.07 | 1.05 | Up | 0.048570 | 0.22 |
| MMP9 | NM_004994 | 9390.90 | 4528.25 | 2.07 | 1.05 | Up | 0.039070 | 0.16 |
| APBB1IP | NM_019043 | 15424.86 | 7421.72 | 2.08 | 1.06 | Up | 0.013011 | 0.12 |
| MVP | NM_017458 | 23967.71 | 11503.56 | 2.08 | 1.06 | Up | 0.004570 | 0.08 |
| RNF222 | NM_001146684 | 1421.34 | 679.93 | 2.09 | 1.06 | Up | 0.033565 | 0.14 |
| BAG1 | NM_004323 | 17506.29 | 8373.49 | 2.09 | 1.06 | Up | 0.025233 | 0.14 |
| HSPA1A | NM_005345 | 6292.40 | 3003.18 | 2.10 | 1.07 | Up | 0.039402 | 0.20 |
| LOC100287633 | XM_002342728 | 2816.64 | 1343.80 | 2.10 | 1.07 | Up | 0.030924 | 0.22 |
| STAT1 | NM_139266 | 3152.95 | 1502.77 | 2.10 | 1.07 | Up | 0.018043 | 0.14 |
| GRN | NM_002087 | 58318.30 | 27740.08 | 2.10 | 1.07 | Up | 0.025838 | 0.16 |
| CD14 | NM_001174104 | 33659.36 | 16008.56 | 2.10 | 1.07 | Up | 0.033178 | 0.16 |
| MX2 | NM_002463 | 6188.26 | 2937.57 | 2.11 | 1.07 | Up | 0.015737 | 0.12 |
| C20orf108 | ENST00000437418 | 1363.00 | 641.46 | 2.12 | 1.09 | Up | 0.022879 | 0.16 |
| MMP9 | NM_004994 | 9379.65 | 4412.73 | 2.13 | 1.09 | Up | 0.034680 | 0.14 |
| MMP9 | NM_004994 | 8664.92 | 4055.19 | 2.14 | 1.10 | Up | 0.045130 | 0.18 |
| XLOC_008609 | TCONS_00018331 | 1011.61 | 471.83 | 2.14 | 1.10 | Up | 0.034828 | 0.18 |
| MMP9 | NM_004994 | 8340.98 | 3883.54 | 2.15 | 1.10 | Up | 0.038852 | 0.16 |
| THC2767054 | THC2767054 | 389.97 | 181.46 | 2.15 | 1.10 | Up | 0.017268 | 0.14 |
| XLOC_l2_001332 | TCONS_l2_00001807 | 360.50 | 167.69 | 2.15 | 1.10 | Up | 0.026005 | 0.18 |
| FLJ45340 | NR_024368 | 3836.72 | 1777.29 | 2.16 | 1.11 | Up | 0.024807 | 0.12 |
| MMP9 | NM_004994 | 8699.59 | 4011.49 | 2.17 | 1.12 | Up | 0.036231 | 0.18 |
| A_33_P3390673 | A_33_P3390673 | 1853.58 | 850.06 | 2.18 | 1.12 | Up | 0.017439 | 0.14 |
| ACSL1 | NM_001995 | 4637.35 | 2120.80 | 2.19 | 1.13 | Up | 0.042373 | 0.14 |
| CTTN | NM_005231 | 727.22 | 329.60 | 2.21 | 1.14 | Up | 0.026835 | 0.16 |
| MFN2 | NM_014874 | 4694.03 | 2117.57 | 2.22 | 1.15 | Up | 0.028773 | 0.20 |
| LOC400499 | ENST00000344649 | 345.35 | 155.77 | 2.22 | 1.15 | Up | 0.007592 | 0.04 |
| LIMK2 | NM_001031801 | 634.56 | 285.91 | 2.22 | 1.15 | Up | 0.018130 | 0.14 |
| STAT1 | NM_139266 | 3109.39 | 1399.38 | 2.22 | 1.15 | Up | 0.016096 | 0.12 |
| RNF213 | NM_020914 | 358.46 | 161.02 | 2.23 | 1.15 | Up | 0.037702 | 0.18 |
| STAT1 | NM_139266 | 2885.75 | 1295.54 | 2.23 | 1.16 | Up | 0.031085 | 0.16 |
| NRGN | NM_006176 | 6611.52 | 2943.17 | 2.25 | 1.17 | Up | 0.035553 | 0.12 |
| CRYAA | NM_000394 | 4714.92 | 2016.40 | 2.34 | 1.23 | Up | 0.032902 | 0.18 |
| ZAN | NM_173059 | 632.03 | 269.90 | 2.34 | 1.23 | Up | 0.042321 | 0.18 |
| STAT1 | NM_139266 | 3978.48 | 1684.80 | 2.36 | 1.24 | Up | 0.015072 | 0.10 |
| TRANK1 | NM_014831 | 1860.47 | 784.18 | 2.37 | 1.25 | Up | 0.001344 | 0.02 |
| SNORD104 | NR_004380 | 1320.90 | 546.50 | 2.42 | 1.27 | Up | 0.047634 | 0.16 |
| LRG1 | NM_052972 | 2263.10 | 922.80 | 2.45 | 1.29 | Up | 0.018121 | 0.16 |
| IFIT2 | NM_001547 | 1460.84 | 565.72 | 2.58 | 1.37 | Up | 0.031747 | 0.14 |
| CCR1 | NM_001295 | 7713.61 | 2898.24 | 2.66 | 1.41 | Up | 0.021703 | 0.12 |
| DYSF | NM_003494 | 1437.46 | 525.19 | 2.74 | 1.45 | Up | 0.014909 | 0.08 |
| MX1 | NM_002462 | 7612.81 | 2637.29 | 2.89 | 1.53 | Up | 0.038333 | 0.16 |
| ITGB3 | NM_000212 | 602.10 | 166.11 | 3.62 | 1.86 | Up | 0.039158 | 0.08 |
| MYOM2 | NM_003970 | 332.85 | 90.77 | 3.67 | 1.87 | Up | 0.037239 | 0.14 |
| HBZ | NM_005332 | 417.65 | 88.39 | 4.73 | 2.24 | Up | 0.013201 | 0.16 |
| HBZ | NM_005332 | 927.33 | 187.85 | 4.94 | 2.30 | Up | 0.009466 | 0.14 |
| SIRPB1 | NM_001135844 | 2527.12 | 497.45 | 5.08 | 2.34 | Up | 0.047447 | 0.16 |

Each value represents the median of 7 MFS patients and 7 HVs controls and ±standard deviation (STDV). Statistical analysis was performed with unpaired-two-tailed t-test (P < 0.05). MFS. Marfan syndrome; HVs. healthy volunteers; AUC. Area Under the Curve

**Table S3:** Significantly deregulated protein coding genes in the serum of patients with Marfan Syndrom (MFS) (n = 7) compared to healthy volunteer (HV) controls (n = 7) as determined by microarray analysis (P-values <0.05).

| **Gene Name** | **Systematic Name** | **Median MFS** | **Median HVs** | **Fold Change** | **Log2 Fold Change** | **Regulation** | **P-value** | **AUC** |
| --- | --- | --- | --- | --- | --- | --- | --- | --- |
| POT1 | NM_015450 | 248.67 | 530.62 | 0.47 | -1.09 | **Down** | 0.000168 | 1.00 |
| C17orf97 | NM_001013672 | 61.69 | 112.85 | 0.55 | -0.87 | **Down** | 0.026108 | 0.80 |
| FCER1A | NM_002001 | 560.85 | 908.25 | 0.62 | -0.70 | **Down** | 0.023762 | 0.88 |
| ARHGEF15 | NM_173728 | 287.81 | 453.65 | 0.63 | -0.66 | **Down** | 0.042069 | 0.80 |
| ME3 | NM_001014811 | 87.20 | 130.90 | 0.67 | -0.59 | **Down** | 0.039721 | 0.80 |
| RPS2 | NM_002952 | 177799.76 | 118882.72 | 1.50 | 0.58 | Up | 0.034702 | 0.16 |
| ACOX1 | NM_001185039 | 1964.04 | 1313.10 | 1.50 | 0.58 | Up | 0.021373 | 0.14 |
| CRISPLD2 | NM_031476 | 172.58 | 115.38 | 1.50 | 0.58 | Up | 0.025060 | 0.16 |
| NUB1 | NM_016118 | 929.94 | 621.64 | 1.50 | 0.58 | Up | 0.018410 | 0.14 |
| CD97 | NM_078481 | 17074.58 | 11399.16 | 1.50 | 0.58 | Up | 0.045424 | 0.14 |
| MYLK | NM_053025 | 152.00 | 101.43 | 1.50 | 0.58 | Up | 0.017457 | 0.08 |
| GPSM3 | NM_022107 | 8219.77 | 5484.36 | 1.50 | 0.58 | Up | 0.030723 | 0.16 |
| JAK3 | NM_000215 | 11611.30 | 7744.18 | 1.50 | 0.58 | Up | 0.038115 | 0.20 |
| REEP4 | NM_025232 | 369.19 | 245.94 | 1.50 | 0.59 | Up | 0.023934 | 0.16 |
| HLA-DRB1 | NM_002124 | 17676.69 | 11768.64 | 1.50 | 0.59 | Up | 0.016528 | 0.14 |
| ACTB | NM_001101 | 148311.47 | 98698.04 | 1.50 | 0.59 | Up | 0.001192 | 0.06 |
| PMPCA | NM_015160 | 642.31 | 427.34 | 1.50 | 0.59 | Up | 0.044147 | 0.22 |
| AATF | NM_012138 | 1135.29 | 755.31 | 1.50 | 0.59 | Up | 0.017090 | 0.16 |
| RFX1 | NM_002918 | 1416.30 | 941.94 | 1.50 | 0.59 | Up | 0.047826 | 0.18 |
| UBA1 | NM_003334 | 1140.49 | 758.49 | 1.50 | 0.59 | Up | 0.025650 | 0.12 |
| SRC | NM_005417 | 158.47 | 105.37 | 1.50 | 0.59 | Up | 0.008616 | 0.10 |
| EHD1 | NM_006795 | 9247.99 | 6148.43 | 1.50 | 0.59 | Up | 0.017749 | 0.14 |
| ITPRIP | NM_033397 | 4827.55 | 3206.52 | 1.51 | 0.59 | Up | 0.007497 | 0.12 |
| LIMK1 | NM_002314 | 347.82 | 230.63 | 1.51 | 0.59 | Up | 0.009530 | 0.12 |
| TNRC18 | NM_001080495 | 1425.65 | 945.26 | 1.51 | 0.59 | Up | 0.014682 | 0.10 |
| BBX | NM_020235 | 1404.32 | 930.66 | 1.51 | 0.59 | Up | 0.041901 | 0.20 |
| REC8 | NM_001048205 | 1558.93 | 1032.06 | 1.51 | 0.60 | Up | 0.036326 | 0.20 |
| FRAT2 | NM_012083 | 14460.43 | 9570.51 | 1.51 | 0.60 | Up | 0.023387 | 0.14 |
| ZNF641 | NM_152320 | 456.99 | 302.28 | 1.51 | 0.60 | Up | 0.028318 | 0.10 |
| AFF1 | NM_005935 | 241.07 | 159.34 | 1.51 | 0.60 | Up | 0.032863 | 0.14 |
| PSAP | NM_001042465 | 13757.98 | 9087.50 | 1.51 | 0.60 | Up | 0.033009 | 0.16 |
| PLIN3 | NM_005817 | 455.49 | 300.66 | 1.51 | 0.60 | Up | 0.041854 | 0.16 |
| SMARCD3 | NM_003078 | 1593.17 | 1050.29 | 1.52 | 0.60 | Up | 0.012551 | 0.16 |
| CSNK1G2 | NM_001319 | 16865.90 | 11109.46 | 1.52 | 0.60 | Up | 0.036516 | 0.12 |
| WASF3 | NM_006646 | 48.88 | 32.19 | 1.52 | 0.60 | Up | 0.032010 | 0.18 |
| GUK1 | NM_000858 | 19244.34 | 12668.08 | 1.52 | 0.60 | Up | 0.035033 | 0.18 |
| NT5M | NM_020201 | 1539.60 | 1013.31 | 1.52 | 0.60 | Up | 0.033526 | 0.18 |
| YY1AP1 | NM_139118 | 2695.66 | 1773.74 | 1.52 | 0.60 | Up | 0.019937 | 0.14 |
| LCP1 | NM_002298 | 42127.01 | 27719.38 | 1.52 | 0.60 | Up | 0.005107 | 0.08 |
| LRRN2 | NM_201630 | 191.20 | 125.79 | 1.52 | 0.60 | Up | 0.028352 | 0.14 |
| BRD3 | NM_007371 | 415.67 | 273.40 | 1.52 | 0.60 | Up | 0.033932 | 0.18 |
| MOB3A | NM_130807 | 5957.92 | 3918.30 | 1.52 | 0.60 | Up | 0.022703 | 0.14 |
| VASP | NM_003370 | 11722.83 | 7709.49 | 1.52 | 0.60 | Up | 0.005976 | 0.06 |
| IQGAP1 | NM_003870 | 10967.14 | 7210.20 | 1.52 | 0.61 | Up | 0.001416 | 0.02 |
| RGS3 | NM_134427 | 1234.24 | 810.65 | 1.52 | 0.61 | Up | 0.005417 | 0.10 |
| SKIV2L | NM_006929 | 251.30 | 164.91 | 1.52 | 0.61 | Up | 0.043080 | 0.20 |
| IRF9 | NM_006084 | 3741.04 | 2454.71 | 1.52 | 0.61 | Up | 0.005976 | 0.10 |
| PSTPIP1 | NM_003978 | 5070.96 | 3324.06 | 1.53 | 0.61 | Up | 0.023325 | 0.14 |
| ARHGDIB | NM_001175 | 87318.28 | 57212.59 | 1.53 | 0.61 | Up | 0.012694 | 0.14 |
| PSMD2 | NM_002808 | 8033.14 | 5262.15 | 1.53 | 0.61 | Up | 0.049442 | 0.20 |
| IFITM2 | NM_006435 | 125829.65 | 82394.77 | 1.53 | 0.61 | Up | 0.049568 | 0.18 |
| CRAT | NM_000755 | 2292.20 | 1500.92 | 1.53 | 0.61 | Up | 0.016242 | 0.14 |
| CD300E | NM_181449 | 1636.57 | 1071.22 | 1.53 | 0.61 | Up | 0.004539 | 0.10 |
| TFE3 | NM_006521 | 628.17 | 411.15 | 1.53 | 0.61 | Up | 0.013533 | 0.12 |
| FHOD1 | NM_013241 | 590.39 | 386.16 | 1.53 | 0.61 | Up | 0.043994 | 0.22 |
| TRAFD1 | NM_001143906 | 4739.25 | 3099.57 | 1.53 | 0.61 | Up | 0.027516 | 0.18 |
| PTPRC | NM_002838 | 39023.82 | 25517.06 | 1.53 | 0.61 | Up | 0.025571 | 0.14 |
| WDFY4 | NM_020945 | 4574.22 | 2988.80 | 1.53 | 0.61 | Up | 0.020598 | 0.16 |
| TLN1 | NM_006289 | 658.02 | 429.62 | 1.53 | 0.62 | Up | 0.001808 | 0.06 |
| MEFV | NM_000243 | 627.29 | 408.90 | 1.53 | 0.62 | Up | 0.016228 | 0.18 |
| RALBP1 | NM_006788 | 909.87 | 593.08 | 1.53 | 0.62 | Up | 0.003566 | 0.08 |
| RALB | NM_002881 | 3573.45 | 2327.50 | 1.54 | 0.62 | Up | 0.034330 | 0.16 |
| ODF3B | NM_001014440 | 1452.92 | 946.02 | 1.54 | 0.62 | Up | 0.023947 | 0.16 |
| SLC43A2 | NM_152346 | 2517.20 | 1637.77 | 1.54 | 0.62 | Up | 0.019643 | 0.14 |
| NTNG2 | NM_032536 | 2073.58 | 1348.56 | 1.54 | 0.62 | Up | 0.036137 | 0.12 |
| PDGFA | NM_002607 | 74.03 | 48.10 | 1.54 | 0.62 | Up | 0.010320 | 0.14 |
| FTH1 | NM_002032 | 68397.05 | 44424.91 | 1.54 | 0.62 | Up | 0.001582 | 0.06 |
| F8A1 | NM_012151 | 5912.91 | 3838.26 | 1.54 | 0.62 | Up | 0.031958 | 0.16 |
| MBD6 | NM_052897 | 1716.34 | 1112.72 | 1.54 | 0.63 | Up | 0.009399 | 0.16 |
| WDR81 | NM_152348 | 1331.60 | 863.15 | 1.54 | 0.63 | Up | 0.033990 | 0.16 |
| CAMK1D | NM_020397 | 691.41 | 447.97 | 1.54 | 0.63 | Up | 0.045805 | 0.20 |
| RBPJ | NM_203284 | 2278.12 | 1475.22 | 1.54 | 0.63 | Up | 0.039188 | 0.20 |
| PKM2 | NM_182470 | 4246.63 | 2748.08 | 1.55 | 0.63 | Up | 0.009030 | 0.10 |
| CARS2 | NM_024537 | 13627.39 | 8816.83 | 1.55 | 0.63 | Up | 0.010369 | 0.12 |
| NEXN | NM_144573 | 196.54 | 127.12 | 1.55 | 0.63 | Up | 0.033888 | 0.22 |
| GIT2 | NM_057169 | 2261.20 | 1461.16 | 1.55 | 0.63 | Up | 0.048802 | 0.16 |
| CS | NM_004077 | 1408.67 | 909.95 | 1.55 | 0.63 | Up | 0.030505 | 0.16 |
| NFKB1 | NM_003998 | 1650.55 | 1065.96 | 1.55 | 0.63 | Up | 0.039449 | 0.16 |
| RNF19B | NM_153341 | 505.20 | 326.17 | 1.55 | 0.63 | Up | 0.011824 | 0.10 |
| IL6R | NM_000565 | 1315.87 | 849.46 | 1.55 | 0.63 | Up | 0.009036 | 0.10 |
| ATP6V0D1 | NM_004691 | 7935.11 | 5122.32 | 1.55 | 0.63 | Up | 0.040365 | 0.18 |
| PNKP | NM_007254 | 622.30 | 400.47 | 1.55 | 0.64 | Up | 0.038045 | 0.18 |
| PRR12 | NM_020719 | 394.87 | 254.03 | 1.55 | 0.64 | Up | 0.041370 | 0.22 |
| NBEAL2 | NM_015175 | 1990.02 | 1278.98 | 1.56 | 0.64 | Up | 0.031576 | 0.18 |
| TRIM56 | NM_030961 | 1563.02 | 1003.52 | 1.56 | 0.64 | Up | 0.032607 | 0.16 |
| SND1 | NM_014390 | 2948.64 | 1893.10 | 1.56 | 0.64 | Up | 0.019327 | 0.12 |
| INA | NM_032727 | 73.96 | 47.46 | 1.56 | 0.64 | Up | 0.026365 | 0.18 |
| CTSZ | NM_001336 | 1178.60 | 755.17 | 1.56 | 0.64 | Up | 0.011669 | 0.04 |
| SPATA13 | NM_153023 | 1441.65 | 923.04 | 1.56 | 0.64 | Up | 0.008671 | 0.08 |
| NLRP3 | NM_001079821 | 833.69 | 533.30 | 1.56 | 0.64 | Up | 0.045272 | 0.18 |
| SASH3 | NM_018990 | 4146.40 | 2650.76 | 1.56 | 0.65 | Up | 0.013822 | 0.14 |
| LYN | NM_002350 | 22722.02 | 14522.33 | 1.56 | 0.65 | Up | 0.012085 | 0.12 |
| FOXH1 | NM_003923 | 825.37 | 527.28 | 1.57 | 0.65 | Up | 0.049010 | 0.14 |
| NCOR2 | NM_006312 | 4137.39 | 2640.72 | 1.57 | 0.65 | Up | 0.029980 | 0.12 |
| AMICA1 | NM_153206 | 55523.07 | 35401.63 | 1.57 | 0.65 | Up | 0.017244 | 0.16 |
| ICAM1 | NM_000201 | 340.44 | 217.06 | 1.57 | 0.65 | Up | 0.031902 | 0.14 |
| TNFSF10 | NM_003810 | 4438.05 | 2827.05 | 1.57 | 0.65 | Up | 0.046020 | 0.20 |
| SLC45A4 | NM_001080431 | 673.23 | 428.83 | 1.57 | 0.65 | Up | 0.041845 | 0.20 |
| LCE1D | NM_178352 | 39163.82 | 24919.94 | 1.57 | 0.65 | Up | 0.021693 | 0.14 |
| ADAR | NM_001111 | 10454.67 | 6649.27 | 1.57 | 0.65 | Up | 0.016561 | 0.14 |
| MBOAT7 | NM_024298 | 10938.91 | 6953.37 | 1.57 | 0.65 | Up | 0.024181 | 0.16 |
| TLR4 | NM_138554 | 1160.82 | 736.69 | 1.58 | 0.66 | Up | 0.018655 | 0.12 |
| HBB | NM_000518 | 254838.20 | 161705.39 | 1.58 | 0.66 | Up | 0.024888 | 0.10 |
| INSL3 | NM_005543 | 785.59 | 498.42 | 1.58 | 0.66 | Up | 0.013141 | 0.12 |
| THRAP3 | NM_005119 | 5526.64 | 3500.71 | 1.58 | 0.66 | Up | 0.030155 | 0.18 |
| NAIP | NM_004536 | 226.69 | 143.47 | 1.58 | 0.66 | Up | 0.048446 | 0.20 |
| DNAJC5 | NM_025219 | 1284.93 | 811.49 | 1.58 | 0.66 | Up | 0.034610 | 0.16 |
| SLC16A3 | NM_001042422 | 18272.33 | 11527.80 | 1.59 | 0.66 | Up | 0.010773 | 0.14 |
| KPNB1 | NM_002265 | 9381.01 | 5914.30 | 1.59 | 0.67 | Up | 0.033156 | 0.18 |
| ACSS2 | NM_018677 | 496.80 | 312.87 | 1.59 | 0.67 | Up | 0.038412 | 0.24 |
| ITGAM | NM_000632 | 1599.19 | 1007.05 | 1.59 | 0.67 | Up | 0.043946 | 0.20 |
| TCIRG1 | NM_006019 | 8455.15 | 5324.26 | 1.59 | 0.67 | Up | 0.017393 | 0.16 |
| MXD1 | NM_002357 | 5507.07 | 3467.13 | 1.59 | 0.67 | Up | 0.006359 | 0.08 |
| FAM129B | NM_022833 | 1564.70 | 984.18 | 1.59 | 0.67 | Up | 0.044314 | 0.20 |
| ACTN1 | NM_001102 | 3339.62 | 2100.42 | 1.59 | 0.67 | Up | 0.017378 | 0.08 |
| PARP14 | NM_017554 | 2354.24 | 1479.44 | 1.59 | 0.67 | Up | 0.023638 | 0.10 |
| HOMER3 | NM_001145722 | 4250.05 | 2670.18 | 1.59 | 0.67 | Up | 0.042852 | 0.20 |
| C7orf74 | NM_175884 | 863.76 | 542.33 | 1.59 | 0.67 | Up | 0.021296 | 0.10 |
| ACSL1 | NM_001995 | 3984.27 | 2500.30 | 1.59 | 0.67 | Up | 0.049141 | 0.16 |
| PRR24 | NM_178511 | 936.76 | 587.62 | 1.59 | 0.67 | Up | 0.049433 | 0.22 |
| NARF | NM_001038618 | 2619.05 | 1642.27 | 1.59 | 0.67 | Up | 0.006142 | 0.08 |
| ZFP106 | NM_022473 | 1986.06 | 1244.64 | 1.60 | 0.67 | Up | 0.004114 | 0.08 |
| XPO6 | NM_015171 | 2640.65 | 1652.89 | 1.60 | 0.68 | Up | 0.024833 | 0.18 |
| KAT6A | NM_001099412 | 2362.09 | 1475.31 | 1.60 | 0.68 | Up | 0.028333 | 0.16 |
| ATF6 | NM_007348 | 923.33 | 576.22 | 1.60 | 0.68 | Up | 0.009355 | 0.10 |
| ZNF333 | NM_032433 | 422.64 | 263.51 | 1.60 | 0.68 | Up | 0.026973 | 0.12 |
| WWC3 | NM_015691 | 1328.68 | 828.14 | 1.60 | 0.68 | Up | 0.004092 | 0.10 |
| KCNQ3 | NM_004519 | 1639.06 | 1019.87 | 1.61 | 0.68 | Up | 0.041976 | 0.18 |
| CYFIP1 | NM_014608 | 350.24 | 217.92 | 1.61 | 0.68 | Up | 0.020483 | 0.12 |
| PF4 | NM_002619 | 11528.73 | 7171.72 | 1.61 | 0.68 | Up | 0.031326 | 0.18 |
| STAT1 | NM_139266 | 2695.71 | 1673.11 | 1.61 | 0.69 | Up | 0.032803 | 0.16 |
| CMA1 | NM_001836 | 111.05 | 68.80 | 1.61 | 0.69 | Up | 0.049348 | 0.20 |
| NT5C3 | NM_001002010 | 2632.41 | 1629.66 | 1.62 | 0.69 | Up | 0.002358 | 0.04 |
| ATP1A1 | NM_000701 | 1913.19 | 1184.16 | 1.62 | 0.69 | Up | 0.035995 | 0.16 |
| AQP1 | NM_198098 | 317.43 | 196.26 | 1.62 | 0.69 | Up | 0.032856 | 0.14 |
| MYO9B | NM_004145 | 2166.05 | 1338.92 | 1.62 | 0.69 | Up | 0.046896 | 0.18 |
| HSPA6 | NM_002155 | 2727.82 | 1685.90 | 1.62 | 0.69 | Up | 0.032081 | 0.20 |
| PLIN5 | NM_001013706 | 742.07 | 457.35 | 1.62 | 0.70 | Up | 0.015404 | 0.12 |
| SEC14L1 | NM_003003 | 10630.45 | 6549.01 | 1.62 | 0.70 | Up | 0.020094 | 0.14 |
| SLC19A1 | NM_194255 | 1161.76 | 715.70 | 1.62 | 0.70 | Up | 0.046627 | 0.20 |
| TBL1X | NM_005647 | 1992.02 | 1226.88 | 1.62 | 0.70 | Up | 0.018855 | 0.10 |
| LILRB3 | NM_006864 | 34230.72 | 21071.99 | 1.62 | 0.70 | Up | 0.017673 | 0.12 |
| DOK3 | NM_024872 | 1342.06 | 826.15 | 1.62 | 0.70 | Up | 0.004136 | 0.08 |
| TPM4 | NM_003290 | 865.65 | 532.57 | 1.63 | 0.70 | Up | 0.000916 | 0.00 |
| CASP10 | NM_032977 | 2637.60 | 1620.19 | 1.63 | 0.70 | Up | 0.008576 | 0.06 |
| PPP1R14B | NM_138689 | 5240.02 | 3216.74 | 1.63 | 0.70 | Up | 0.005970 | 0.10 |
| RERE | NM_012102 | 545.10 | 334.40 | 1.63 | 0.70 | Up | 0.029480 | 0.16 |
| NPLOC4 | NM_017921 | 3682.34 | 2258.06 | 1.63 | 0.71 | Up | 0.038708 | 0.24 |
| ASAP1 | NM_001247996 | 634.38 | 388.50 | 1.63 | 0.71 | Up | 0.021365 | 0.16 |
| LTA4H | NM_000895 | 7400.92 | 4532.10 | 1.63 | 0.71 | Up | 0.031190 | 0.18 |
| ZFP36L1 | NM_004926 | 18669.38 | 11432.09 | 1.63 | 0.71 | Up | 0.021248 | 0.08 |
| PLEKHO2 | NM_025201 | 4183.21 | 2560.35 | 1.63 | 0.71 | Up | 0.011817 | 0.12 |
| FBXO42 | NM_018994 | 1205.39 | 737.47 | 1.63 | 0.71 | Up | 0.028958 | 0.22 |
| DUX4 | NM_033178 | 301.69 | 184.53 | 1.63 | 0.71 | Up | 0.025958 | 0.16 |
| NOTCH2 | NM_024408 | 4018.21 | 2454.11 | 1.64 | 0.71 | Up | 0.011175 | 0.10 |
| ADAM8 | NM_001109 | 36566.74 | 22291.61 | 1.64 | 0.71 | Up | 0.044667 | 0.18 |
| CSF1R | NM_005211 | 9478.75 | 5776.85 | 1.64 | 0.71 | Up | 0.039695 | 0.18 |
| CCDC88B | NM_032251 | 13851.64 | 8423.84 | 1.64 | 0.72 | Up | 0.021952 | 0.14 |
| FES | NM_002005 | 1650.73 | 1003.81 | 1.64 | 0.72 | Up | 0.026951 | 0.16 |
| FSTL1 | NM_007085 | 116.56 | 70.83 | 1.65 | 0.72 | Up | 0.001315 | 0.06 |
| CSF3R | NM_156039 | 83814.93 | 50912.74 | 1.65 | 0.72 | Up | 0.013188 | 0.14 |
| HBA2 | NM_000517 | 270909.71 | 164490.33 | 1.65 | 0.72 | Up | 0.006251 | 0.10 |
| HSPA1A | NM_005345 | 6829.88 | 4145.54 | 1.65 | 0.72 | Up | 0.047086 | 0.18 |
| DYNC1H1 | NM_001376 | 1542.72 | 934.87 | 1.65 | 0.72 | Up | 0.031947 | 0.14 |
| FLOT2 | NM_004475 | 9375.44 | 5671.26 | 1.65 | 0.73 | Up | 0.015853 | 0.14 |
| CPAMD8 | NM_015692 | 446.79 | 270.15 | 1.65 | 0.73 | Up | 0.030917 | 0.16 |
| BAG1 | NM_001172415 | 2701.75 | 1633.55 | 1.65 | 0.73 | Up | 0.049692 | 0.20 |
| DISC1 | NM_001164554 | 623.17 | 376.72 | 1.65 | 0.73 | Up | 0.011193 | 0.12 |
| PHC2 | NM_198040 | 11442.48 | 6894.34 | 1.66 | 0.73 | Up | 0.028865 | 0.18 |
| IL2RG | NM_000206 | 7274.11 | 4374.34 | 1.66 | 0.73 | Up | 0.037674 | 0.20 |
| TMEM175 | NM_032326 | 3307.08 | 1986.70 | 1.66 | 0.74 | Up | 0.047940 | 0.22 |
| ACTN4 | NM_004924 | 927.55 | 556.43 | 1.67 | 0.74 | Up | 0.021202 | 0.08 |
| RUFY1 | NM_025158 | 4810.16 | 2884.48 | 1.67 | 0.74 | Up | 0.003718 | 0.10 |
| C10orf54 | NM_022153 | 17201.34 | 10311.31 | 1.67 | 0.74 | Up | 0.033434 | 0.18 |
| CTSB | NM_147780 | 2647.38 | 1586.28 | 1.67 | 0.74 | Up | 0.035514 | 0.16 |
| TFF1 | NM_003225 | 248.11 | 148.48 | 1.67 | 0.74 | Up | 0.044845 | 0.14 |
| DHRS13 | NM_144683 | 1300.55 | 777.72 | 1.67 | 0.74 | Up | 0.015784 | 0.12 |
| CSNK1D | NM_001893 | 667.48 | 398.88 | 1.67 | 0.74 | Up | 0.040689 | 0.14 |
| SPI1 | NM_001080547 | 11685.69 | 6982.22 | 1.67 | 0.74 | Up | 0.013799 | 0.16 |
| RNF24 | NM_007219 | 1068.69 | 637.40 | 1.68 | 0.75 | Up | 0.015192 | 0.16 |
| SLC8A1 | NM_021097 | 869.09 | 518.13 | 1.68 | 0.75 | Up | 0.018093 | 0.16 |
| TNFRSF1A | NM_001065 | 2563.82 | 1525.65 | 1.68 | 0.75 | Up | 0.040194 | 0.16 |
| THBS1 | NM_003246 | 206.27 | 122.68 | 1.68 | 0.75 | Up | 0.020440 | 0.08 |
| TLR5 | NM_003268 | 524.93 | 311.91 | 1.68 | 0.75 | Up | 0.031877 | 0.08 |
| EIF3B | NM_001037283 | 1809.90 | 1075.18 | 1.68 | 0.75 | Up | 0.040836 | 0.22 |
| HK1 | NM_033500 | 1487.58 | 882.19 | 1.69 | 0.75 | Up | 0.030768 | 0.16 |
| IL1RN | NM_173843 | 13126.44 | 7778.74 | 1.69 | 0.75 | Up | 0.025361 | 0.16 |
| PAQR6 | NM_024897 | 782.29 | 463.41 | 1.69 | 0.76 | Up | 0.004484 | 0.04 |
| SEC31A | NM_001077207 | 1164.86 | 689.78 | 1.69 | 0.76 | Up | 0.016235 | 0.12 |
| HSCB | NM_172002 | 53568.46 | 31714.55 | 1.69 | 0.76 | Up | 0.009619 | 0.14 |
| TMEM88 | NM_203411 | 609.64 | 360.61 | 1.69 | 0.76 | Up | 0.003788 | 0.06 |
| SBNO1 | NM_001167856 | 1146.23 | 677.96 | 1.69 | 0.76 | Up | 0.021235 | 0.18 |
| CD163 | NM_004244 | 1057.28 | 624.66 | 1.69 | 0.76 | Up | 0.037816 | 0.18 |
| EXTL3 | NM_001440 | 4228.43 | 2494.88 | 1.69 | 0.76 | Up | 0.024157 | 0.18 |
| TMEM40 | NM_018306 | 172.13 | 101.45 | 1.70 | 0.76 | Up | 0.038366 | 0.16 |
| C15orf39 | NM_015492 | 1475.85 | 869.75 | 1.70 | 0.76 | Up | 0.014883 | 0.12 |
| DAPK3 | NM_001348 | 2339.06 | 1373.73 | 1.70 | 0.77 | Up | 0.025044 | 0.18 |
| N4BP1 | NM_153029 | 656.29 | 385.28 | 1.70 | 0.77 | Up | 0.012116 | 0.10 |
| HBD | NM_000519 | 211465.49 | 123914.21 | 1.71 | 0.77 | Up | 0.007941 | 0.10 |
| FLOT1 | NM_005803 | 3197.19 | 1870.17 | 1.71 | 0.77 | Up | 0.001919 | 0.08 |
| SEC24C | NM_004922 | 6709.32 | 3924.19 | 1.71 | 0.77 | Up | 0.045696 | 0.20 |
| CLDN5 | NM_001130861 | 413.00 | 241.40 | 1.71 | 0.77 | Up | 0.003989 | 0.08 |
| CYP1B1 | NM_000104 | 1171.16 | 683.64 | 1.71 | 0.78 | Up | 0.013106 | 0.12 |
| APOL2 | NM_145637 | 6694.38 | 3906.78 | 1.71 | 0.78 | Up | 0.004048 | 0.10 |
| ETV6 | NM_001987 | 432.10 | 252.12 | 1.71 | 0.78 | Up | 0.018182 | 0.10 |
| KIAA1324 | NM_020775 | 1482.01 | 863.00 | 1.72 | 0.78 | Up | 0.047788 | 0.18 |
| YBX1 | NM_004559 | 8262.86 | 4810.22 | 1.72 | 0.78 | Up | 0.003515 | 0.00 |
| PRKCD | NM_006254 | 2785.50 | 1620.96 | 1.72 | 0.78 | Up | 0.047019 | 0.18 |
| ABCD1 | NM_000033 | 298.08 | 173.45 | 1.72 | 0.78 | Up | 0.006930 | 0.14 |
| TBL1Y | NM_033284 | 657.79 | 381.42 | 1.72 | 0.79 | Up | 0.009264 | 0.08 |
| CASP1 | NM_033292 | 3739.94 | 2162.92 | 1.73 | 0.79 | Up | 0.020365 | 0.16 |
| IL17RA | NM_014339 | 3134.96 | 1809.13 | 1.73 | 0.79 | Up | 0.015176 | 0.16 |
| KIAA0513 | NM_014732 | 6228.43 | 3588.72 | 1.74 | 0.80 | Up | 0.037013 | 0.16 |
| ARRB2 | NM_004313 | 14408.94 | 8288.74 | 1.74 | 0.80 | Up | 0.002837 | 0.10 |
| SLC15A3 | NM_016582 | 3443.63 | 1978.60 | 1.74 | 0.80 | Up | 0.018691 | 0.12 |
| ALDH2 | NM_000690 | 1438.95 | 826.06 | 1.74 | 0.80 | Up | 0.048240 | 0.18 |
| FKBP1B | NM_054033 | 412.33 | 236.29 | 1.75 | 0.80 | Up | 0.029222 | 0.14 |
| PRR5 | NM_015366 | 4709.51 | 2694.02 | 1.75 | 0.81 | Up | 0.022470 | 0.16 |
| PQLC2 | NM_001040125 | 169324.09 | 96789.77 | 1.75 | 0.81 | Up | 0.012787 | 0.12 |
| GLT1D1 | NM_144669 | 5174.61 | 2956.05 | 1.75 | 0.81 | Up | 0.023270 | 0.16 |
| GNAZ | NM_002073 | 962.23 | 547.29 | 1.76 | 0.81 | Up | 0.033819 | 0.12 |
| PLCB2 | NM_004573 | 17203.01 | 9772.05 | 1.76 | 0.82 | Up | 0.027097 | 0.18 |
| GIMAP8 | NM_175571 | 1210.23 | 687.29 | 1.76 | 0.82 | Up | 0.037779 | 0.20 |
| SLC25A44 | NM_014655 | 2372.80 | 1347.03 | 1.76 | 0.82 | Up | 0.018342 | 0.14 |
| DOCK8 | NM_203447 | 3667.62 | 2080.12 | 1.76 | 0.82 | Up | 0.035804 | 0.16 |
| SPARC | NM_003118 | 148.71 | 84.15 | 1.77 | 0.82 | Up | 0.017615 | 0.12 |
| ACADVL | NM_000018 | 5172.65 | 2923.68 | 1.77 | 0.82 | Up | 0.033586 | 0.20 |
| PXN | NM_002859 | 24928.45 | 13963.90 | 1.79 | 0.84 | Up | 0.010021 | 0.08 |
| PRDM1 | NM_001198 | 346.48 | 193.77 | 1.79 | 0.84 | Up | 0.003001 | 0.04 |
| UCP3 | NM_022803 | 2418.82 | 1352.48 | 1.79 | 0.84 | Up | 0.019892 | 0.12 |
| SORL1 | NM_003105 | 39630.29 | 22151.45 | 1.79 | 0.84 | Up | 0.008516 | 0.04 |
| CBS | NM_000071 | 457.27 | 255.07 | 1.79 | 0.84 | Up | 0.042958 | 0.20 |
| STAT2 | NM_005419 | 9108.43 | 5070.83 | 1.80 | 0.84 | Up | 0.009111 | 0.12 |
| S100A8 | NM_002964 | 31807.12 | 17662.57 | 1.80 | 0.85 | Up | 0.017149 | 0.12 |
| APOL1 | NM_145343 | 1765.94 | 980.27 | 1.80 | 0.85 | Up | 0.010160 | 0.16 |
| NAALADL2 | NM_207015 | 292.70 | 162.18 | 1.80 | 0.85 | Up | 0.044661 | 0.16 |
| ALOX5 | NM_000698 | 19620.14 | 10841.50 | 1.81 | 0.86 | Up | 0.029976 | 0.20 |
| PRIC285 | NM_001037335 | 4043.47 | 2232.34 | 1.81 | 0.86 | Up | 0.006325 | 0.12 |
| EMILIN2 | NM_032048 | 2866.84 | 1570.68 | 1.83 | 0.87 | Up | 0.026187 | 0.16 |
| SERPINA1 | NM_001002236 | 25780.78 | 14118.14 | 1.83 | 0.87 | Up | 0.006262 | 0.10 |
| APBB1IP | NM_019043 | 4365.52 | 2388.40 | 1.83 | 0.87 | Up | 0.009128 | 0.10 |
| NFE2 | NM_006163 | 23816.34 | 12988.62 | 1.83 | 0.87 | Up | 0.009484 | 0.10 |
| SECTM1 | NM_003004 | 13285.75 | 7243.48 | 1.83 | 0.88 | Up | 0.028894 | 0.24 |
| PGM1 | NM_002633 | 1138.38 | 618.64 | 1.84 | 0.88 | Up | 0.007047 | 0.10 |
| NEBL | NM_006393 | 72.89 | 39.50 | 1.85 | 0.88 | Up | 0.006178 | 0.08 |
| TALDO1 | NM_006755 | 38655.02 | 20922.60 | 1.85 | 0.89 | Up | 0.027461 | 0.14 |
| APP | NM_000484 | 320.84 | 173.60 | 1.85 | 0.89 | Up | 0.018333 | 0.14 |
| PARP9 | NM_001146106 | 1251.90 | 677.35 | 1.85 | 0.89 | Up | 0.015467 | 0.16 |
| NKX1-2 | NM_001146340 | 20195.28 | 10913.62 | 1.85 | 0.89 | Up | 0.025293 | 0.20 |
| CR1L | NM_175710 | 182.45 | 97.99 | 1.86 | 0.90 | Up | 0.000480 | 0.00 |
| ITGB5 | NM_002213 | 185.03 | 98.88 | 1.87 | 0.90 | Up | 0.027699 | 0.12 |
| SBNO2 | NM_014963 | 7369.77 | 3936.22 | 1.87 | 0.90 | Up | 0.011471 | 0.14 |
| OSCAR | NM_206818 | 3873.02 | 2066.61 | 1.87 | 0.91 | Up | 0.008347 | 0.08 |
| PADI2 | NM_007365 | 742.42 | 393.53 | 1.89 | 0.92 | Up | 0.033171 | 0.16 |
| EIF2AK2 | NM_002759 | 1695.38 | 894.60 | 1.90 | 0.92 | Up | 0.048544 | 0.16 |
| UBN1 | NM_016936 | 2989.00 | 1567.91 | 1.91 | 0.93 | Up | 0.024002 | 0.10 |
| SORT1 | NM_002959 | 786.11 | 412.28 | 1.91 | 0.93 | Up | 0.024380 | 0.18 |
| MYH9 | NM_002473 | 5612.75 | 2922.47 | 1.92 | 0.94 | Up | 0.002444 | 0.02 |
| MARCH2 | NM_016496 | 628.65 | 325.17 | 1.93 | 0.95 | Up | 0.031317 | 0.10 |
| SIPA1L1 | NM_015556 | 1829.57 | 944.99 | 1.94 | 0.95 | Up | 0.012681 | 0.14 |
| TRIM25 | NM_005082 | 7637.30 | 3939.73 | 1.94 | 0.95 | Up | 0.014307 | 0.14 |
| IRF7 | NM_004031 | 8953.41 | 4593.46 | 1.95 | 0.96 | Up | 0.021837 | 0.16 |
| APOBR | NM_018690 | 4120.97 | 2107.06 | 1.96 | 0.97 | Up | 0.019892 | 0.14 |
| FAM129A | NM_052966 | 9308.00 | 4750.02 | 1.96 | 0.97 | Up | 0.019659 | 0.14 |
| SEPT14 | NM_207366 | 1228.61 | 625.18 | 1.97 | 0.97 | Up | 0.036576 | 0.12 |
| OSM | NM_020530 | 581.85 | 291.37 | 2.00 | 1.00 | Up | 0.041370 | 0.14 |
| SELL | NM_000655 | 31194.94 | 15602.46 | 2.00 | 1.00 | Up | 0.001100 | 0.04 |
| CLU | NM_001831 | 836.51 | 413.13 | 2.02 | 1.02 | Up | 0.042327 | 0.22 |
| GNL3L | NM_019067 | 5954.61 | 2934.43 | 2.03 | 1.02 | Up | 0.032513 | 0.20 |
| GBP2 | NM_004120 | 2880.78 | 1411.74 | 2.04 | 1.03 | Up | 0.009735 | 0.08 |
| SOCS3 | NM_003955 | 708.26 | 345.92 | 2.05 | 1.03 | Up | 0.047206 | 0.20 |
| MMP9 | NM_004994 | 9362.43 | 4569.53 | 2.05 | 1.03 | Up | 0.045560 | 0.20 |
| CTNNA1 | NM_001903 | 542.33 | 264.28 | 2.05 | 1.04 | Up | 0.030932 | 0.16 |
| CXCL5 | NM_002994 | 471.95 | 228.28 | 2.07 | 1.05 | Up | 0.045531 | 0.18 |
| MVP | NM_017458 | 23967.71 | 11503.56 | 2.08 | 1.06 | Up | 0.004570 | 0.08 |
| RNF222 | NM_001146684 | 1421.34 | 679.93 | 2.09 | 1.06 | Up | 0.033565 | 0.14 |
| GRN | NM_002087 | 58318.30 | 27740.08 | 2.10 | 1.07 | Up | 0.025838 | 0.16 |
| CD14 | NM_001174104 | 33659.36 | 16008.56 | 2.10 | 1.07 | Up | 0.033178 | 0.16 |
| MX2 | NM_002463 | 6188.26 | 2937.57 | 2.11 | 1.07 | Up | 0.015737 | 0.12 |
| CTTN | NM_005231 | 727.22 | 329.60 | 2.21 | 1.14 | Up | 0.026835 | 0.16 |
| MFN2 | NM_014874 | 4694.03 | 2117.57 | 2.22 | 1.15 | Up | 0.028773 | 0.20 |
| LIMK2 | NM_001031801 | 634.56 | 285.91 | 2.22 | 1.15 | Up | 0.018130 | 0.14 |
| RNF213 | NM_020914 | 358.46 | 161.02 | 2.23 | 1.15 | Up | 0.037702 | 0.18 |
| NRGN | NM_006176 | 6611.52 | 2943.17 | 2.25 | 1.17 | Up | 0.035553 | 0.12 |
| CRYAA | NM_000394 | 4714.92 | 2016.40 | 2.34 | 1.23 | Up | 0.032902 | 0.18 |
| ZAN | NM_173059 | 632.03 | 269.90 | 2.34 | 1.23 | Up | 0.042321 | 0.18 |
| TRANK1 | NM_014831 | 1860.47 | 784.18 | 2.37 | 1.25 | Up | 0.001344 | 0.02 |
| LRG1 | NM_052972 | 2263.10 | 922.80 | 2.45 | 1.29 | Up | 0.018121 | 0.16 |
| IFIT2 | NM_001547 | 1460.84 | 565.72 | 2.58 | 1.37 | Up | 0.031747 | 0.14 |
| CCR1 | NM_001295 | 7713.61 | 2898.24 | 2.66 | 1.41 | Up | 0.021703 | 0.12 |
| DYSF | NM_003494 | 1437.46 | 525.19 | 2.74 | 1.45 | Up | 0.014909 | 0.08 |
| MX1 | NM_002462 | 7612.81 | 2637.29 | 2.89 | 1.53 | Up | 0.038333 | 0.16 |
| ITGB3 | NM_000212 | 602.10 | 166.11 | 3.62 | 1.86 | Up | 0.039158 | 0.08 |
| MYOM2 | NM_003970 | 332.85 | 90.77 | 3.67 | 1.87 | Up | 0.037239 | 0.14 |
| HBZ | NM_005332 | 417.65 | 88.39 | 4.73 | 2.24 | Up | 0.013201 | 0.16 |
| SIRPB1 | NM_001135844 | 2527.12 | 497.45 | 5.08 | 2.34 | Up | 0.047447 | 0.16 |

**Table S4:** Over-representation analysis of target gene list.

| **PANTHER classification category** | **Number of genes** |  |  | **Over-/under-represented (+/−)** | **Fold Enrichment** | ***P-*value** |
| --- | --- | --- | --- | --- | --- | --- |
|  | **Reference list^a^** | **Target list^b^** | **Expected^c^** |  |  |  |
| **Pathways** |  |  |  |  |  |  |
| Apoptosis signaling pathway (P00006) | 119 | 10 | 1.68 | + | 5.96 | 1.54E-03 |
| JAK/STAT signaling pathway (P00038) | 17 | 4 | 0.24 | + | 16.69 | 1.78E-02 |
| Integrin signalling pathway (P00034) | 194 | 11 | 2.73 | + | 4.02 | 1.92E-02 |
| Angiogenesis (P00005) | 174 | 10 | 2.45 | + | 4.08 | 3.50E-02 |
|  |  |  |  |  |  |  |
| **Molecular functions** |  |  |  |  |  |  |
| protein binding (GO:0005515) | 11103 | 227 | 156.48 | + | 1.45 | 6.83E-14 |
| binding (GO:0005488) | 14579 | 260 | 205.47 | + | 1.27 | 2.21E-10 |
| receptor binding (GO:0005102) | 1547 | 55 | 21.8 | + | 2.52 | 7.11E-07 |
| cell adhesion molecule binding (GO:0050839) | 454 | 27 | 6.4 | + | 4.22 | 1.73E-06 |
| molecular_function (GO:0003674) | 17318 | 275 | 244.08 | + | 1.13 | 4.39E-04 |
| protein complex binding (GO:0032403) | 771 | 31 | 10.87 | + | 2.85 | 7.02E-04 |
| macromolecular complex binding (GO:0044877) | 964 | 34 | 13.59 | + | 2.5 | 3.43E-03 |
| enzyme binding (GO:0019899) | 1887 | 53 | 26.6 | + | 1.99 | 3.44E-03 |
| cadherin binding (GO:0045296) | 295 | 17 | 4.16 | + | 4.09 | 4.48E-03 |
| cytoskeletal protein binding (GO:0008092) | 870 | 31 | 12.26 | + | 2.53 | 8.60E-03 |
| carbohydrate derivative binding (GO:0097367) | 2254 | 58 | 31.77 | + | 1.83 | 1.52E-02 |
| cytokine binding (GO:0019955) | 97 | 9 | 1.37 | + | 6.58 | 3.85E-02 |
| actin filament binding (GO:0051015) | 151 | 11 | 2.13 | + | 5.17 | 4.03E-02 |
|  |  |  |  |  |  |  |
| **Biological Processes** |  |  |  |  |  |  |
| immune response (GO:0006955) | 1662 | 95 | 23.42 | + | 4.06 | 3.46E-29 |
| immune system process (GO:0002376) | 2528 | 115 | 35.63 | + | 3.23 | 5.93E-28 |
| immune effector process (GO:0002252) | 1014 | 67 | 14.29 | + | 4.69 | 2.52E-22 |
| cell activation (GO:0001775) | 1025 | 64 | 14.45 | + | 4.43 | 8.01E-20 |
| secretion by cell (GO:0032940) | 956 | 60 | 13.47 | + | 4.45 | 2.01E-18 |
| regulated exocytosis (GO:0045055) | 684 | 51 | 9.64 | + | 5.29 | 3.12E-18 |
| response to stress (GO:0006950) | 3318 | 114 | 46.76 | + | 2.44 | 3.12E-17 |
| cell activation involved in immune response (GO:0002263) | 609 | 47 | 8.58 | + | 5.48 | 4.12E-17 |
| secretion (GO:0046903) | 1061 | 61 | 14.95 | + | 4.08 | 6.36E-17 |
| exocytosis (GO:0006887) | 768 | 52 | 10.82 | + | 4.8 | 7.59E-17 |
| myeloid leukocyte activation (GO:0002274) | 564 | 45 | 7.95 | + | 5.66 | 8.71E-17 |
| leukocyte activation (GO:0045321) | 878 | 55 | 12.37 | + | 4.44 | 1.63E-16 |
| leukocyte activation involved in immune response (GO:0002366) | 605 | 46 | 8.53 | + | 5.39 | 2.02E-16 |
| response to stimulus (GO:0050896) | 7914 | 189 | 111.54 | + | 1.69 | 6.53E-16 |
| myeloid leukocyte mediated immunity (GO:0002444) | 517 | 42 | 7.29 | + | 5.76 | 1.01E-15 |
| neutrophil mediated immunity (GO:0002446) | 496 | 41 | 6.99 | + | 5.87 | 1.56E-15 |
| myeloid cell activation involved in immune response (GO:0002275) | 513 | 41 | 7.23 | + | 5.67 | 5.05E-15 |
| single-organism transport (GO:0044765) | 2552 | 94 | 35.97 | + | 2.61 | 5.19E-15 |
| defense response (GO:0006952) | 1211 | 62 | 17.07 | + | 3.63 | 8.23E-15 |
| vesicle-mediated transport (GO:0016192) | 1770 | 76 | 24.95 | + | 3.05 | 9.08E-15 |
| single-organism localization (GO:1902578) | 2768 | 98 | 39.01 | + | 2.51 | 1.00E-14 |
| leukocyte degranulation (GO:0043299) | 504 | 40 | 7.1 | + | 5.63 | 1.78E-14 |
| neutrophil degranulation (GO:0043312) | 482 | 39 | 6.79 | + | 5.74 | 2.60E-14 |
| neutrophil activation involved in immune response (GO:0002283) | 483 | 39 | 6.81 | + | 5.73 | 2.79E-14 |
| neutrophil activation (GO:0042119) | 489 | 39 | 6.89 | + | 5.66 | 4.19E-14 |
| response to organic substance (GO:0010033) | 2641 | 94 | 37.22 | + | 2.53 | 4.99E-14 |
| granulocyte activation (GO:0036230) | 493 | 39 | 6.95 | + | 5.61 | 5.49E-14 |
| localization (GO:0051179) | 5456 | 145 | 76.9 | + | 1.89 | 1.83E-13 |
| leukocyte mediated immunity (GO:0002443) | 730 | 46 | 10.29 | + | 4.47 | 2.56E-13 |
| cellular response to organic substance (GO:0071310) | 1998 | 76 | 28.16 | + | 2.7 | 6.92E-12 |
| establishment of localization (GO:0051234) | 4515 | 124 | 63.63 | + | 1.95 | 2.24E-11 |
| innate immune response (GO:0045087) | 667 | 41 | 9.4 | + | 4.36 | 3.69E-11 |
| regulation of biological process (GO:0050789) | 11201 | 222 | 157.87 | + | 1.41 | 1.13E-10 |
| transport (GO:0006810) | 4384 | 120 | 61.79 | + | 1.94 | 1.15E-10 |
| cellular response to chemical stimulus (GO:0070887) | 2449 | 83 | 34.52 | + | 2.4 | 1.46E-10 |
| response to cytokine (GO:0034097) | 771 | 43 | 10.87 | + | 3.96 | 2.03E-10 |
| regulation of cellular process (GO:0050794) | 10543 | 211 | 148.59 | + | 1.42 | 1.13E-09 |
| biological regulation (GO:0065007) | 11809 | 227 | 166.43 | + | 1.36 | 1.47E-09 |
| cell surface receptor signaling pathway (GO:0007166) | 2227 | 76 | 31.39 | + | 2.42 | 1.94E-09 |
| regulation of immune system process (GO:0002682) | 1461 | 59 | 20.59 | + | 2.87 | 2.15E-09 |
| positive regulation of cellular process (GO:0048522) | 4859 | 125 | 68.48 | + | 1.83 | 2.34E-09 |
| regulation of response to stimulus (GO:0048583) | 3800 | 106 | 53.56 | + | 1.98 | 3.27E-09 |
| positive regulation of biological process (GO:0048518) | 5456 | 134 | 76.9 | + | 1.74 | 5.72E-09 |
| response to chemical (GO:0042221) | 3952 | 108 | 55.7 | + | 1.94 | 6.54E-09 |
| positive regulation of multicellular organismal process (GO:0051240) | 1474 | 57 | 20.77 | + | 2.74 | 3.34E-08 |
| regulation of multicellular organismal process (GO:0051239) | 2699 | 82 | 38.04 | + | 2.16 | 7.27E-08 |
| cellular response to stimulus (GO:0051716) | 6260 | 143 | 88.23 | + | 1.62 | 1.57E-07 |
| regulation of developmental process (GO:0050793) | 2289 | 73 | 32.26 | + | 2.26 | 1.66E-07 |
| negative regulation of biological process (GO:0048519) | 4806 | 119 | 67.74 | + | 1.76 | 1.92E-07 |
| inflammatory response (GO:0006954) | 459 | 29 | 6.47 | + | 4.48 | 2.61E-07 |
| single-organism process (GO:0044699) | 12759 | 233 | 179.82 | + | 1.3 | 2.78E-07 |
| biological_process (GO:0008150) | 17342 | 282 | 244.42 | + | 1.15 | 3.11E-07 |
| regulation of cytokine production (GO:0001817) | 602 | 33 | 8.48 | + | 3.89 | 4.20E-07 |
| cellular response to cytokine stimulus (GO:0071345) | 675 | 35 | 9.51 | + | 3.68 | 4.64E-07 |
| regulation of defense response (GO:0031347) | 753 | 37 | 10.61 | + | 3.49 | 5.59E-07 |
| cell communication (GO:0007154) | 5374 | 126 | 75.74 | + | 1.66 | 1.37E-06 |
| regulation of signal transduction (GO:0009966) | 2814 | 81 | 39.66 | + | 2.04 | 1.48E-06 |
| single organism signaling (GO:0044700) | 5279 | 124 | 74.4 | + | 1.67 | 1.94E-06 |
| signaling (GO:0023052) | 5283 | 124 | 74.46 | + | 1.67 | 2.04E-06 |
| signal transduction (GO:0007165) | 4888 | 117 | 68.89 | + | 1.7 | 2.91E-06 |
| single-multicellular organism process (GO:0044707) | 5565 | 128 | 78.43 | + | 1.63 | 3.23E-06 |
| cytokine-mediated signaling pathway (GO:0019221) | 479 | 28 | 6.75 | + | 4.15 | 3.29E-06 |
| regulation of signaling (GO:0023051) | 3169 | 86 | 44.66 | + | 1.93 | 6.26E-06 |
| negative regulation of cellular process (GO:0048523) | 4320 | 106 | 60.89 | + | 1.74 | 8.62E-06 |
| positive regulation of cell migration (GO:0030335) | 409 | 25 | 5.76 | + | 4.34 | 1.24E-05 |
| regulation of cell communication (GO:0010646) | 3116 | 84 | 43.92 | + | 1.91 | 1.49E-05 |
| regulation of cell death (GO:0010941) | 1596 | 54 | 22.49 | + | 2.4 | 1.69E-05 |
| regulation of immune response (GO:0050776) | 1023 | 41 | 14.42 | + | 2.84 | 1.95E-05 |
| response to external stimulus (GO:0009605) | 1846 | 59 | 26.02 | + | 2.27 | 2.15E-05 |
| single-organism cellular process (GO:0044763) | 9903 | 190 | 139.57 | + | 1.36 | 2.35E-05 |
| positive regulation of cell motility (GO:2000147) | 425 | 25 | 5.99 | + | 4.17 | 2.64E-05 |
| positive regulation of cell communication (GO:0010647) | 1586 | 53 | 22.35 | + | 2.37 | 3.73E-05 |
| regulation of innate immune response (GO:0045088) | 401 | 24 | 5.65 | + | 4.25 | 3.98E-05 |
| positive regulation of signaling (GO:0023056) | 1593 | 53 | 22.45 | + | 2.36 | 4.33E-05 |
| positive regulation of cellular component movement (GO:0051272) | 436 | 25 | 6.14 | + | 4.07 | 4.36E-05 |
| positive regulation of signal transduction (GO:0009967) | 1458 | 50 | 20.55 | + | 2.43 | 4.85E-05 |
| positive regulation of immune system process (GO:0002684) | 1015 | 40 | 14.31 | + | 2.8 | 4.98E-05 |
| platelet degranulation (GO:0002576) | 127 | 14 | 1.79 | + | 7.82 | 5.07E-05 |
| response to type I interferon (GO:0034340) | 69 | 11 | 0.97 | + | 11.31 | 5.55E-05 |
| positive regulation of response to stimulus (GO:0048584) | 2111 | 63 | 29.75 | + | 2.12 | 7.57E-05 |
| regulation of cellular component movement (GO:0051270) | 823 | 35 | 11.6 | + | 3.02 | 7.64E-05 |
| response to other organism (GO:0051707) | 824 | 35 | 11.61 | + | 3.01 | 7.87E-05 |
| response to external biotic stimulus (GO:0043207) | 827 | 35 | 11.66 | + | 3 | 8.61E-05 |
| regulation of cell motility (GO:2000145) | 751 | 33 | 10.58 | + | 3.12 | 9.74E-05 |
| positive regulation of cytokine production (GO:0001819) | 388 | 23 | 5.47 | + | 4.21 | 1.00E-04 |
| positive regulation of locomotion (GO:0040017) | 457 | 25 | 6.44 | + | 3.88 | 1.09E-04 |
| movement of cell or subcellular component (GO:0006928) | 1453 | 49 | 20.48 | + | 2.39 | 1.20E-04 |
| negative regulation of metabolic process (GO:0009892) | 2715 | 74 | 38.26 | + | 1.93 | 1.26E-04 |
| regulation of multicellular organismal development (GO:2000026) | 1742 | 55 | 24.55 | + | 2.24 | 1.27E-04 |
| regulation of apoptotic process (GO:0042981) | 1474 | 49 | 20.77 | + | 2.36 | 1.87E-04 |
| regulation of cell migration (GO:0030334) | 698 | 31 | 9.84 | + | 3.15 | 2.20E-04 |
| response to biotic stimulus (GO:0009607) | 861 | 35 | 12.13 | + | 2.88 | 2.31E-04 |
| regulation of locomotion (GO:0040012) | 821 | 34 | 11.57 | + | 2.94 | 2.39E-04 |
| cell migration (GO:0016477) | 864 | 35 | 12.18 | + | 2.87 | 2.51E-04 |
| regulation of programmed cell death (GO:0043067) | 1488 | 49 | 20.97 | + | 2.34 | 2.51E-04 |
| regulation of metabolic process (GO:0019222) | 6600 | 138 | 93.02 | + | 1.48 | 2.90E-04 |
| regulation of cellular metabolic process (GO:0031323) | 6090 | 130 | 85.83 | + | 1.51 | 3.03E-04 |
| regulation of cell differentiation (GO:0045595) | 1547 | 50 | 21.8 | + | 2.29 | 3.17E-04 |
| response to wounding (GO:0009611) | 558 | 27 | 7.86 | + | 3.43 | 3.40E-04 |
| regulation of biological quality (GO:0065008) | 3510 | 87 | 49.47 | + | 1.76 | 3.75E-04 |
| type I interferon signaling pathway (GO:0060337) | 65 | 10 | 0.92 | + | 10.92 | 3.77E-04 |
| cellular response to type I interferon (GO:0071357) | 65 | 10 | 0.92 | + | 10.92 | 3.77E-04 |
| regulation of response to external stimulus (GO:0032101) | 760 | 32 | 10.71 | + | 2.99 | 4.35E-04 |
| response to oxygen-containing compound (GO:1901700) | 1421 | 47 | 20.03 | + | 2.35 | 4.42E-04 |
| negative regulation of protein metabolic process (GO:0051248) | 1106 | 40 | 15.59 | + | 2.57 | 5.02E-04 |
| endocytosis (GO:0006897) | 609 | 28 | 8.58 | + | 3.26 | 5.38E-04 |
| defense response to other organism (GO:0098542) | 465 | 24 | 6.55 | + | 3.66 | 6.24E-04 |
| developmental process (GO:0032502) | 5478 | 119 | 77.21 | + | 1.54 | 7.16E-04 |
| negative regulation of macromolecule metabolic process (GO:0010605) | 2467 | 67 | 34.77 | + | 1.93 | 8.67E-04 |
| regulation of molecular function (GO:0065009) | 3077 | 78 | 43.37 | + | 1.8 | 1.00E-03 |
| single-organism developmental process (GO:0044767) | 5391 | 117 | 75.98 | + | 1.54 | 1.07E-03 |
| negative regulation of cellular metabolic process (GO:0031324) | 2430 | 66 | 34.25 | + | 1.93 | 1.11E-03 |
| negative regulation of response to stimulus (GO:0048585) | 1420 | 46 | 20.01 | + | 2.3 | 1.14E-03 |
| regulation of catalytic activity (GO:0050790) | 2492 | 67 | 35.12 | + | 1.91 | 1.26E-03 |
| regulation of intracellular signal transduction (GO:1902531) | 1767 | 53 | 24.9 | + | 2.13 | 1.27E-03 |
| locomotion (GO:0040011) | 1198 | 41 | 16.88 | + | 2.43 | 1.46E-03 |
| regulation of response to stress (GO:0080134) | 1395 | 45 | 19.66 | + | 2.29 | 1.79E-03 |
| positive regulation of metabolic process (GO:0009893) | 3181 | 79 | 44.83 | + | 1.76 | 1.89E-03 |
| multicellular organism development (GO:0007275) | 4768 | 106 | 67.2 | + | 1.58 | 1.91E-03 |
| multicellular organismal process (GO:0032501) | 6664 | 136 | 93.92 | + | 1.45 | 1.95E-03 |
| regulation of hemopoiesis (GO:1903706) | 321 | 19 | 4.52 | + | 4.2 | 1.98E-03 |
| regulation of peptidyl-tyrosine phosphorylation (GO:0050730) | 228 | 16 | 3.21 | + | 4.98 | 2.00E-03 |
| response to interferon-gamma (GO:0034341) | 148 | 13 | 2.09 | + | 6.23 | 2.34E-03 |
| regulation of cellular protein metabolic process (GO:0032268) | 2488 | 66 | 35.07 | + | 1.88 | 2.62E-03 |
| wound healing (GO:0042060) | 469 | 23 | 6.61 | + | 3.48 | 2.85E-03 |
| defense response to virus (GO:0051607) | 178 | 14 | 2.51 | + | 5.58 | 3.03E-03 |
| localization of cell (GO:0051674) | 960 | 35 | 13.53 | + | 2.59 | 3.04E-03 |
| cell motility (GO:0048870) | 960 | 35 | 13.53 | + | 2.59 | 3.04E-03 |
| regulation of protein metabolic process (GO:0051246) | 2743 | 70 | 38.66 | + | 1.81 | 4.39E-03 |
| positive regulation of molecular function (GO:0044093) | 1945 | 55 | 27.41 | + | 2.01 | 4.61E-03 |
| positive regulation of cellular metabolic process (GO:0031325) | 2970 | 74 | 41.86 | + | 1.77 | 4.68E-03 |
| regulation of inflammatory response (GO:0050727) | 375 | 20 | 5.29 | + | 3.78 | 4.78E-03 |
| positive regulation of developmental process (GO:0051094) | 1211 | 40 | 17.07 | + | 2.34 | 5.13E-03 |
| positive regulation of nitrogen compound metabolic process (GO:0051173) | 2868 | 72 | 40.42 | + | 1.78 | 5.30E-03 |
| anatomical structure development (GO:0048856) | 5113 | 110 | 72.06 | + | 1.53 | 5.38E-03 |
| myeloid cell differentiation (GO:0030099) | 190 | 14 | 2.68 | + | 5.23 | 6.50E-03 |
| positive regulation of macromolecule metabolic process (GO:0010604) | 2946 | 73 | 41.52 | + | 1.76 | 7.06E-03 |
| regulation of localization (GO:0032879) | 2562 | 66 | 36.11 | + | 1.83 | 7.41E-03 |
| regulation of primary metabolic process (GO:0080090) | 6042 | 124 | 85.16 | + | 1.46 | 8.33E-03 |
| positive regulation of defense response (GO:0031349) | 391 | 20 | 5.51 | + | 3.63 | 9.01E-03 |
| positive regulation of intracellular signal transduction (GO:1902533) | 918 | 33 | 12.94 | + | 2.55 | 9.15E-03 |
| regulation of nitrogen compound metabolic process (GO:0051171) | 5878 | 121 | 82.84 | + | 1.46 | 1.08E-02 |
| regulation of cell adhesion (GO:0030155) | 668 | 27 | 9.41 | + | 2.87 | 1.10E-02 |
| positive regulation of catalytic activity (GO:0043085) | 1643 | 48 | 23.16 | + | 2.07 | 1.14E-02 |
| cellular process (GO:0009987) | 14947 | 246 | 210.66 | + | 1.17 | 1.14E-02 |
| regulation of macromolecule metabolic process (GO:0060255) | 6080 | 124 | 85.69 | + | 1.45 | 1.18E-02 |
| response to virus (GO:0009615) | 264 | 16 | 3.72 | + | 4.3 | 1.33E-02 |
| system development (GO:0048731) | 4183 | 93 | 58.95 | + | 1.58 | 1.64E-02 |
| negative regulation of cellular protein metabolic process (GO:0032269) | 1038 | 35 | 14.63 | + | 2.39 | 1.77E-02 |
| regulation of myeloid cell differentiation (GO:0045637) | 178 | 13 | 2.51 | + | 5.18 | 1.78E-02 |
| negative regulation of signal transduction (GO:0009968) | 1141 | 37 | 16.08 | + | 2.3 | 2.16E-02 |
| regulation of anatomical structure morphogenesis (GO:0022603) | 957 | 33 | 13.49 | + | 2.45 | 2.22E-02 |
| multi-organism process (GO:0051704) | 2322 | 60 | 32.73 | + | 1.83 | 2.42E-02 |
| negative regulation of signaling (GO:0023057) | 1243 | 39 | 17.52 | + | 2.23 | 2.47E-02 |
| cell adhesion (GO:0007155) | 872 | 31 | 12.29 | + | 2.52 | 2.48E-02 |
| negative regulation of nitrogen compound metabolic process (GO:0051172) | 2276 | 59 | 32.08 | + | 1.84 | 2.71E-02 |
| cytokine production (GO:0001816) | 130 | 11 | 1.83 | + | 6 | 2.77E-02 |
| biological adhesion (GO:0022610) | 878 | 31 | 12.37 | + | 2.51 | 2.85E-02 |
| regulation of cellular component organization (GO:0051128) | 2338 | 60 | 32.95 | + | 1.82 | 3.01E-02 |
| negative regulation of cell death (GO:0060548) | 927 | 32 | 13.07 | + | 2.45 | 3.11E-02 |
| negative regulation of apoptotic process (GO:0043066) | 838 | 30 | 11.81 | + | 2.54 | 3.16E-02 |
| negative regulation of interleukin-12 production (GO:0032695) | 16 | 5 | 0.23 | + | 22.17 | 3.34E-02 |
| response to peptide (GO:1901652) | 427 | 20 | 6.02 | + | 3.32 | 3.34E-02 |
| negative regulation of defense response (GO:0031348) | 162 | 12 | 2.28 | + | 5.26 | 3.81E-02 |
| response to endogenous stimulus (GO:0009719) | 1271 | 39 | 17.91 | + | 2.18 | 4.15E-02 |
| positive regulation of cellular component biogenesis (GO:0044089) | 472 | 21 | 6.65 | + | 3.16 | 4.17E-02 |
| negative regulation of programmed cell death (GO:0043069) | 851 | 30 | 11.99 | + | 2.5 | 4.26E-02 |
| leukocyte migration (GO:0050900) | 361 | 18 | 5.09 | + | 3.54 | 4.44E-02 |
|  |  |  |  |  |  |  |
| **Cellular components** |  |  |  |  |  |  |
| cytoplasmic vesicle part (GO:0044433) | 1419 | 69 | 20 | + | 3.45 | 1.36E-16 |
| secretory granule (GO:0030141) | 825 | 52 | 11.63 | + | 4.47 | 2.48E-16 |
| secretory vesicle (GO:0099503) | 938 | 54 | 13.22 | + | 4.08 | 2.31E-15 |
| cytoplasmic part (GO:0044444) | 9354 | 206 | 131.83 | + | 1.56 | 3.73E-15 |
| cytoplasm (GO:0005737) | 11296 | 227 | 159.2 | + | 1.43 | 3.81E-13 |
| cytoplasmic vesicle (GO:0031410) | 2191 | 81 | 30.88 | + | 2.62 | 4.33E-13 |
| intracellular vesicle (GO:0097708) | 2194 | 81 | 30.92 | + | 2.62 | 4.68E-13 |
| cytoplasmic vesicle lumen (GO:0060205) | 336 | 28 | 4.74 | + | 5.91 | 1.48E-10 |
| vesicle lumen (GO:0031983) | 337 | 28 | 4.75 | + | 5.9 | 1.58E-10 |
| intracellular (GO:0005622) | 14487 | 258 | 204.18 | + | 1.26 | 2.99E-10 |
| vesicle (GO:0031982) | 4239 | 114 | 59.74 | + | 1.91 | 4.49E-10 |
| cytosol (GO:0005829) | 4883 | 125 | 68.82 | + | 1.82 | 5.23E-10 |
| ficolin-1-rich granule (GO:0101002) | 184 | 21 | 2.59 | + | 8.1 | 6.15E-10 |
| extracellular organelle (GO:0043230) | 2775 | 86 | 39.11 | + | 2.2 | 9.50E-10 |
| cell part (GO:0044464) | 16704 | 279 | 235.42 | + | 1.19 | 1.09E-09 |
| intracellular part (GO:0044424) | 14159 | 253 | 199.56 | + | 1.27 | 1.36E-09 |
| cell (GO:0005623) | 16731 | 279 | 235.8 | + | 1.18 | 1.54E-09 |
| extracellular exosome (GO:0070062) | 2758 | 85 | 38.87 | + | 2.19 | 1.85E-09 |
| secretory granule lumen (GO:0034774) | 320 | 26 | 4.51 | + | 5.76 | 2.02E-09 |
| extracellular vesicle (GO:1903561) | 2773 | 85 | 39.08 | + | 2.17 | 2.47E-09 |
| ficolin-1-rich granule lumen (GO:1904813) | 124 | 17 | 1.75 | + | 9.73 | 6.51E-09 |
| endomembrane system (GO:0012505) | 4298 | 112 | 60.58 | + | 1.85 | 6.81E-09 |
| extracellular space (GO:0005615) | 3755 | 102 | 52.92 | + | 1.93 | 9.36E-09 |
| extracellular region (GO:0005576) | 4752 | 119 | 66.97 | + | 1.78 | 1.38E-08 |
| intracellular organelle part (GO:0044446) | 8831 | 182 | 124.46 | + | 1.46 | 1.78E-08 |
| focal adhesion (GO:0005925) | 392 | 27 | 5.52 | + | 4.89 | 3.04E-08 |
| cell-substrate adherens junction (GO:0005924) | 395 | 27 | 5.57 | + | 4.85 | 3.59E-08 |
| extracellular region part (GO:0044421) | 3960 | 104 | 55.81 | + | 1.86 | 4.03E-08 |
| cell-substrate junction (GO:0030055) | 400 | 27 | 5.64 | + | 4.79 | 4.74E-08 |
| cellular_component (GO:0005575) | 18506 | 291 | 260.82 | + | 1.12 | 6.71E-08 |
| organelle part (GO:0044422) | 9041 | 183 | 127.42 | + | 1.44 | 8.50E-08 |
| intracellular organelle (GO:0043229) | 12358 | 227 | 174.17 | + | 1.3 | 1.05E-07 |
| anchoring junction (GO:0070161) | 517 | 30 | 7.29 | + | 4.12 | 1.38E-07 |
| membrane (GO:0016020) | 9539 | 189 | 134.44 | + | 1.41 | 1.84E-07 |
| adherens junction (GO:0005912) | 498 | 29 | 7.02 | + | 4.13 | 2.65E-07 |
| membrane-bounded organelle (GO:0043227) | 12364 | 225 | 174.26 | + | 1.29 | 5.82E-07 |
| organelle (GO:0043226) | 13347 | 235 | 188.11 | + | 1.25 | 3.37E-06 |
| cell leading edge (GO:0031252) | 368 | 23 | 5.19 | + | 4.43 | 5.92E-06 |
| whole membrane (GO:0098805) | 1591 | 53 | 22.42 | + | 2.36 | 6.43E-06 |
| lamellipodium (GO:0030027) | 181 | 16 | 2.55 | + | 6.27 | 1.39E-05 |
| organelle lumen (GO:0043233) | 5016 | 114 | 70.69 | + | 1.61 | 2.03E-05 |
| intracellular organelle lumen (GO:0070013) | 5016 | 114 | 70.69 | + | 1.61 | 2.03E-05 |
| membrane-enclosed lumen (GO:0031974) | 5016 | 114 | 70.69 | + | 1.61 | 2.03E-05 |
| cell junction (GO:0030054) | 1210 | 43 | 17.05 | + | 2.52 | 3.81E-05 |
| actin cytoskeleton (GO:0015629) | 462 | 24 | 6.51 | + | 3.69 | 8.59E-05 |
| contractile actin filament bundle (GO:0097517) | 57 | 9 | 0.8 | + | 11.2 | 2.24E-04 |
| stress fiber (GO:0001725) | 57 | 9 | 0.8 | + | 11.2 | 2.24E-04 |
| organelle membrane (GO:0031090) | 2880 | 73 | 40.59 | + | 1.8 | 4.61E-04 |
| actin filament bundle (GO:0032432) | 65 | 9 | 0.92 | + | 9.82 | 6.61E-04 |
| cell surface (GO:0009986) | 804 | 31 | 11.33 | + | 2.74 | 7.26E-04 |
| platelet alpha granule lumen (GO:0031093) | 67 | 9 | 0.94 | + | 9.53 | 8.48E-04 |
| tertiary granule (GO:0070820) | 163 | 13 | 2.3 | + | 5.66 | 1.06E-03 |
| actomyosin (GO:0042641) | 69 | 9 | 0.97 | + | 9.25 | 1.08E-03 |
| platelet alpha granule (GO:0031091) | 91 | 10 | 1.28 | + | 7.8 | 1.23E-03 |
| intracellular non-membrane-bounded organelle (GO:0043232) | 4007 | 91 | 56.47 | + | 1.61 | 1.39E-03 |
| non-membrane-bounded organelle (GO:0043228) | 4007 | 91 | 56.47 | + | 1.61 | 1.39E-03 |
| tertiary granule lumen (GO:1904724) | 55 | 8 | 0.78 | + | 10.32 | 2.00E-03 |
| bounding membrane of organelle (GO:0098588) | 1966 | 54 | 27.71 | + | 1.95 | 2.23E-03 |
| secretory granule membrane (GO:0030667) | 303 | 17 | 4.27 | + | 3.98 | 2.72E-03 |
| plasma membrane part (GO:0044459) | 2690 | 67 | 37.91 | + | 1.77 | 3.01E-03 |
| perinuclear region of cytoplasm (GO:0048471) | 653 | 26 | 9.2 | + | 2.83 | 3.54E-03 |
| plasma membrane bounded cell projection (GO:0120025) | 1825 | 50 | 25.72 | + | 1.94 | 6.24E-03 |
| podosome (GO:0002102) | 32 | 6 | 0.45 | + | 13.3 | 1.01E-02 |
| cell-cell junction (GO:0005911) | 420 | 19 | 5.92 | + | 3.21 | 1.46E-02 |
| cell projection (GO:0042995) | 1890 | 50 | 26.64 | + | 1.88 | 1.61E-02 |
| cytoskeleton (GO:0005856) | 2066 | 53 | 29.12 | + | 1.82 | 1.97E-02 |
| hemoglobin complex (GO:0005833) | 11 | 4 | 0.16 | + | 25.8 | 2.77E-02 |
| lytic vacuole (GO:0000323) | 654 | 24 | 9.22 | + | 2.6 | 3.23E-02 |
| lysosome (GO:0005764) | 654 | 24 | 9.22 | + | 2.6 | 3.23E-02 |
| membrane region (GO:0098589) | 368 | 17 | 5.19 | + | 3.28 | 3.28E-02 |
| intracellular membrane-bounded organelle (GO:0043231) | 10632 | 185 | 149.85 | + | 1.23 | 3.39E-02 |
| ruffle (GO:0001726) | 163 | 11 | 2.3 | + | 4.79 | 3.44E-02 |
| nuclear part (GO:0044428) | 4268 | 90 | 60.15 | + | 1.5 | 3.50E-02 |
| cell cortex part (GO:0044448) | 136 | 10 | 1.92 | + | 5.22 | 3.92E-02 |
| cell periphery (GO:0071944) | 5472 | 109 | 77.12 | + | 1.41 | 3.93E-02 |
| vacuole (GO:0005773) | 753 | 26 | 10.61 | + | 2.45 | 4.09E-02 |

Pathways. molecular functions. biological processes and cellular components resulted significantly over-represented by 292 protein coding genes.

^a^ Number of genes in the reference list that map to this PANTHER classification category.

^b^ Number of genes in the target genes list that map to this PANTHER classification category.

^C^ Expected value is the number of genes that could be expected in target genes list for this PANTHER category based on the reference list.
